# A cryptic binding pocket regulates the metal-dependent activity of Cas9

**DOI:** 10.1038/s41467-026-78458-0

**Published:** 2026-10-09

**Authors:** Mohd Ahsan, Alexa L. Knight, Aakash Saha, Delisa Ramos, Isabel Strohkendl, Erin Skeens, Madeline S. West, Jinping Luo, Charlotte S. Dresser, David W. Taylor, George P. Lisi, Giulia Palermo

**Affiliations:** 1 Department of Chemistry and Biochemistry, University of California Los Angeles, Los Angeles, CA USA; 2 Department of Molecular Biology, Cell Biology & Biochemistry, Brown University, Providence, RI USA; 3 Department of Bioengineering and Chemistry, University of California Riverside, Riverside, CA USA; 4 Department of Molecular Biosciences, University of Texas at Austin, Austin, TX USA; 5 Interdisciplinary Life Sciences Graduate Programs, University of Texas at Austin, Austin, TX USA; 6 Giuliani Center for Systems and Synthetic Biology, University of Texas at Austin, Austin, TX USA; 7 Giuliani RNA Center, Brown University, Providence, RI USA

**Keywords:** Computational biophysics, Enzyme mechanisms, CRISPR-Cas systems

## Abstract

Cas9 is a metal-dependent nuclease used for genome editing across diverse cells and organisms exhibiting distinct ionic environments, yet how metal ions regulate its catalytic function, and consequently its editing efficiency remains unclear. Here, molecular simulations, Markov state models, and mixed quantum–classical approaches reveal that divalent metals promote activation of the catalytic HNH domain through formation of a divalent metal-binding pocket (DBP) at the HNH–RuvC interface. This cryptic pocket emerges dynamically during HNH activation and is supported by NMR measurements revealing metal-dependent structural perturbations within the HNH–L2 region. Mutations targeting the DBP disrupt HNH activation and impair the coupled catalytic activity of both nucleases, identifying the pocket as a key regulator of Cas9’s metal-dependent activity. Together with quantum–classical simulations revealing distinct catalytic behaviours among divalent metals, these findings uncover an ion-dependent regulatory mechanism in Cas9 with insights for genome editing across diverse biological environments.

## Abstract (web summary)

Here the authors perform extensive molecular simulations, integrated with biochemical experiments and solution NMR, to show that a metal-binding pocket in Cas9 regulates its activation, revealing how metal-ion concentration influences the enzyme’s activity.

## Introduction

CRISPR-Cas9 (Clustered Regularly Interspaced Short Palindromic Repeats and associated protein 9) has revolutionized life sciences by introducing a facile genome editing technology^1,2^. At its core, Cas9 is a metal-dependent nuclease enabling precise genetic modifications across a wide range of cells and organisms with diverse ion uptake, metabolism, and intracellular metal homeostasis^3^. Intracellular availability of divalent ions such as Mg^2+^ and Ca^2+^ can vary substantially across organisms, cell types, and physiological conditions, owing to differences in ion transport, buffering, compartmentalization, and stress responses^4–7^. Such variations can influence protein-nucleic acid interactions and the specificity of Cas effectors^8–11^. However, the extent to which divalent metals regulate the catalytic function of Cas9, and consequently its genome editing capability in different cellular environments, remains unclear.

**Fig. 1:**
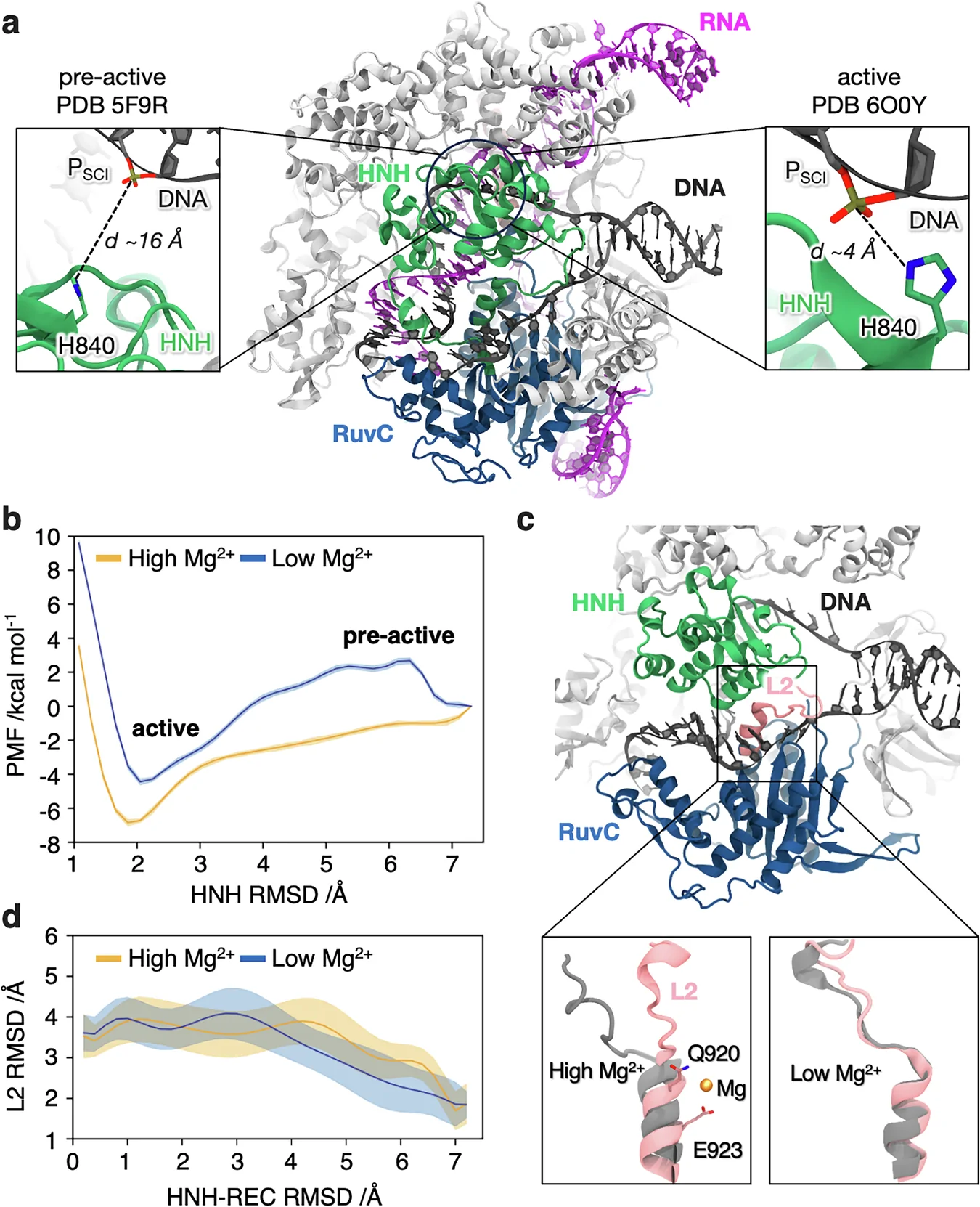
Cas9 activation under varying concentrations of Mg^2+^ ions. **a** Structure of *S. pyogenes* Cas9 bound to RNA (magenta) and DNA (black), highlighting the HNH (green) and RuvC (blue) catalytic domains^22^. Close-up views show the HNH nuclease in its pre-active state (PDB 5F9R^18^, left), where the catalytic H840 is ~16 Å away from the scissile phosphate (P_SCI_) on the DNA target strand, and in its active conformation (PDB 6O0Y^22^, right), where HNH is docked at the cleavage site. **b** Free energy profiles depicting the conformational change of HNH from its pre-active to active state at high (10 mM, yellow) and low (10 μM, blue) concentration of Mg^2+^. The Potential of Mean Force (PMF, in kcal/mol) was computed along the root-mean-square deviation (RMSD) of Cα atoms in the HNH domain and portions of the recognition lobe (REC) relative to both states. Shaded regions represent the standard error obtained using Monte Carlo bootstrap analysis of the free energy simulations (see Supplementary Methods). **c** Rearrangements of the L2 region (residues 907–925) in response to Mg^2+^ concentration. At high Mg^2+^ levels (left), residues Q920 and E923 form interactions with the ion, which are lost under low Mg^2+^ conditions (right). **d** RMSD of the L2 region along the free energy profiles reveals that as HNH moves toward the active state (i.e., in the transition region from RC ~ 7 to ~4 Å), conformational changes in L2 are more pronounced at high Mg^2+^ concentration. Shaded regions represent the standard deviation within each umbrella sampling window.

The *S. pyogenes* Cas9 (SpCas9) is an RNA-guided nuclease that utilizes the sequence specificity of its guide RNA (gRNA) to target and cleave complementary DNA sequences in the presence of metal ions^12^. Double-stranded DNA cleavage is carried out by two catalytic domains, HNH and RuvC (Fig. 1a), which cleave the DNA target strand (TS, complementary to the gRNA) and the non-target strand (NTS), respectively^3,13^. The HNH domain exhibits considerable conformational flexibility and structural rearrangements, in contrast to the RuvC domain, which maintains a stable structure^14^. Upon DNA binding, Cas9 undergoes a series of large-scale transitions, including DNA unwinding and R-loop formation, which have been shown to govern target recognition^15^. The HNH nuclease transitions from an inactive state, in which its catalytic triad (comprising H840, N863, D839) is oriented away from the cleavage site on the DNA TS^16,17^, to a pre-active state, where H840 shifts closer to the scissile phosphate but remains ~16 Å away (Fig. 1a, left)^18^. Biophysical and kinetic studies indicate that these early conformational rearrangements of the HNH domain can occur in the absence of divalent metal ions^19–21^. In contrast, the late-stage transition from the pre-active to the catalytically competent state – where H840 docks at the cleavage site (Fig. 1a, right)^22–24^ – is coupled to divalent ion binding. In particular, Mg^2+^ promotes the final conformational activation of the HNH domain, enabling alignment of the catalytic residues required for DNA cleavage^19–21^. Single-molecule studies have also shown that Mg^2+^ concentrations between 5 and 10 mM (i.e., high concentrations) promote the transition of the HNH domain into its active, docked state^20^. At low Mg^2+^ concentrations (10 μM), this conformational shift is hindered, limiting efficient HNH activation for DNA cleavage. These studies showed that other divalent cations, such as Ca^2+^ and Co^2+^, can promote this conformational activation when present at high concentrations, suggesting more generally that high concentrations of divalent cations may promote the docking of HNH at the DNA target strand for cleavage. However, the impact of ionic strength on this critical conformational change, which is essential for Cas9 function, remains unclear. Additionally, how different divalent metal ions – such as Mg^2+^, Ca^2+^, and Co^2+^ – alter the chemical mechanism of DNA cleavage, either facilitating or inhibiting catalysis^3^, remains an open question. Addressing these questions is key to understanding how Cas9 function is altered in cells with varying ion uptake, metabolism, and intracellular concentrations, as well as for optimizing Cas9-mediated genome editing across diverse biological contexts, including plants and mammalian cells.

Here, extensive molecular simulations, including free energy methods and Markov State Models, integrated with biochemical experiments and solution NMR, characterize the thermodynamics and kinetics of the HNH activation under varying concentrations of metals. We reveal that divalent metals facilitate HNH activation and the coordinated function of both nucleases by binding within a divalent metal binding pocket (DBP), which dynamically forms at the HNH-RuvC interfaces. Expanded analyses across monovalent, divalent, and trivalent ions further demonstrate distinct ion-dependent modulation of the HNH conformational landscape, indicating that Cas9 activation depends not simply on ionic strength but is preferentially associated with divalent ions.

Mutations within this cryptic DBP disrupt HNH dynamics and impair the coupled activity of both nucleases, underscoring its regulatory role in Cas9’s metal-dependent function. Quantum-classical simulations further elucidate the metal-specific nature of Cas9’s catalytic activity showing that while Mg^2+^ and Ca^2+^ support DNA cleavage, Co^2+^ disrupts enzymatic function, despite ionic strength promoting Cas9 conformational activation regardless of ion type. Together, these findings advance our understanding of Cas9’s metal-dependent mechanism and offer valuable insights for optimizing genome editing applications in diverse biological contexts, from mammalian cells with altered ion homeostasis to plants and microbes with atypical metal availability.

## Results

### Magnesium-dependent transition pathways of the HNH domain

The transition of the HNH domain from the pre-active (PDB 5F9R)^18^ to the active (PDB 6O0Y)^22^ state (Fig. 1a, Supplementary Fig. 1) under varying Mg^2+^ concentrations was investigated using a combination of free energy simulations and Markov State Model (MSM) analysis^25,26^, generating ~12 μs total sampling across systems. The Umbrella Sampling (US) method^27^ was employed to examine the effects of high (10 mM) and low (10 μM) Mg^2+^ concentrations on the energetics of the HNH transition, while a MSM approach provided the transition kinetics upon additional unbiased simulations (see Supplementary Methods). US simulations were performed using the difference in root-mean-square deviation (RMSD) of the Cα atoms in the HNH domain and portions of the recognition lobe relative to the pre-active and active states as the reaction coordinate (RC, Supplementary Fig. 2).

At high Mg^2+^ concentration, the transition from the pre-active to the active state proceeds barrierless, progressing toward the energetic minimum corresponding to the active state (Fig. 1b). At low Mg^2+^ concentration, a ~2.5 kcal/mol energetic barrier indicates a less favourable transition. The Potential of Mean Force (PMF) profile further indicates that the active state is less thermodynamically stable under low Mg^2+^ conditions, underscoring the role of Mg^2+^ in stabilizing the active state. The simulations also uncover structural rearrangements of the L2 region (residues 907–925), which bridges the HNH and RuvC domains (Fig. 1c)^18^. Indeed, as the HNH domain transitions toward the active state (from RC ~ 7 to ~4 Å), conformational changes in L2 are more pronounced at high Mg^2+^ levels (Fig. 1d) and are linked to the coordination of Mg^2+^ ions by residues Q920 and E923 (Fig. 1c, Supplementary Fig. 3).

To gain insights into the transition kinetics, a MSM was employed^25,26^. This approach describes the possible transitions between conformational states and their intermediates, along with the likelihood of these transitions over time. To construct our MSM, additional unbiased MD simulations were performed along the HNH conformational change, yielding ~3.6 μs of sampling for each Mg^2+^ concentration examined (see Supplementary Methods). The HNH conformational change was described using the Cα–Cα distances between S355 and S867, and between S867 and N1054, which served as independent components (ICs) for constructing our MSM. These distances, previously utilized in Förster Resonance Energy Transfer (FRET) experiments^28^, effectively represent the conformational activation of HNH^14^, and delineate the differences between the investigated states (Supplementary Figs. 4, 5).

**Fig. 2:**
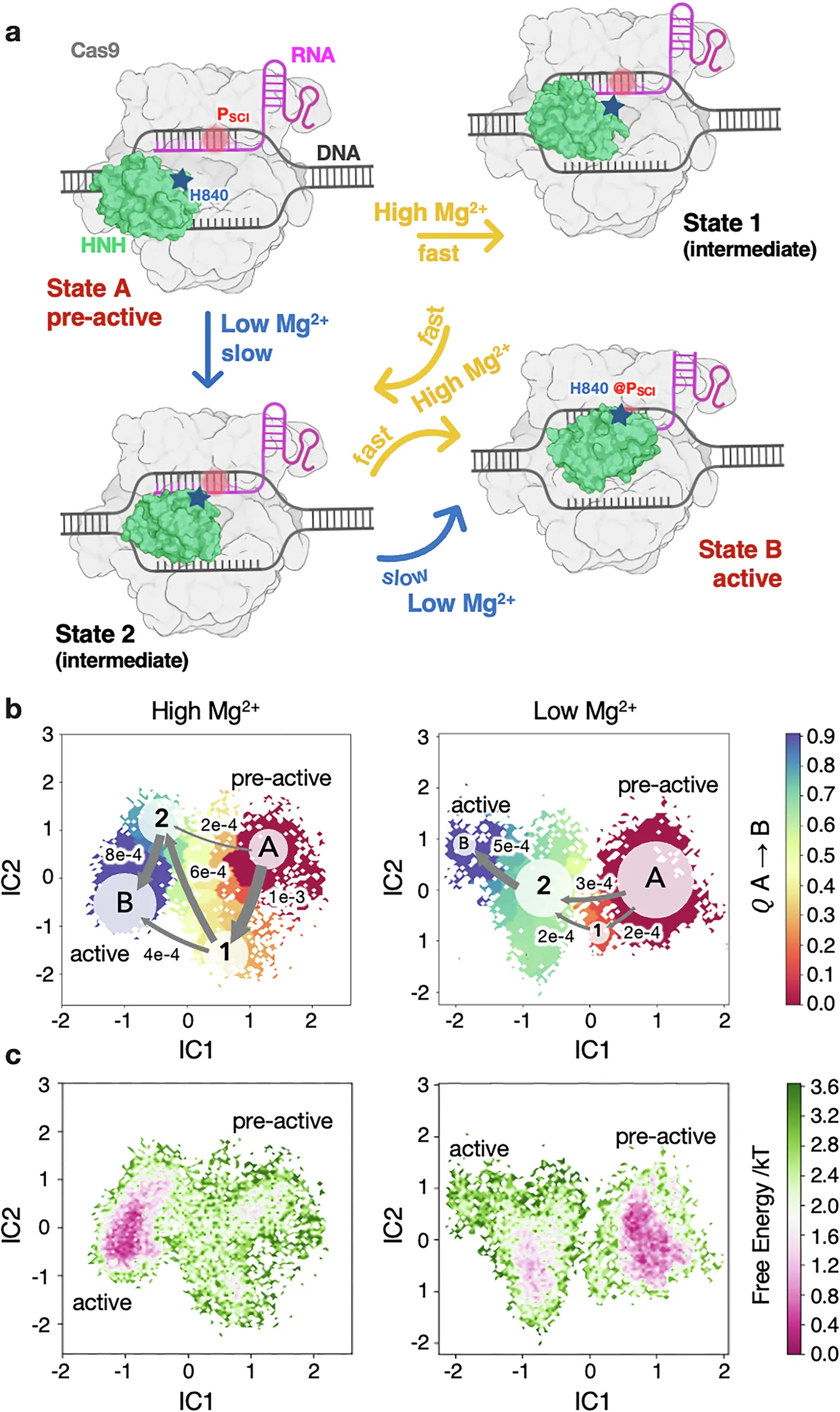
Kinetics of the HNH conformational change. **a** Schematic representation of the transition kinetics of the HNH domain from the pre-active (State A) to the active (State B) conformation via intermediate states (viz., States 1 and 2). The transition occurs approximately twice as fast (see panel **b**) at high Mg^2+^ concentration (10 mM) compared to low Mg^2+^ levels (10 μM). Created in BioRender (Palermo, G. (2026)). **b** Markov State Model (MSM) analysis depicting transitions between states under high (left) and low (right) Mg^2+^ conditions. Distances between residue pairs S355–S867 and S867–N1054 were used as input features for time-lagged independent component analysis (TICA)^58^, presented as scatter TICA plots (IC1−IC2 space) color-coded by the committor probability (Q; scale on the right). The Q values ~ 0.5 indicate that the system at the transition state, traversing two metastable states (State 1 and State 2) en route from the pre-active to the active state. Transition flux values show that the conformational change proceeds approximately twice as fast at high Mg^2+^ concentration. **c** TICA plots color-coded according to the free energy landscape, obtained by reweighting the Boltzmann distribution using stationary probabilities obtained from MSM at high (left) and low (right) Mg^2+^ concentrations. At high Mg^2+^, the active conformation is more frequently sampled and energetically favoured. In contrast, under low Mg^2+^ conditions, the system predominantly resides in the pre-active state.

Our MSM enabled a detailed characterization of the transition kinetics from the pre-active (State A) to the active state (State B, Fig. 2a). At both high and low Mg^2+^ levels, the transition is mediated by two intermediate metastable states (state 1 and state 2, Fig. 2b, Supplementary Figs. 6–8). At high Mg^2+^ concentration, the flux through the slowest transition step is twice as fast, relative to low Mg^2+^ concentration, reinforcing the critical role of Mg^2+^ in driving this transition. Importantly, while the MSM-derived kinetics remain faster than experimentally reported millisecond-timescale conformational interconversion rates^20^, the estimated rate differences correspond to modest deviations in the underlying free-energy barriers, within the expected uncertainty of atomistic force fields and MSM-based kinetic models^29,30^. Accordingly, MSM analysis is primarily used to characterize metastable conformational ensembles and ion-dependent structural transitions, rather than to quantitatively reproduce absolute experimental timescales^25,26^.

Analysis of the L2 region revealed marked conformational changes at high Mg^2+^ levels, with Q920 and E923 interacting with Mg^2+^ ions (Supplementary Fig. 9). The free energy landscapes from MSM analysis also show that at high Mg^2+^ levels, the active state (State B) is both energetically favoured and more frequently populated, indicating an efficient transition (Fig. 2c). At low Mg^2+^ concentration, the system is often trapped in intermediate metastable states, resulting in an incomplete transition to the active state. These findings demonstrate that elevated Mg^2+^ levels not only promote an effective transition to the active state but also stabilize it.

### Divalent ions drive the HNH transition through a cryptic binding pocket

**Fig. 3:**
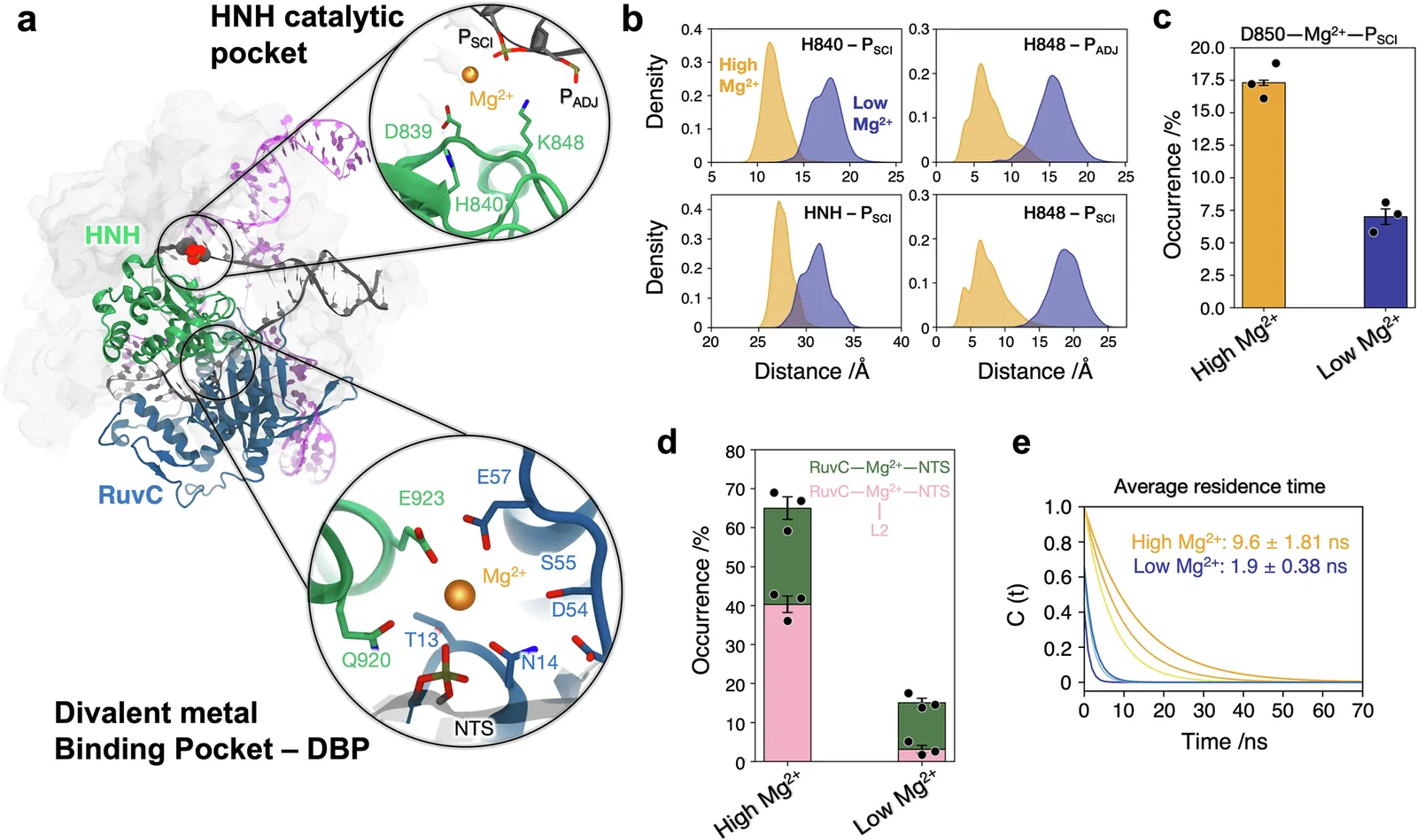
Dynamic formation of a cryptic divalent metal binding pocket during HNH activation. **a** Overview of two distinct divalent ion binding sites in Cas9. The HNH catalytic pocket (top inset) features a Mg^2+^ positioned between the catalytic core and the scissile phosphate (P_SCI_) on the DNA target strand (TS). A cryptic divalent metal binding pocket (DBP, bottom inset) emerges during molecular simulations at the HNH–RuvC interface. The DBP comprises residues from the RuvC domain, the L2 region, and the DNA non-target strand (NTS), with Mg^2+^ mediating bridging interactions among these regions. **b** Probability distribution of key distances bridging the HNH catalytic core and the TS at high (10 mM, yellow) and low (10 μM, blue) Mg^2+^ concentrations ([Mg^2+^]). Distances include those between residues H840 and K848 (Cα atoms) and P_SCI_ or the adjacent phosphate (P_ADJ_), as well as the distance between the centre of mass of HNH and P_SCI_. **c-d.** Percentage occurrence of Mg^2+^-mediated bridging interactions within the HNH catalytic site (**c**) and the DBP (**d**) at high and low [Mg^2+^]. In the catalytic site, bridging interactions involve Mg^2+^, D850, and P_SCI_. Within the DBP, Mg^2+^ interacts with the RuvC domain and the NTS (green), while simultaneously bridging the L2 region (pink). Bars represent the mean ± s.e.m. calculated from three independent MD simulation replicas (*n* = 3 independent simulations per condition). **e** Time correlation functions, (${C}_{t}$), used to determine Mg^2+^ residence times at high (yellow) and low (blue) Mg²⁺ concentrations. Lines represent three independent MD simulation replicas. Residence times were obtained by fitting each replica separately and are reported as mean ± s.e.m. across three independent simulations (*n* = 3 per condition). Data in panels (**b**–**e**) were computed over the full simulation ensemble.

To investigate the influence of divalent metals on the dynamics of HNH, all-atom MD simulations were performed under ionic conditions comparable to those used in in vitro experiments^19,20^, spanning high (10 mM) and low (10 μM) ionic concentrations. These simulations were initiated from the pre-active state of Cas9^18^ and conducted in three independent ~1 μs replicas for each condition, providing multi-microsecond sampling and a view of how varying ionic environments affect key molecular interactions. Mg^2+^ ions were observed to interact at two primary sites: between the HNH domain and the scissile phosphate (P_SCI_) on the DNA TS, and at the junction between HNH and RuvC (Fig. 3a). Under high Mg^2+^ concentrations, HNH displayed a more pronounced movement toward P_SCI_, evidenced by the reduction of key distances bridging the HNH catalytic core and the DNA TS (Fig. 3b, Supplementary Fig. 10). This observation is corroborated by the analysis of the backbone RMSD, highlighting that elevated Mg^2+^ concentrations enhance the dynamics of HNH while stabilizing RuvC (Supplementary Fig. 11). High Mg^2+^ concentrations also increased the frequency of bridging interactions between the ion, HNH (through coordination with D850) and P_SCI_ (Fig. 3c). Similar effects were observed in the presence of Ca^2+^ and Co^2+^ (Supplementary Fig. 12). Expanded simulations including monovalent, divalent, and trivalent ions further indicate that divalent cations favour sampling of activation-like HNH conformations (Supplementary Fig. 13). In contrast, the monovalent ion Li⁺ does not promote activation-like conformations, whereas the trivalent ion Al^3+^ disrupts coordinated HNH domain movement and increases conformational variability (details in Supplementary Results). Consistently, Cas9 DNA cleavage assays show efficient DNA cleavage activity in the presence of Mg^2+^, whereas Li⁺ and Al^3+^ do not support productive cleavage (Supplementary Fig. 14).

The second divalent ion binding site emerges as a pocket during molecular simulations, positioned between the HNH-RuvC interface and involving residues from the L2 region, the RuvC domain, and the NTS (Fig. 3a, bottom inset). This divalent ion Binding Pocket (DBP) recruits Mg^2+^ ions at high concentrations, forming interactions that bridge the L2 region with the RuvC domain and the NTS (Fig. 3d, Supplementary Fig. 15). Simulations employing different Mg^2+^ force-field parameters confirmed that these bridging interactions are more pronounced at high Mg^2+^ concentrations (Supplementary Fig. 16). Their formation at low Mg^2+^ concentration is hindered by shorter residence times, compared to high Mg^2+^ levels (Fig. 3e). Similar results were observed in the presence of Ca^2+^ and Co^2+^ (Supplementary Fig. 17), indicating that high concentrations of divalent ions facilitate the formation of bridging interactions within the DBP. Additional simulations performed at intermediate Mg^2+^ concentration (2 mM) show that the same conformational mechanism is preserved, with reduced DBP occupancy and fewer ion-mediated interactions relative to 10 mM (Supplementary Fig. 18).

Further MSM analysis of the high-Mg^2+^ simulation ensemble indicates that the DBP is transiently populated, with an estimated ensemble-level lifetime on the order of ~100 ns (Supplementary Fig. 19). Such transient conformational states are characteristic of cryptic pockets, namely dynamically formed cavities that may remain difficult to resolve in experimental structures despite their functional relevance^31–34^. The observed interactions also corroborate the rearrangement of the L2 region during the transition from the pre-active to active state (Fig. 1d), where L2 reorganization together with the formation of ion-mediated bridging interactions within the cryptic DBP promotes conformational activation of the HNH domain. Multiple sequence alignment of representative Cas9 orthologs reveals conservation of acidic and polar character within the DBP despite sequence variability, consistent with a local environment compatible with divalent ion binding (Supplementary Fig. 20).

**Fig. 4:**
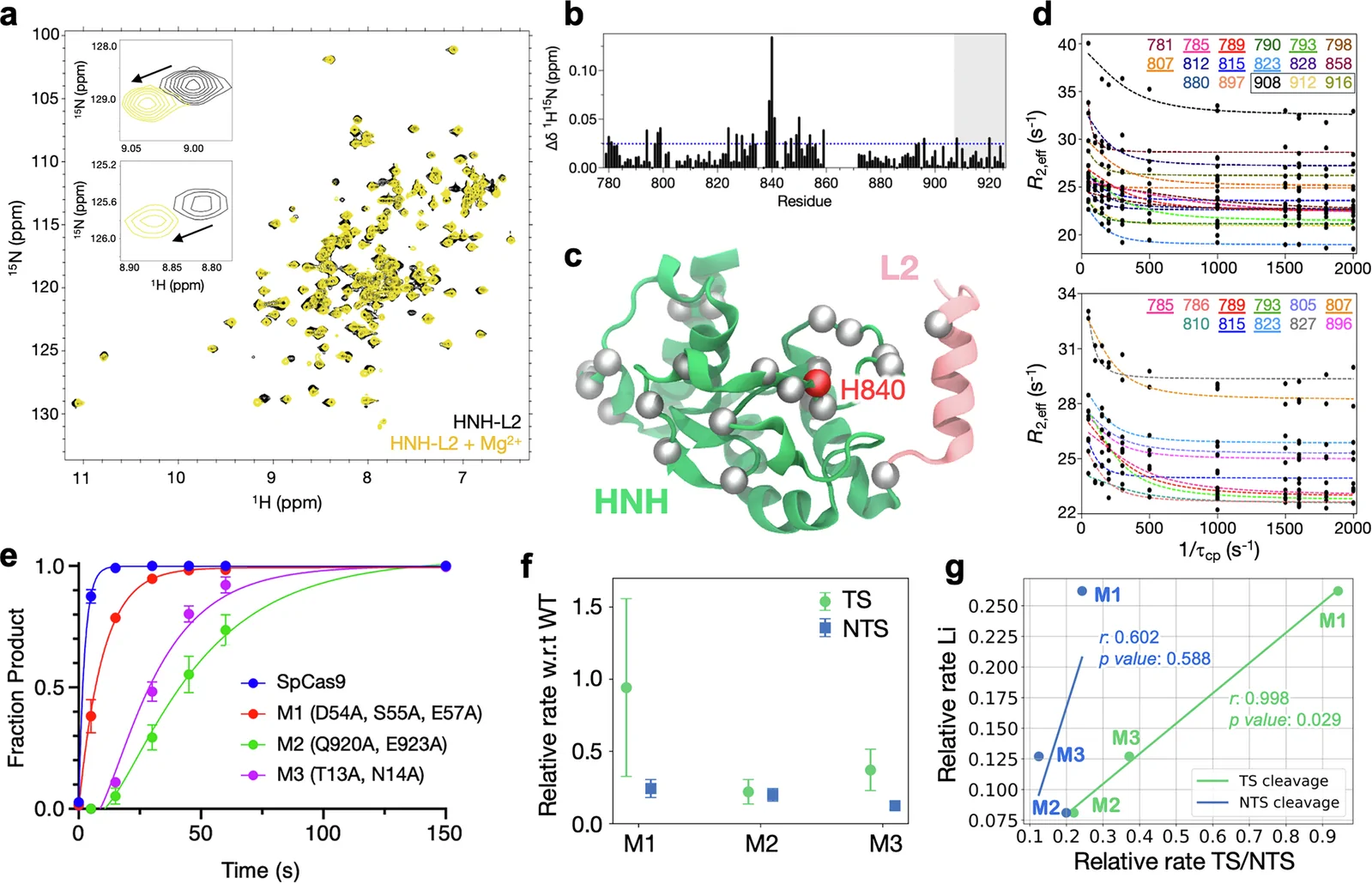
Experimental characterization of the cryptic divalent metal binding pocket in Cas9. **a** ^1^H-^15^N HSQC spectral overlay of apo HNH-L2 (black) and HNH-L2 bound to Mg^2+^ (gold). Representative chemical shift perturbations are inset. **b** ^1^H-^15^N combined chemical shift perturbations (Δδ) for Mg^2+^-bound HNH-L2 relative to apo. The grey area highlights L2 residues, the blue dashed line denotes 1.5σ above the 10% trimmed mean of all shifts. **c** Chemical shift perturbations >1.5σ are mapped onto the structure of HNH (green) and L2 (pink) in grey spheres. The red sphere denotes a significant perturbation at the catalytic H840. **d** CPMG relaxation dispersion profiles demonstrate slow timescale (μs - ms) motions in apo (upper) and Mg^2+^-bound (lower) HNH-L2. Mg^2+^ generally supresses motions in HNH-L2, evidenced by fewer curved profiles. Sites of flexibility shared between the samples are underlined, while sites of flexibility within L2 itself are boxed. **e** Cleavag**e** activity over time for plasmid DNA by wild-type Cas9 and variants with mutations in the DBP (M1: D54A/S55A/E57A, M2: Q920A/E923A, M3: T13A/N14A). Data points represent mean ± s.e.m. from three independent experiments (*n* = 3), fitted to a double-exponential curve. **f** Calculated rates of TS (green) and NTS (blue) cleavage relative to wild-type Cas9 (*k*_*obs*_, Supplementary Fig. 24). Rates were determined by fitting fraction-cleaved time courses obtained using fluorescently labelled oligoduplex substrates under saturating conditions. Symbols represent the relative fitted *k*_*obs*_ values, and error bars represent propagated standard errors from the fitted variant and WT *k*_*obs*_ values. **g** Correlation between the relative rate of appearance of Linear (product) species and the relative rate cleavage activity for both TS (green) and NTS (blue) cleavage with respect to WT SpCas9. Pearson’s r and two-sided *p* values are shown (*n* = 3 paired mutant observations). Details in Supplementary Figs. 23, 24.

To experimentally probe the ion-dependent conformational effects observed in the simulations, solution NMR experiments were performed using an isolated HNH–L2 construct, previously established as a suitable model for probing HNH conformational dynamics^35,36^. Mg^2+^ binding induces localized structural perturbations within the HNH–L2 (Fig. 4a), consistent with the ion-dependent conformational rearrangements observed in the simulations (Figs. 2 and 3). The largest perturbations are observed near the catalytic H840 and residues proximal to Q920 in L2 (Fig. 4b, c), supporting the involvement of these regions in the Mg^2+^-dependent regulatory interactions identified computationally. The divalent ion Ca^2+^ produces localized perturbations similar to Mg^2+^ (Supplementary Fig. 21). As expected, Mg^+2^-induced chemical shift perturbations within L2 are smaller than those at the HNH active site, likely due to the transient nature of the DBP. In contrast, the monovalent ion Li⁺ induces minimal perturbations even at high concentration, whereas the trivalent ion Al^3+^ induces broader spectral perturbations throughout the HNH–L2 region (details in Supplementary Results), suggesting that highly charged ions perturb the HNH conformational landscape differently from divalent ions. Fast timescale (ps–ns) flexibility of HNH-L2 measured by NMR spin relaxation is largely maintained with Mg^2+^, while μs–ms motions in L2 are attenuated by Mg^2+^, consistent with a binding interaction that stabilizes the L2 motif (Fig. 4d, Supplementary Fig. 22) and may contribute to the stabilization of the active state observed through MD simulations at high Mg^2+^ concentration (Fig. 3). NMR thereby indicates that Mg^2+^ ions alter structure and dynamics of HNH-L2, in line with computational studies. Together with the distinct behaviour observed for monovalent, divalent, or trivalent ions (Supplementary Fig. 21), these observations support ion-dependent modulation of the conformational landscape that cannot be solely explained by electrostatic stabilization within the DBP.

To biochemically validate these structural insights, mutations (mutants M1: D54A/S55A, E57A, M2: Q920A/E923A, M3: T13A/N14A) were introduced into residues lining the DBP, and the resulting plasmid DNA cleavage activity was compared to that of wild-type (WT) Cas9.

Loss of supercoiled plasmid substrate and appearance of linear product and nicked intermediate were fit to single and double exponential curves, respectively, to determine the apparent rates of the first and second strand cleavage events. All mutants exhibited reduced catalytic efficiency (Fig. 4e, Supplementary Fig. 23). Mutant M1 showed an equal reduction in the loss of supercoiled substrate and formation of linear product. Mutants M2 and M3, in addition to slower plasmid cleavage, also produced a prominent nicked intermediate species, suggesting that the sets of residues mutated within the DBP differ in magnitudes of effect on the RuvC and HNH domains. These observations indicate that the mutations impair cleavage mediated by the HNH and RuvC nuclease domains and suggest a disruption in the communication between them^15,28,37^. Additionally, we performed reactions with fluorescently labelled oligoduplex substrates, enabling separate measurements of DNA TS and NTS cleavage to quantify penalties to the HNH and RuvC domain, respectively (Supplementary Fig. 24). RuvC NTS cleavage (*k*_obs_) was uniformly reduced by ~75–85% across all mutants, while HNH TS cleavage (*k*_obs_) was reduced by ~6%, ~78%, and ~63% for mutants M1, M2, and M3, respectively (Fig. 4f). When compared to the formation of linearized product within the plasmid cleavage assay, there is a strong correlation with the relative rate of TS cleavage (Fig. 4g, r = 0.998), indicating that the different behaviours of the DBP mutants are due to the penalties in HNH cleavage. The observation that the M2 mutant, which includes alanine mutations of the L2 residues Q920 and E923, demonstrates the strongest reduction in HNH cleavage corroborates the role of the L2 region in HNH activation^23^.

Differential scanning fluorimetry (DSF) experiments performed on WT Cas9 and the M1–M3 variants under apo, Mg^2+^-bound, and Mg^2+^/gRNA-bound conditions show that all variants remain thermally stable, exhibiting melting transition temperatures comparable to WT Cas9 under all tested conditions (Supplementary Fig. 25, and Supplementary Table 1). Addition of gRNA stabilizes all variants by several degrees, consistent with preservation of the overall folded architecture and assembly competence of the Cas9 complex. Furthermore, temperature-dependent NMR measurements performed on the isolated HNH–L2 construct^35^ reveal preservation of the folded state, hydrogen bonding pattern, and general HNH-L2 ensemble across the investigated temperature range in both apo and Mg^2+^-bound conditions (Supplementary Fig. 26). Fluorescence anisotropy measurements further show that the M1–M3 variants retain DNA binding affinities comparable to WT Cas9 (Supplementary Fig. 27). In addition, gRNA binding measurements performed under low and high Mg^2+^ conditions reveal largely preserved guide RNA affinity across the variants, with only modest reductions observed for M2 (Supplementary Fig. 28). Together, these observations indicate that the DBP mutations do not substantially disrupt global protein stability or nucleic acid binding.

Additional MD simulations of the M1–M3 variants under high Mg^2+^ conditions further support this interpretation (Supplementary Fig. 29). In particular, the mutants reduce the occurrence of Mg^2+^-mediated bridging interactions within the DBP between the RuvC domain, the L2 region, and the NTS, with the M2 variant showing the strongest disruption of these interactions together with altered catalytic positioning of the HNH domain relative to P_SCI_. These results support the notion that perturbation of the DBP affects HNH conformational activation. Furthermore, these findings align with prior studies showing that the HNH and RuvC domains operate in a coupled catalytic manner^15,18,28,37,38^, relying on dynamic crosstalk essential for Cas9 activity. Collectively, these findings suggest that disruption of the DBP differentially modulates HNH catalysis depending on the mutation site, while communication between the HNH and RuvC domains remains impaired across all mutants, leading to a comparable reduction in RuvC catalysis.

Collectively, the computational, biochemical, and NMR data support a model in which the DBP contributes to the ion-dependent conformational activation of HNH and to the functional coupling between the HNH and RuvC domains of Cas9.

### Impact of divalent metal ions on target strand cleavage

**Fig. 5:**
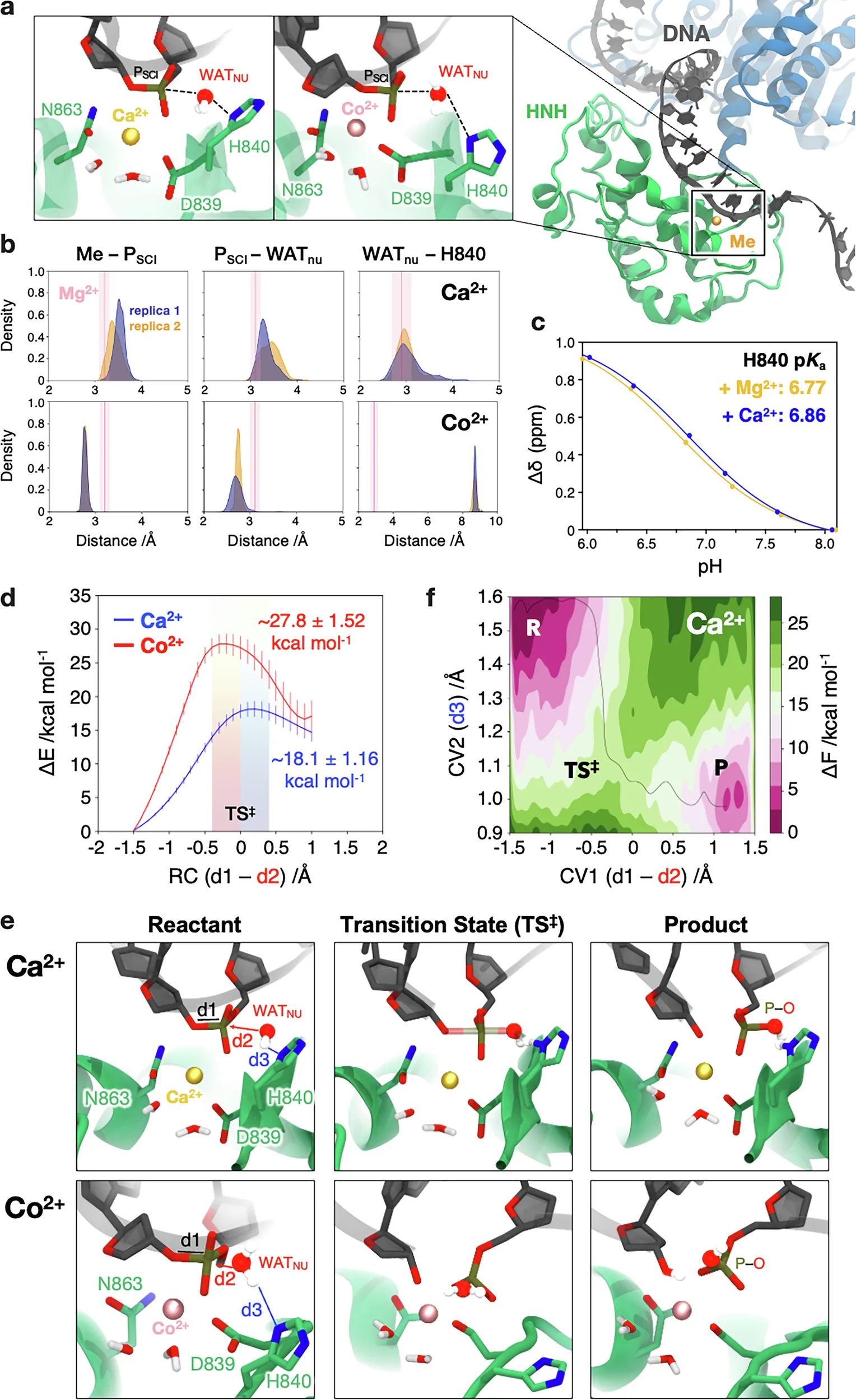
Effect of divalent metal ions on the HNH active site chemistry. **a** Conformations of the HNH active site in the presence of Ca^2+^ (left) and Co^2+^, obtained from classical molecular dynamics (MD) and ab initio QM(BLYP)/MM simulations. **b** Distributions of key distances involving the metal ion (Me), the scissile phosphate (P_SCI_), and the nucleophilic water molecule (WAT_nu_), computed from ~20 ps of QM/MM simulations (two replicas) with Ca^2+^ (top) and Co^2+^ (bottom). Red lines indicate the corresponding average distances obtained in the presence of Mg^2+^ using the same methodology; shaded regions represent the standard deviation. **c** NMR determined p*K*_a_ of the catalytic H840 in the presence of Mg^2+^ (yellow) and Ca^2+^ (blue). Errors in the chemical shift are smaller than the data points. The p*K*_a_ could not be quantified when bound to Co^2+^ due to extensive paramagnetic line broadening around the binding site. **d** Free energy profiles for phosphodiester bond cleavage in the presence of Ca^2+^ (blue) and Co^2+^ (red), obtained through QM(BLYP)/MM MD and thermodynamic integration. The difference in distance between the breaking and forming P–O bonds is used as the reaction coordinate (RC = d1 − d2; see panel **e**). Error bars represent standard deviations derived from error propagation analysis, with each data point reflecting the mean over the last ~6–10 ps of converged QM/MM MD. **e** Representative structures of the reactant (left), transition state (TS^**‡**^, centre), and product (right) during phosphodiester bond cleavage in the presence of Ca^2+^ (top) and Co^2+^ (bottom). **f** Two-dimensional free energy surface of phosphodiester bond cleavage in the presence of Ca^2+^, obtained from QM/MM metadynamics simulations. Free energy is projected onto two collective variables (CVs): CV1 describes nucleophilic attack on the scissile phosphate, whereas CV2 describes proton transfer from the water nucleophile to H840.

To investigate how different divalent metal ions affect TS cleavage, the active state of Cas9^22^ was simulated for ~9 μs in the presence of Mg^2+^, Ca^2+^, and Co^2+^, revealing their impact on the catalytic site’s structure and dynamics. The 2D-RMSD plots of the active site show that Ca^2+^ preserves a structure similar to that stabilized by Mg^2+^, whereas Co^2+^ induces pronounced structural deviations (Fig. 5a, Supplementary Fig. 30). To substantiate these observations, quantum mechanics/molecular mechanics (QM/MM) simulations were conducted, including the divalent ions, coordinating residues (D839, N863), the catalytic H840, critical DNA bases (G-3, T-4, C-5), and the surrounding waters in the QM region. For each system, ~20 ps of ab-initio MD at a Density Functional Theory (DFT) level were collected in two replicates. As a result, Ca^2+^ preserves the overall organization of the active site, similarly to Mg^2+^ (Fig. 5b, Supplementary Fig. 31). Consistently, NMR data show that binding of Ca^2+^ to the HNH–L2 construct induces structural perturbations similar to those observed for Mg^2+^ (Supplementary Fig. 21). Notably, previous biochemical studies have shown that, despite preserving HNH activation, Ca^2+^ remains functionally distinct from Mg^2+^ in supporting coordinated double-strand cleavage^12,20^. Furthermore, the p*K*_a_ of H840 remains largely unchanged in the presence of Mg^2+^ and Ca^2+^ (Fig. 5c, Supplementary Fig. 32), indicating that the effects of Mg^2+^ and Ca^2+^ within the HNH active site are comparable. In contrast, Co^2+^ binding results in a significant reduction in coordination distances, displacing H840 from P_SCI_ (Fig. 5b, Supplementary Fig. 31).

In the presence of Ca^2+^, the distances involving the ion, the P_SCI_ and the water nucleophile (WAT_nu_) – i.e., Ca^2+^–P_SCI_, P_SCI_–WAT_nu_, and WAT_nu_–H840 – are consistent with the reference Mg^2+^-bound system, preserving the active site’s structural integrity. Contrarily, with Co^2+^, shortened Co^2+^–P_SCI_ and P_SCI_–WAT_nu_ distances are accompanied by an increased WAT_nu_–H840 distance. This misalignment disrupts the catalytic geometry, potentially lowering the nucleophilicity of WAT_nu_ and diminishing its ability to facilitate DNA cleavage. To assess the impact of these structural differences on catalysis, we conducted free energy simulations, characterizing phosphodiester bond cleavage. A thermodynamic integration approach was combined with QM/MM simulations, using the difference in distance between the breaking and forming P–O bonds as a reaction coordinate. This methodology was previously employed to characterize the catalytic mechanism in the presence of Mg^2+^^39^. The free energy profiles indicate that Ca^2+^ supports catalysis with an activation barrier of ~18.1 ± 1.16 kcal/mol (Fig. 5d, Supplementary Fig. 33), resembling the barrier obtained for Mg^2+^ (~17.06 ± 1.22 kcal/mol) ^39^. Contrarily, Co^2+^ raises the activation barrier to ~27.8 ± 1.52 kcal/mol, indicating a less favourable catalytic pathway. This ~9 kcal/mol higher reaction barrier with Co^2+^ corroborates biochemical results showing an abolition of catalysis in the presence of Co^2+^^12,20^. A mechanistic analysis elucidates this difference, showing that while Ca^2+^ follows an associative S_N_2 mechanism (similar to Mg^2+^), Co^2+^ drives the reaction towards a dissociative mechanism due to the misalignment of H840 (Fig. 5e, Supplementary Figs. 33–35). Indeed, in the presence of Ca^2+^, H840 effectively abstracts a proton from WAT_nu_, facilitating the nucleophilic attack on P_SCI_ with simultaneous breaking of the O3’—P bond and forming the P—O_WAT_ bond. Conversely, in the presence of Co^2+^, a premature O3’—P bond cleavage is followed by a proton transfer from WAT_nu_ to O3’, resulting in a less efficient reaction pathway.

To further validate the catalytic mechanism with Ca^2+^, metadynamics simulations were performed (Fig. 5f, Supplementary Fig. 36), describing phosphodiester bond cleavage and deprotonation of the nucleophilic water across two dimensions. The free energy surface confirmed an S_N_2-like mechanism and an activation barrier of ~18.5 ± 0.3 kcal/mol, similar to that computed for Mg^2+^ using the same approach (~17.4 ± 0.84 kcal/mol)^39^, underscoring that divalent metals retaining the integrity of the active site preserve the catalysis.

## Discussion

Here, molecular simulations, solution NMR, and biochemical experiments were integrated to elucidate how divalent metal ions regulate conformational activation and target DNA cleavage in CRISPR–Cas9. Free energy simulations and Markov State Models reveal the thermodynamic and kinetic mechanisms underlying activation of the catalytic HNH domain under varying Mg^2+^ concentrations. We show that elevated Mg^2+^ levels (10 mM) facilitate the transition of HNH from the pre-active to the active conformation, accelerating the activation nearly twofold relative to low Mg^2+^ conditions (10 μM) (Figs. 1 and 2). These findings are in remarkable agreement with FRET experiments reporting an approximately 2.5-fold increase in transition rates at elevated Mg^2+^ concentrations^20^, and are further supported by kinetic studies indicating that Cas9 is unlikely to efficiently reach the active state in the absence of Mg^2+^^19^^,^. Consistently, single-molecule analyses have shown that HNH adopts distinct conformational ensembles depending on Mg^2+^ availability ^21^ and only rarely transitions into the active state in the absence of divalent cations^20^. Together, these observations provide strong experimental support for the mechanistic framework emerging from our simulations.

Our simulations reveal that the metal-dependent function of Cas9 is mediated by the formation of a divalent metal binding pocket (DBP) at the HNH–RuvC interface, involving residues from the L2 region, the RuvC domain, and the DNA non-target strand (NTS) (Fig. 3). Such cryptic pockets – transient cavities that remain hidden in static crystal or cryo-EM structures but emerge dynamically during conformational transitions – are increasingly recognized as important regulatory elements in biomolecular systems ^31–33^. Here, the DBP forms dynamically during HNH activation and modulates the conformational rearrangements required for catalytic activation. Elevated Mg^2+^ concentrations increase ion residence times within this pocket, promoting coordinated HNH motion toward the active state. Consistently, solution NMR experiments on an isolated HNH–L2 construct reveal Mg^2+^-dependent structural and dynamical perturbations near the catalytic H840 residue and within the L2 region (Fig. 4), supporting the involvement of this interface in the ion-dependent regulatory mechanism identified computationally. Similar behaviour is observed for Ca^2+^, Co^2+^, and other divalent cations (Supplementary Figs. 13, 21), which likewise facilitate activation-associated structural rearrangements at high concentrations. Together, these findings highlight a general mechanism by which divalent cations induce conformational activation through cryptic site stabilization.

Mutation of residues lining the DBP markedly reduces the cleavage activity of both HNH and RuvC and diminishes the accumulation of the intermediate nicked DNA species (Fig. 4), indicating that perturbation of this pocket disrupts the coordinated catalytic action of the two nuclease domains^15,18,28,37,38^. Mutations targeting distinct regions of the DBP – including M1 and M3 within RuvC and M2 within the L2 region of HNH – consistently impair RuvC cleavage activity and correlate with reduced formation of linear DNA products and diminished HNH catalysis (Fig. 4). Complementary MD simulations further show that these mutations disrupt Mg^2+^-mediated bridging interactions within the DBP and alter HNH positioning relative to the scissile phosphate (Supplementary Fig. 29), whereas NMR, DSF, and nucleic acid binding experiments (Supplementary Figs. 25–28) indicate that the variants retain overall structural integrity and substrate binding. Together, these findings support a model in which divalent ions stabilize transient interactions within the DBP, thereby promoting HNH activation and functional coupling between the HNH and RuvC nuclease domains.

Studies of the effects of Mg^2+^, Ca^2+^, and Co^2+^ on target-strand cleavage using quantum-classical simulations reveal distinct catalytic behaviours. Mg^2+^, the optimal catalytic ion, coordinates key residues and catalytic waters to promote DNA cleavage through an associative S_N_2 mechanism (Fig. 5)^39^. Ca^2+^ preserves a similar coordination geometry and supports target-strand cleavage through a comparable mechanism. In contrast, Co^2+^ induces substantial structural rearrangements within the active site, shifting the reaction toward a dissociative pathway and increasing the activation barrier, thereby impairing catalysis. Together with the distinct conformational responses observed for monovalent, divalent, and trivalent ions (Supplementary Figs. 13, 21), these findings indicate that Cas9 activation and catalysis depend not simply on ionic strength, but on ion-dependent modulation of the conformational and catalytic landscape by divalent metal ions.

In summary, this study provides key insights into how metal ions regulate the conformational activation and target DNA cleavage in CRISPR-Cas9. By integrating free energy simulations, Markov State Models, and quantum-classical approaches, biochemical and NMR experiments, we show that divalent metals such as Mg^2+^, Ca^2+^, and Co^2+^ promote the conformational activation of the catalytic HNH domain. These ions bind within a cryptic divalent metal-binding pocket (DBP), which emerges dynamically in simulations and plays a central role in stabilizing HNH conformations and coordinating its activity with RuvC. The DBP thus serves as a regulatory hub, mediating metal-dependent communication between the two nuclease domains. These findings uncover a regulatory mechanism in Cas9 function and offer key insights into the Cas9-mediated genome editing in cells and organisms where ion availability and metal homeostasis vary widely.

## Methods

### Molecular dynamics simulations

Molecular dynamics (MD) simulations were performed on two distinct structural states of CRISPR-Cas9: the pre-active conformation (PDB 5F9R, solved at 3.40 Å resolution^18^) and the catalytically active conformation (PDB 6O0Y, solved at 3.37 Å resolution^22^), representing two distinct states of the HNH domain. To assess ion-dependent conformational activation, simulations of the pre-active state were performed under high (10 mM) and low (10 μM) concentrations of Mg^2+^, Ca^2+^, and Co^2+^. These simulations included either 13 MCl_2_ units (M = Mg^2+^, Ca^2+^, or Co^2+^) corresponding to a high ion concentration of 10 mM, or a single MCl_2_ unit to represent a low concentration of 10 μM. These conditions were chosen to reflect the ionic regimes used in single-molecule and biochemical studies of Cas9^19,20^. The resulting systems comprised ~340,000 atoms within simulation boxes of ~180*120*140 Å^3^. MD simulations were performed using a protocol tailored for protein/nucleic acid complexes^40^ and applied in studies of CRISPR-Cas systems^41–45^. The Amber ff19SB^46^ force field was employed, incorporating the OL15^47^ and OL3^48^ corrections for DNA and RNA, respectively. The TIP3P^49^ model was used for water molecules. The Li & Merz model was used for ions^50,51^, describing the metal sites in agreement with QM/MM simulations (Supplementary Fig. 31). The 12-6-4 polarizable Li-Merz^52,53^ and the Allnér^54^ parameters were also used to model Mg^2+^ ions in the pre-active state. An integration time step of 2 fs was used. All systems were minimized and equilibrated through a staged heating protocol, gradually raising the temperature to 300 K under positional restraints that were progressively released. Production runs were conducted in the NVT ensemble. In addition to simulations performed with Mg^2+^, Ca^2+^, and Co^2+^ ions, expanded simulations were carried out on the pre-active state using additional monovalent, divalent, and trivalent ions (Li⁺, Be^2+^, Mn^2+^, Zn^2+^, Ra^2+^, and Al^3+^) under both high and low concentration conditions (full details are reported in the Supplementary Methods). Simulations of the pre-active state were also performed at an intermediate Mg^2+^ concentration (2 mM). Furthermore, the three mutants M1 (D54A/S55A/E57A), M2 (Q920A/E923A), and M3 (T13A/N14A) were simulated under high Mg^2+^ concentration conditions. All systems were simulated in three independent ~1 μs replicas. Altogether, these simulations resulted in over ~75 μs of aggregate MD sampling. The GPU-empowered version of AMBER 22^55^ was used as MD engine.

### Umbrella sampling simulations

The Umbrella sampling (US) free energy method was employed to characterize the conformational transition of the HNH domain from the pre-active to active state^27^. In this approach, a number of simulations (US windows) are run in parallel with additional harmonic bias potential applied to selected Reaction Coordinates (RCs):

1 $$V({RC})=\frac{k}{2}{({RC}\left(t\right)-{{RC}}^{*})}^{2}$$

where $V({RC})$ is the value of the bias potential, k is a bias force constant, ${RC}\left(t\right)$ is the value of RC at given time t and RC* is the reference value of RC. By using different RC* values in each US window, one can sample the biased probability distribution ${p}_{b}\left({RC}\right)$ along the whole RC range of interest. The difference in root-mean-square deviation (RMSD) of the Cα atoms in HNH and portions of REC (residues 175–393, 778–906) relative to the pre-activated and active states, was used as a RC (Supplementary Fig. 2a). Two independent US simulations were performed: under high (10 mM) and low (10 μm) concentrations of Mg^2+^. The systems were simulated in 36 overlapping windows, with the RC ranging from 7.4 Å to 0.2 Å, using a harmonic restraint with a spring constant of 50 kcal/mol·Å^2^. The centre of the harmonic bias potential was distributed along the RC in 36 windows, each separated by 0.2 Å, ensuring proper overlap of the probability distributions. For each system, ~120 ns-long trajectories were obtained per US window, resulting in ~4.3 µs of collective sampling per system (and a total of ~8.6 µs). The free energy profiles were computed using the Weighted Histogram Analysis (WHAM) method^56^, discarding the first ~20 ns of each window to ensure convergence. Error estimation on the free energy profile was performed using Monte Carlo bootstrap error analysis^57^. Details are reported in the Supplementary Methods.

Markov State Models. To resolve the kinetics of the HNH transition, Markov State Models (MSMs)^25,26^ were constructed using unbiased MD simulations initiated from 36 representative conformations extracted from the US trajectories. Each seeded trajectory was propagated for ~100 ns, totalling ~3.6 μs per condition. Features used for MSM construction included inter-residue Cα distances (S355–S867 and S867–N1054), previously validated by FRET studies. Time-lagged Independent Component Analysis (TICA)^58^ was performed (lag time = 5 ns), followed by k-means clustering into 100 microstates^59,60^, selected based on the saturation of the VAMP-2 score^61^. Implied timescales analysis^62^ confirmed a Markovian lag time of 5 ns, which was used to construct Bayesian MSMs. The Chapman–Kolmogorov test validated the models’ Markovianity^63^. Macrostates were defined using PCCA++ clustering^64^, identifying four metastable states. MSM-derived stationary distributions were used to reweight MD configurations, enabling the calculation of MSM-reweighted free energy landscapes projected onto the TICA space. Finally, Transition Path Theory (TPT)^65,66^ was applied to estimate the dominant flux pathways between the pre-active and active states based on forward and backward committor probabilities. MSM analysis was performed through the PyEMMA package^67^.

### QM/MM simulations

QM/MM simulations were performed on the active conformation of the HNH domain in the presence of Ca^2+^ and Co^2+^ in the active site. The QM region comprised the divalent ion, coordinating residues (D839, N863), the catalytic H840, DNA bases G-3, T-4, C-5, and nine water molecules, including the nucleophile and ion-coordinating waters. This region totalled 123 atoms and was capped with hydrogen atoms to saturate valence at the QM–MM boundary. H840 was modelled in its neutral ε-tautomer, consistent with prior QM/MM simulations including Mg^2+^^39^. QM atoms were treated at the DFT/BLYP^68,69^ level; the MM region used the same force field as in classical MD simulations (*vide supra*). Ab-initio MD simulations were carried out using the CPMD version 4 code^70^. The wave functions were expanded in a plane wave basis set up to a cutoff of 75 Ry. Interactions between the valence electrons and ionic cores were described using norm-conserving Martins-Troullier pseudopotentials^71^. Electrostatic coupling between periodic QM images was removed using Tuckerman’s scheme^72^. A Hamiltonian treatment of the electrostatic interaction between the QM and MM regions was used^73^. The QM/MM protocol included initial wavefunction optimization, followed by ~6 ps of Born–Oppenheimer MD at 300 K in NVT ensemble, using a 20 au (~0.48 fs) time step. This was followed by Car–Parrinello MD using a 5 au (~0.12 fs) timestep and a fictitious electron mass of 600 au. For each system, ~20 ps of unconstrained QM/MM simulations were performed. The active site conformations derived from these simulations were subsequently used as starting points for free energy simulations to study the catalytic mechanism (*vide infra*).

### Thermodynamic integration

Thermodynamic Integration and the blue moon ensemble method^74,75^ were used to compute the free-energy profiles for phosphodiester bond cleavage in the active state of HNH in the presence of Ca^2+^ and Co^2+^. Here, the average converged constraint forces are computed and integrated along a given RC, deriving the associated free energy profile. The difference in distance between the breaking and forming P–O bonds was used as RC, in line with studies of DNA cleavage by HNH in the presence of Mg^2+^^39^^,^. Starting from a RC = −1.5 Å, corresponding to the reactant state, a total of 26 sequential windows were sampled along the RC at 0.1 Å intervals, extending to the product state (RC = 1.5 Å). Each window was simulated for ≥10 ps, ensuring convergence of the constraint force (Supplementary Fig. 34), and resulting in a total of ~260 ps of ab initio MD. To estimate the error associated to hysteresis, we also computed the backward free energy profiles. These simulations were initiated from the post-transition state region (RC = 0.5 Å) and involved sampling 8 windows per system in the reverse direction along the RC. These simulations resulted in additional ~80 ps of sampling for each system. The statistical error at each point along the forward and backward free energy profiles was estimated using error propagation analysis. The total uncertainty in the free energy barrier was then obtained by combining the statistical error with the hysteresis error, calculated as the discrepancy between the forward and backward pathways. Details are in the Supplementary Methods.

### Metadynamics

To further elucidate the catalytic mechanism in the presence of Ca^2+^, QM/MM metadynamics^76^ simulations were performed on the Ca^2+^-bound active HNH complex. Here, a time-dependent biasing potential is added to the system’s Hamiltonian, applied as a function of selected collective variables (CVs) that represent the process of interest. Two CVs were employed: (i) the difference in distances between the breaking and forming P–O bonds, and (ii) the distance between the δ-nitrogen of H840 and the hydrogen atom of the nucleophilic water molecule. An extended Lagrangian formulation of the method was employed to ensure consistent coupling with the QM/MM framework^77^. For both collective variables, the fictitious particle mass was set to 16 amu, and the associated force constant was 0.24 au. The height of the Gaussian terms was 0.5 kcal/mol, while their width was set at 0.05 Å, consistent with the oscillations of the CVs observed in unbiased Car-Parrinello QM/MM simulation. A total of ~5000 Gaussians were deposited during the simulation, yielding a total of ~120 ps of metadynamics sampling. Multiple transitions between reactant and product states were observed (Supplementary Fig. 36), confirming thorough sampling. Free energy profiles were computed by averaging over ~10 ps blocks; errors were estimated using block averaging from the converged region. The statistical error associated with the free energy for phosphodiester bond cleavage was estimated using average blocking over ~10 ps segments from the converged portion of the metadynamics trajectory. Details are reported in the Supplementary Methods.

In vitro Cleavage. Mutants M1 (D54A/S55A/E57A), M2 (Q920A/E923A), and M3 (T13A/N14A) were generated by site-directed mutagenesis of the SpCas9 plasmid (Addgene pMJ806) using primers listed in Supplementary Table 2. Recombinant wild-type and mutant Cas9 proteins were expressed in *E. coli* BL21(DE3) cells, induced with 0.5 mM IPTG for 18 h at 18 °C, and purified by Ni-affinity chromatography followed by TEV tag cleavage and SP cation exchange chromatography. Proteins were concentrated, aliquoted, flash-frozen, and stored at −80 °C. Cas9 was pre-incubated with gRNA in 1.5X excess in cleavage buffer [20 mM Tris-HCl pH 7.5, 100 mM KCl, 5% glycerol, 1 mM DTT, 10 mM MgCl_2_] for 10 min at room temperature. Plasmid cleavage reactions were initiated at 37 °C combining 100 nM Cas9-gRNA with 2 nM circular plasmid containing an NGG PAM and Lambda1 target sequence. Aliquots were quenched at defined time points in 0.5 M EDTA and resolved on 1% agarose gels. Band intensities for supercoiled (SC), nicked (Ni), and linear (Li) species were quantified in FIJI and fit by non-linear regression using KinTek software: SC to a single exponential, Ni and Li to a double exponential. To generate a DNA substrate containing the NGG PAM and Lambda1 target sequence, fluorescently labelled oligos (5′-FAM label on TS or NTS) were annealed with their complement. Cas9–gRNA complexes were assembled as above and oligoduplex cleavage reactions initiated with 10 nM duplex at 37 °C. Aliquots were quenched in EDTA and analysed by capillary electrophoresis (ABI 3130xl) to quantify fraction cleaved over time. Data were fit to a single exponential using GraphPad Prism to determine *k*_obs_ for TS and NTS cleavage. The gRNA sequence used in cleavage experiments was 5′-GACGCAUAAAGAUGAGACGC + 80-mer SpCas9 scaffold-3′. The dsDNA substrates were prepared from complementary TS (5′-AGCTGACGTTTGTACTCCAGCGTCTCATCTTTATGCGTCAGCAGAGATTTCTGCT-3′) and NTS (5′-AGCAGAAATCTCTGCTGACGCATAAAGATGAGACGCTGGAGTACAAACGTCAGCT-3′) oligonucleotides. For fluorescence detection, either the target strand or the non-target strand carried a 5′-FAM label, depending on whether target strand or non-target strand cleavage was being resolved.

### NMR spectroscopy

The HNH-L2 domain (residues 772–925) of *S. pyogenes* Cas9 was cloned into pET-28a(+) with an N-terminal His₆-tag and TEV cleavage site. Protein was expressed in M9 minimal medium supplemented with ^15^NH_4_Cl and ^13^C-glucose, induced with 1 mM IPTG, and purified using Ni-NTA affinity and size-exclusion chromatography (full details in the Supplementary Information). Post-purification, protein was dialyzed into buffers containing KCl, MgCl_2_, or CaCl_2_ depending on the sample condition. NMR experiments were conducted at 600 MHz and 25 °C using a Bruker Avance NEO spectrometer. Backbone assignments were obtained using standard triple-resonance experiments (full details in Supplementary Methods)^78^ and validated against BMRB entry 27949^79^. Chemical shift perturbations were computed from ^1^H–^15^N HSQC spectra^80^ and parameters from *T*_1_, *T*_2_, ^1^H-[^15^N]-NOE, and CPMG relaxation dispersion experiments were measured using established protocols^81,82^ with uncertainties were derived from replicate spectra^35,36,83^. Histidine p*K*_a_ values were determined from ^1^H–^13^C HMQC titrations and fit using a modified Henderson–Hasselbalch equation^39,84^.

### Reporting summary

Further information on research design is available in the Nature Portfolio Reporting Summary linked to this article.

## Supplementary information

Supplementary Information

Reporting Summary

Transparent Peer Review file

## Source data

Source Data

## Data Availability

Atomic coordinates of the optimized computational models are available in Figshare at https://doi.org/10.6084/m9.figshare.29114687. NMR resonance assignments for the HNH nuclease are available in the Biological Magnetic Resonance Data Bank (BMRB), accession 27949. Additional supporting data are provided in the Supplementary Information. Source data are provided with this paper.
