# Supplementary material for: A cryptic binding pocket regulates the metal-dependent activity of Cas9
Source: Nat Commun. 2026 Oct 9;17:11501. doi: 10.1038/s41467-026-78458-0 (PMC13745514; doi:10.1038/s41467-026-78458-0)
Supplement: Supplementary file 1 [file 41467_2026_78458_MOESM1_ESM.pdf]

Supplementary Information

A Cryptic Binding Pocket Regulates the Metal-Dependent Activity of Cas9

Mohd Ahsan, Alexa L. Knight, Aakash Saha, Delisa Ramos, Isabel Strohkendl, Erin Skeens, Madeline S. West, Jinping Luo, Charlotte S. Dresser, David W. Taylor, George P. Lisi, and Giulia Palermo

Table of Contents

S1. Supplementary Materials and Methods ... 2

S1.1. Structural models ... 2

S1.2. Classical Molecular Dynamics (MD) simulations ... 2

S1.3. MD simulations of additional monovalent, divalent, and trivalent cations ... 3

S1.4. Analysis of ion-dependent conformational responses ... 4

S1.5. Umbrella Sampling Simulations ... 5

S1.6. Markov State Models (MSM) ... 6

S1.7. Markov State Models (MSM) analysis of Divalent Binding Pocket (DBP) lifetime ... 8

S1.8. Quantum Mechanics/Molecular Mechanics (QM/MM) simulations ... 9

S1.9. Thermodynamic Integration ... 10

S1.10. Metadynamics ... 11

S1.11. *In vitro* Cleavage ... 12

S1.12. NMR Spectroscopy ... 14

S1.13. Differential Scanning Fluorimetry (DSF) ... 16

S2. Supplementary Results ... 17

S2.1. Ion-dependent modulation of HNH conformational activation ... 17

S3. Supplementary Figures ... 19

S4. Supplementary Tables ... 65

S5. Raw Data ... 67

S6. Supplementary References ... 69

S1. Supplementary Materials and Methods

S1.1. Structural models

Molecular simulations were conducted based on two experimental structures of the *Streptococcus pyogenes* Cas9 (SpCas9), representing the pre-active and active configurations of the catalytic HNH domain. The crystallographic structure PDB: 5F9R[1], solved at 3.40 Å resolution, depicts the pre-active state of the complex, with the HNH domain approximately 14 Å away from the scissile phosphate[1]. The cryo-EM structure EMD-0584 (PDB: 6O0Y[2]), solved at 3.37 Å resolution, illustrates the catalytically competent state of Cas9, where the HNH domain is aligned with the target DNA strand and positioned at the scissile phosphate[2]. Since this structure corresponds to the DNA post-cleavage state, the P–O3′ bond in the catalytic site was carefully reconstructed following energy minimization and equilibration at the classical molecular dynamics level (*vide infra*). Both the pre-active and active systems were solvated in explicit water molecules and neutralized using the divalent ion under study, *viz.,* Mg^{2+}, Ca^{2+}, or Co^{2+}. To achieve the desired ionic environment, 13 molecules of $MgCl_2$, $CaCl_2$, or $CoCl_2$ were added to each system to obtain a 10mM (high) concentration, while one molecule was added to achieve a 10μM (low) concentration. This setup resulted in periodic simulation cells of approximately 180 x 120 x 140 $Å^3$, containing a total of around 340,000 atoms for each system.

S1.2. Classical Molecular Dynamics (MD) simulations

Molecular dynamics (MD) simulations at the classical molecular mechanics level were conducted using a protocol tailored for protein/nucleic acid complexes[3], applied in studies of CRISPR-Cas systems[4–7]. The Amber ff19SB[8] force field was employed, incorporating the OL15[9] and OL3[10] corrections for DNA and RNA, respectively. The TIP3P[11] model was used for water molecules. The 12-6 Li & Merz model was used for Mg^{2+}, Ca^{2+} and Co^{2+} ions[12]. This model described the metal active site in agreement with quantum mechanics/molecular mechanics (QM/MM) simulation (Supplementary Fig. 31). The 12-6-4 polarizable Li-Merz[13,14] and the Allnér[15] parameters were also used to model Mg^{2+} ions in the pre-active state under high and low Mg^{2+} concentrations. The results showed consistent trends in divalent ion interactions across all three models (Supplementary Fig. 16). An integration time step of 2 fs was used, with hydrogen atoms added assuming standard bond lengths and constrained to their equilibrium positions using the SHAKE algorithm. Temperature control was maintained at 300 K via Langevin dynamics[16], with a collision frequency $\gamma = 1$. Pressure control was achieved by coupling the system to a Berendsen barostat[17] at a reference pressure of 1 atm and with a relaxation time of 2 ps. The systems underwent energy minimization to relax water molecules and counter ions, with the protein, RNA,

DNA, and Mg^{2+} ions held fixed using harmonic position restraints of 300 kcal/mol·Å^2. The systems were then gradually heated from 0 to 100 K in the canonical ensemble (NVT) by running two simulations of 5 ps each, with position restraints of 100 kcal/mol·Å^2 on the key components of the system. The temperature was further increased to 200 K over ~100 ps of MD in the isothermal-isobaric ensemble (NPT), during which the restraint was reduced to 25 kcal/mol·Å^2. Subsequently, all restraints were removed, and the system temperature was raised to 300 K in a single NPT simulation of 500 ps. After ~1.1 ns of equilibration, ~10 ns of NPT was carried out, allowing the system density to stabilize around 1.01 g/cm^3. Finally, production runs were carried out in the NVT ensemble, yielding an aggregate sampling time of multiple microseconds (μs). To assess ion-dependent conformational activation, MD simulations of the pre-active state were performed under high (10 mM) and low (10 μM) concentrations of Mg^{2+}, Ca^{2+}, and Co^{2+}. These conditions were chosen to reflect the ionic regimes used in single-molecule and biochemical studies of Cas9[18,19]. Additional simulations were carried out at an intermediate Mg^{2+} concentration (2 mM). Furthermore, the three mutants M1 (D54A/S55A/E57A), M2 (Q920A/E923A), and M3 (T13A/N14A) were simulated under high Mg^{2+} concentration conditions. Simulations of the active state were performed under high ionic concentration conditions, consistent with *in vitro* assays, to assess ion-dependent effects on HNH active-site organization. For each simulation system, three simulation replicates of ~1 μs each were conducted, totalling over ~39 μs of classical MD simulations. All classical MD simulations, including free energy simulations (*vide infra*), were performed using the GPU-accelerated version of AMBER 22[20].

S1.3. MD simulations of additional monovalent, divalent, and trivalent cations

To assess the generality of ion-dependent conformational activation, additional MD simulations were performed on the pre-active state of Cas9 (PDB: 5F9R[1]) in the presence of a broader set of metal ions, including monovalent (Li^+), divalent (Be^{2+}, Mn^{2+}, Zn^{2+}, Ra^{2+}), and trivalent (Al^{3+}) cations. For each ion, simulations were carried out under both high (10 mM) and low (10 μM) concentration conditions, using the same protocol and system setup as described above for Mg^{2+}, Ca^{2+}, and Co^{2+}. Metal ions were described using parameters developed by Merz and co-workers. Specifically, Li^+ was modelled using the monovalent ion parameters reported by Li et al.[21]; Be^{2+}, Mn^{2+}, Zn^{2+} and Ra^{2+} were modelled using the 12-6 parameters for divalent ions developed by the same authors[12], which were also employed for Mg^{2+}, Ca^{2+} and Co^{2+} (*vide supra*), and Al^{3+} was modelled using the parameters developed for highly charged ions[22]. Each system was simulated in three independent replicas of ~1 μs each, totalling additional ~36 μs of classical MD simulations.

S1.4. Analysis of ion-dependent conformational responses

To characterize ion-dependent conformational responses of the HNH domain, all simulation trajectories (including those with monovalent, divalent and trivalent cations) were analysed using two structural observables: (i) the distance between the catalytic residue H840 (Cα atom) and the scissile phosphate (P_{SCI}), denoted $d_{H840-PSCI}$, and (ii) the distance between the center of mass of the HNH domain and P_{SCI}, denoted $d_{HNH-PSCI}$ (Supplementary Fig. 13a). These coordinates report on local catalytic positioning and global domain movement, respectively. For each ion and concentration condition, three independent replicas were analysed. Replica-averaged mean distances were computed separately for high- and low-concentration ensembles, and concentration-dependent changes were defined as:

$$\Delta d_{H840-PSCI} = d_{H840-PSCI}^{LOW} - d_{H840-PSCI}^{HIGH}, \quad (1)$$

$$\Delta d_{HNH-PSCI} = d_{HNH-PSCI}^{LOW} - d_{HNH-PSCI}^{HIGH}, \quad (2)$$

such that positive values indicate reduced distances under high-concentration conditions, corresponding to closer approach of the catalytic residue or the HNH domain to P_{SCI} (Supplementary Fig. 13b-c).

To characterize sampling of configurations closer to P_{SCI}, operational thresholds were defined independently for $d_{H840-PSCI}$ and $d_{HNH-PSCI}$ using the high-concentration ensemble as a reference. Specifically, thresholds $d_{H840-PSCI}^{*}$ and $d_{HNH-PSCI}^{*}$ were defined as the 25th percentile of the corresponding distributions pooled across the three high-concentration replicas for each ion. These thresholds identify the subset of configurations corresponding to the lower tail of the distance distributions (i.e., closest to P_{SCI}) sampled under high-concentration conditions and provide a consistent, ion-specific reference for comparing different ionic environments.

Because conformational activation requires both catalytic positioning and domain alignment, a joint metric was used to quantify simultaneous sampling of these configurations. Specifically, a frame was classified as satisfying a combined criterion when both conditions were met: $d_{H840-PSCI} < d_{H840-PSCI}^{*}$ and $d_{HNH-PSCI} < d_{HNH-PSCI}^{*}$. For each trajectory, the fraction of frames satisfying both conditions was computed, yielding an activation probability P. Replica-averaged values were obtained for both high- and low-concentration ensembles, and concentration dependence was quantified as:

$$\Delta P = P_{HIGH} - P_{LOW}, \quad (3)$$

where positive values indicate increased sampling of configurations in which both the catalytic residue and the HNH domain approach P_{SCI} under high-concentration conditions, whereas

negative ΔP values indicate that such configurations are more frequently sampled under low-concentration conditions (Supplementary Fig. 13d).

For each ion and concentration condition, reported quantities were averaged over three independent replicas. The standard error of the mean (s.e.m.) was calculated across the three replicas for each concentration independently. For concentration-dependent differences (Δd and ΔP), the difference between the independently averaged high- and low-concentration ensembles was calculated as defined above, and the associated uncertainty was obtained by propagation of the corresponding standard errors.

S1.5. Umbrella Sampling Simulations

The umbrella sampling (US) method[23] was employed to compute the free energy profile associated with the conformational change of the HNH domain from the pre-active (PDB 5F9R)[1] to the active (PDB 6O0Y)[2] conformation. In this approach, multiple simulations (US windows) are run in parallel, with an additional harmonic bias potential applied to selected Reaction Coordinates (RCs):

$$V(RC) = \frac{k}{2}(RC(t) - RC^*)^2 \tag{4}$$

where $V(RC)$ is the value of the bias potential, k is a bias force constant, $RC(t)$ is the value of RC at given time t and RC^* is the reference value of RC. By using different RC^* values in each US window, one can sample the biased probability distribution $p_b(RC)$ along the whole RC range of interest. Thanks to a simple reweighting scheme (*vide infra*) providing the unbiased probabilities, the free energy of a process along the RCs can be computed.

The HNH domain differs in conformation and position with respect to the recognition lobe (REC) when comparing the pre-activated and active states (Supplementary Fig. 1). Therefore, the difference in root-mean-square deviation (RMSD) of the Cα atoms in HNH and portions of REC (residues 175-393, 778-906) relative to the pre-activated and active states, was used as a RC (Supplementary Fig. 2a). Restraint forces were applied on the RC as follows. The RMSD between initial and final conformations was reduced, according to the following potential:

$$V = \begin{cases} \frac{k}{2N}\left(RMS(t) - RMS^*(t)\right)^2, & RMS(t) > RMS^*(t) \\ 0, & RMS(t) \le RMS^*(t) \end{cases} \tag{5}$$

where k is the collective force constant, N is the number of targeted atoms, $RMS(t)$ is the actual RMSD at initial time t, and $RMS^*(t)$ is gradually reduced from the initial RMSD value (~7.4 Å) between the initial and target configurations toward the desired final RMSD (i.e., zero). The collective force constant k was progressively increased from 20 kcal/mol·Å² at the beginning of

the simulation to 100 kcal/mol·Å^2. Two independent sets of US simulations were performed: (i) under high concentration of Mg^{2+} (10 mM) and (ii) under low concentration of Mg^{2+} (10 μm). In both cases, the system was simulated in 36 overlapping windows, with the RC ranging from 7.4 Å to 0.2 Å, using a harmonic restraint with a spring constant of 50 kcal/mol·Å^2. The centre of the harmonic bias potential was distributed along the RC in 36 windows, each separated by 0.2 Å, ensuring proper overlap of the probability distributions. For each system, ~120 ns-long trajectories were obtained per US window, resulting in ~4.3 μs of collective sampling per system (and a total of ~8.6 μs). The free energy profiles were then computed through energetic reweighting using the Weighted Histogram Analysis Method (WHAM)[24]. To ensure the accuracy of the free energy profiles, we evaluated the convergence by excluding portions of the US windows corresponding to the initial relaxation of the system. Specifically, we removed the first ~20 ns of the US trajectories from each window and computed the free energy profiles in steps of 10 ns, using the remaining ~100 ns of data per window (Supplementary Fig. 2d). Convergence was reached ~70 onboard ns for both systems. Error estimation on the free energy profile was performed using Monte Carlo bootstrap error analysis[25].

S1.6. Markov State Models (MSM)

The Markov State Model (MSM) is a kinetic model that simulates continuous dynamics as a series of Markov jumps between discrete states at specific time intervals, enabling the systematic prediction of long-time scale dynamics from a large set of short, unbiased MD trajectories[26,27]. To characterize the kinetics of the HNH transition from the pre-activated to the active state of CRISPR-Cas9, MSMs were developed using unbiased MD simulations at high (10 nm) and low (10 μs) concentrations of Mg^{2+} ions. To construct MSMs along the HNH conformational change, representative structures along the transition were extracted from US simulations, which effectively described the conformational change of HNH and provided insights into its thermodynamics (details in the main text, Fig. 1 and Supplementary Fig. 2). In detail, the conformations from each of the 36 US window, each approximatively 100 ns in duration (see section 1.3), were clustered to generate a total of 36 structures. These structures were subsequently utilized for initiating the MSM simulations, with one representative structure selected from each US window. These seeding structures were solvated and simulated for additional unbiased runs, collecting ~100 ns for each structure, following the simulation protocol described above (see section 1.2 of this Supplementary Information). In total, 36 trajectories were accumulated, resulting in approximately 3.6 μs of simulation time for each system, in the presence of high and low concentration of Mg^{2+}. The simulation trajectories were imported into the PyEMMA

package for MSM analysis[28]. Two inter-residue distances were selected as features (i.e., independent components, IC): the distance between the Cα atoms of residues S355 and S867 (IC1), and the distance between the Cα atoms of residues S867 and N1054 (IC). These distances were used in Förster Resonance Energy Transfer (FRET) experiments to describe the conformational dynamics of the HNH transition from the inactive to the active state[18,29]. The evolution of these distances over the total simulation time for each system (at high and low concentration of Mg^{2+}) is shown in Supplementary Fig. 4. Using these two features (IC1 and IC2), a time-lagged independent component analysis (TICA)[30] was performed, with a lag time of 20 steps (equivalent to 10 ns, given the step size of 0.25 ns). The trajectories were then discretized into 5 to 200 microstates using the k-means clustering method[31,32], and the clusters were compared by calculating the variational principle of conformational dynamics, which provides a scalar value (VAMP-2 score[33], Supplementary Fig. 5a). A cluster size of 100 microstates was found to saturate the VAMP-2 score for both systems. Consequently, k-means clustering was performed on the TICA space, selecting 100 clusters (Supplementary Fig. 5b). To determine the appropriate lag time for the final MSM, the implied timescales (ITS)[34] were plotted as a function of lag time (Supplementary Fig. 5c). In MSM, the smallest lag time where ITS converge is selected as an indicator of the Markovian nature of the model. For both systems, the ITS converged at ~10 ns, which was used to build the final MSM. Bayesian Markov state models[35,36] were then created, selecting a lag time of 5 ns for each MSM. To ensure that the constructed MSM was genuinely Markovian, the Chapman–Kolmogorov (CK) test[37] was also performed (Supplementary Fig. 5d). This analysis showed that the estimated and predicted lines overlap up to ~15 ns (3x the MSM lag time) with a 95% confidence interval, confirming that these MSMs are statistically valid for four macrostates.

Perron Cluster Cluster Analysis (PCCA++)[38] was then applied to cluster the first two eigenvectors of the MSM transition matrices, enabling the coarse graining of the 100 microstate transition matrix into four macrostates. This fuzzy clustering algorithm calculates the probability of each microstate belonging to each macrostate. The maximum assignment probability was used to assign 100 microstates into the four macrostates. Visualizations of the macrostate assignments within the TICA space are provided in Supplementary Figs. 6 and 7, corresponding to the systems at high and low concentration of Mg^{2+}, respectively. The stationary probability of each macrostate is shown in Supplementary Fig. 8 for both systems. The stationary distribution method in PyEMMA was utilized to recompute the statistical weight of the MD configurations obtained from the trajectories. The stationary distribution represents the long-term probability distribution over the states in the MSM model, indicating the likelihood of the system occupying each state at

equilibrium. The free energy landscapes were obtained by reweighting trajectory frames using the stationary probabilities derived from the MSM[26] (Fig. 2c, main text). Specifically, trajectory frames were grouped according to their MSM state assignments, using the stationary probabilities derived from the MSM. Each state's equilibrium probability was evenly distributed among its associated frames, and these per-frame weights were used to reconstruct the reweighted free energy surfaces. Finally, Transition Path Theory (TPT)[39,40] was applied to compute the fluxes from a source state (A, pre-activated) to a sink state (B, active), providing insights into the structural mechanism and kinetics of the A -> B transition. The net fluxes were computed to define a directed graph from A to B states based on the backward and forward committor probabilities.

S1.7. Markov State Models (MSM) analysis of Divalent Binding Pocket (DBP) lifetime

The lifetime of the cryptic pocket was estimated from the MSM by treating the pocket as a dynamic structural ensemble rather than a static metal-binding site. Its persistence was quantified from the structural integrity of the residues lining the pocket. The cryptic pocket was defined by residues T13, N14, D54, S55, and E57 on the RuvC side and residues Q920 and E923 of the L2 domain. To monitor the structural persistence of the pocket, pairwise distances were calculated between residues T13, N14, D54, S55, and E57 and residues Q920 and E923, giving ten pocket-defining distance pairs. A reference pocket geometry was taken from the initial pocket-containing structure, and each distance pair was considered preserved at frame t if its value remained within a tolerance of the corresponding reference distance:

$$d_{ij}(t) \leq \; d_{ij}^{ref} + 2.0\ \text{Å} \tag{6}$$

where $d_{ij}(t)$ is the distance between pocket-lining residues i and j at frame t, and d_{ij}^{ref} is the corresponding reference distance in cryo-EM structure (PDB: 5F9R)[1]. The pocket order parameter was then defined as:

$$Q_{pocket}(t) = \frac{1}{N} \sum_{ij=1}^{N} I \,[\, d_{ij}(t) \leq \; d_{ij}^{ref} + 2.0\ \text{Å}] \tag{7}$$

where $N = 10$ is the total number of pocket-defining distance pairs and I is an indicator function equal to 1 when a given distance pair is preserved and 0 otherwise. Thus, Q_{pocket} represents the fraction of native pocket-defining distances retained at each frame. To avoid assigning a pocket as intact solely on the basis of local pairwise distances, the pocket root-mean-square deviation (RMSD) was also used as a geometric filter. A frame was classified as pocket-intact when:

$$Q_{pocket}(t) \;\geq 0.5 \tag{8}$$

and:

$$RMSD_{pocket}(t) \leq 2.0\text{Å}. \tag{9}$$

Frames not satisfying both criteria were classified as pocket-disrupted. This binary pocket-state assignment was then projected onto the MSM metastable states. For each metastable state s, the probability of retaining an intact pocket was calculated as:

$$P_s(pocket\ intact) = \frac{N_s(pocket-intact\ frames)}{N_s(total\ frames)} \tag{10}$$

where N_s is the number of frames assigned to metastable state s. In this analysis, the MSM contained four metastable states (Fig. 2 and Supplementary Fig. 6). States A and 1 retained substantial pocket-like character and were therefore grouped as the pocket-containing ensemble, whereas states 2 and B showed reduced pocket-intact probability and were grouped as the pocket-disrupted ensemble.

The kinetic lifetime of the cryptic pocket was then estimated as the mean first passage time (MFPT) from the pocket-containing ensemble to the disrupted-pocket ensemble:

$$\tau_{pocket} = MFPT_{[\mathrm{A},1]\rightarrow[2,\mathrm{B}]} \tag{11}$$

This quantity reports the ensemble-level persistence of the cryptic-pocket architecture before transition to the disrupted-pocket ensemble. The MFPT was calculated from the MSM using PyEMMA and converted to physical time using the trajectory time resolution.

Uncertainty in the MSM-derived pocket lifetime was estimated by block bootstrapping the metastable-state trajectory. Contiguous trajectory blocks were resampled with replacement to generate bootstrap state trajectories. For each bootstrap replicate, a transition matrix was re-estimated at the same MSM lag time, and the MFPT from the pocket-containing ensemble to the disrupted-pocket ensemble was recalculated. The resulting bootstrap distribution was used to estimate the mean, median, standard deviation, standard error, and 95% confidence interval of the cryptic-pocket lifetime.

S1.8. Quantum Mechanics/Molecular Mechanics (QM/MM) simulations

QM/MM simulations were conducted on the active conformation of the HNH domain in the presence of Ca^{2+} and Co^{2+} in the active site. The QM region included the divalent ion, its coordinating residues (D839, N863), the catalytic H840, DNA nucleobases G-3, T-4, and C-5, and nine water molecules (including the nucleophile, two ion-coordinating waters, and six surrounding waters). Capping hydrogen atoms were used to saturate the valence of the terminal QM part, resulting in a total of 123 QM atoms. The catalytic H840 was modelled in its neutral tautomeric

form, protonated at the ε position, as indicated by Nierzwicki et al., who previously conducted QM/MM simulations in the presence of Mg^{2+} ions[41].

The QM atoms were described at the QM DFT/BLYP[42,43] level, while the remaining MM region was treated using the force field described above. QM/MM simulations were performed using the CPMD 4 code[44]. The wave functions were expanded in a plane wave basis set with a cutoff of 75 Ry in a QM cell with dimensions of approximately 26 x 28 x 26 $Å^3$. Interactions between the valence electrons and ionic cores were described using norm-conserving Martins-Troullier pseudopotentials[45]. The QM part was treated as an isolated system, with electrostatic interactions between periodic images decoupled using Tuckerman's scheme[46]. A rigorous Hamiltonian treatment of the electrostatic interaction between the QM and MM regions was applied[47]. The QM/MM protocol began with an initial optimization of the wavefunction, followed by ~6 ps of equilibration using Born–Oppenheimer MD in the canonical (NVT) ensemble with an integration time step of 20 au (~0.48 fs). The temperatures of the QM and MM subsystems were maintained at 300 K using a Nosé-Hoover thermostat. After this equilibration phase, Car-Parrinello QM/MM simulations were carried out with a time step of 5 au (~0.12 fs) and a fictitious electron mass of 600 au. To assess the conformation of the active site in the presence of Ca^{2+} and Co^{2+}, ~20 ps of unconstrained ab-initio MD were performed in two replicates for each system. The results were compared to the Mg^{2+}-bound configuration, previously simulated by Nierzwicki et al. using the same methodological approach applied in this study[41]. The active site conformations derived from these ~20 ps unconstrained ab-initio MD simulations were subsequently used as starting points for free energy simulations to study the catalytic mechanism.

S1.9. Thermodynamic Integration

The catalytic mechanism of phosphodiester bond cleavage at the HNH catalytic site was characterized using a Thermodynamic Integration approach combined with the “blue moon ensemble” method[48,49]. This study considered the HNH active conformation in the presence of Ca^{2+} and Co^{2+} ions. In this approach, the average converged constraint forces are computed at each step along a given Reaction Coordinate (RC) and subsequently integrated to derive the associated free energy profile (Supplementary Fig. 33). The difference in distance between the breaking ($O3'_{DNA}$–P_{SCI}) and forming (O_{WAT}–P_{SCI}) bonds was used as the RC (shown in Fig. 5, main text). This approach has proven suitable for describing phosphodiester bond cleavage in studies of DNA/RNA-cleaving enzymes[50,51], including recent work on the HNH catalysis in Cas9 in the presence of Mg^{2+}[41]. Starting with an RC value of -1.5 Å, we sampled 26 sequential windows along the RC, with a resolution of 0.1 Å. A growth rate of 0.1 Å/ps was used to transition from one

window to the next. Each window was simulated for ~10 ps, allowing the constraint force to reach convergence (Supplementary Fig. 34), resulting in extensive and well-converged simulations, collecting a total of ~260 ps of ab-initio QM/MM MD for each system.

To estimate the error associated with hysteresis, we also computed the backward pathways for each free energy profile (Supplementary Fig. 33). For the system including Ca^{2+} in the active site, we selected the point at RC = 0.5 Å (after the transition state was reached, towards product formation) and then followed the backward pathway at a growth rate of 0.1 Å/ps, sampling eight consecutive points at RC values of 0.5 Å, 0.4 Å, 0.3 Å, 0.2 Å, 0.1 Å, 0.0 Å, -0.1 Å, and -0.2 Å. Similarly, for the system including Co^{2+} in the active site, the backward pathway was calculated at RC values of 0.1 Å, 0.0 Å, -0.1 Å, -0.2 Å, -0.3 Å, -0.4 Å, -0.5 Å, and -0.6 Å. Each window was simulated for ~10 ps, resulting in an additional ~80 ps per system. In the presence of Ca^{2+}, the free energy barriers for the forward and backward pathways were ~18.4 ± 1.03 kcal/mol and ~20.25 ± 0.90 kcal/mol, respectively (Supplementary Fig. 33a). The difference between the forward and backward pathways was ~1.85 kcal/mol, leading to a total Helmholtz free energy for the reaction of ~18.4 ± 1.85 kcal/mol. For the system with Co^{2+}, the free energy barriers were ~27.6 ± 1.33 kcal/mol (forward) and ~30.03 ± 1.20 kcal/mol (backward), with a difference of ~2.43 kcal/mol, resulting in a total Helmholtz free energy for the reaction of ~27.6 ± 2.43 kcal/mol. The free energy profiles (both forward and backward) were derived by considering the average constraint force within each window from the points where the force reached convergence along the equilibrated trajectory (i.e., upon discarding the first ~5 ps in each window). The statistical error at each point of the free energy profiles was computed using error propagation analysis. The overall error on the free energy barrier was estimated by summing the statistical error and the hysteresis error between the forward and backward pathways. Altogether, considering the forward and backward pathways, as well as the unconstrained runs, we collected ~340 ps of ab-initio QM/MM MD for each system.

S1.10. Metadynamics

To further characterize the catalytic mechanism of phosphodiester bond cleavage in the presence of Ca^{2+} in the HNH active site of CRISPR-Cas9, we performed QM/MM MD simulations combined with metadynamics[52]. The use of metadynamics allowed us to explicitly account for the degrees of freedom involved in phosphodiester bond cleavage and the deprotonation of the water nucleophile. This method enables the dynamic exploration of the free energy surface across multiple dimensions by applying an external, history-dependent bias potential to the system's Hamiltonian, based on a set of predefined degrees of freedom (collective variables, CVs). We

selected two CVs for this study: (i) the difference in distance between the breaking ($O3'_{DNA}$–P_{SCI}) and forming (O_{WAT}–P_{SCI}) P–O bonds, and (ii) the distance between the H840 δ nitrogen and the hydrogen of the water nucleophile (Supplementary Fig. 36). This approach allowed us to map the free energy associated with phosphodiester bond cleavage along one dimension (CV1), while simultaneously characterizing nucleophile activation along another (CV2).

An extended Lagrangian version of metadynamics was used to ensure proper coupling with the QM/MM simulations[53]. For both CVs, the selected mass value of the fictitious particles was 16 amu, and the force constant was set to 0.24 au. The height of the Gaussian terms was 0.5 kcal/mol, providing sufficient accuracy for reconstructing the free energy surface, while the width of the Gaussian terms was set at 0.05 Å, consistent with the oscillations of the CVs observed in a free Car-Parrinello QM/MM simulation. Gaussian functions were deposited at a rate of 24 fs, with a total of ~5000 Gaussians deposited during the simulation, yielding a total of ~120 ps of metadynamics sampling. During this simulation time, the chemical step transitioned from the reactants to products multiple times (Supplementary Fig. 36), allowing for comprehensive sampling of the conformational space. The statistical error associated with the free energy for phosphodiester bond cleavage was estimated using average blocking, which involves computing and averaging the standard deviation of the mean free energy over ~10 ps blocks from the converged portion of the metadynamics simulation

S1.11. *In vitro* Cleavage

Recombinant constructs of spCas9 harbouring mutations at each magnesium binding pocket (M1: D54A, S55A, E57A, M2: Q920A, E923A, M3: T13A, N14A), were made by performing site-directed mutagenesis on the spCas9 plasmid (Addgene: pMJ806) using the DNA primers listed in Supplementary Table 2. BL21(DE3)-competent cells (Invitrogen) containing recombinant plasmids with either wild-type SpCas9 or each Cas9 mutation cluster were cultured in LB medium at 37 °C until the optical density at 600 nm (OD600) reached 0.6. Protein expression was induced by adding a final concentration of 0.5 mM isopropyl-β-d-1-thiogalactopyranoside (IPTG) for 18 h at 18 °C. The bacterial pellets were lysed via sonication in lysis buffer [20 mM Tris-HCl pH 7.5, 10% glycerol, 1 M NaCl, 0.5 mM tris(2-carboxyethyl) phosphine (TCEP), 10 mM MgCl2, 1X DNase, 2 mM phenylmethylsulfonyl fluoride (PMSF) and Pearce protease inhibitor cocktails (Thermo Scientific)]. Clarified lysate was loaded onto a HisTrapTM HP column, washed with wash buffer [20 mM Tris-HCl pH 7.5, 10% glycerol, 1 M NaCl, 0.5 mM TCEP] and eluted in elution buffer [20 mM Tris-HCl pH 7.5, 10% glycerol, 1 M NaCl, 250 mM imidazole (pH 8.0) and 0.5 mM TCEP]. Fractions containing the protein of interest were added to dialysis tubing and incubated in dialysis

buffer [20 mM HEPES pH 7.5, 10% glycerol, 150 mM KCl and 0.5 mM TCEP] at 4 °C for 16 h. TEV protease was added for affinity tag cleavage during dialysis. Dialysed protein was then loaded onto a HiTrap SP HP cation exchange chromatography column, washed in an ion exchange wash buffer [20 mM HEPES pH 7.5, 10% glycerol, 150 mM KCl and 0.5 mM TCEP], and eluted in an elution buffer [20 mM HEPES pH 7.5, 10% glycerol, 1 M KCl and 0.5 mM TCEP]. Eluted SP column protein fractions were pooled, concentrated, aliquoted, flash-frozen in liquid nitrogen, and stored at −80 °C.

To perform plasmid cleavage assays 100nM of purified Cas9 protein was incubated with 150nM lambda1 gRNA in cleavage buffer [20mM Tris-HCl pH 7.5, 100mM KCl, 5% Glycerol, 1mM DTT, and 10mM $MgCl_2$] for 10 minutes at room temperature. Cas9 cleavage was initiated at 37° C with the addition of a 2nM circular plasmid containing an NGG PAM and Lambda1 target sequence. At time points [5, 15, 30, 45, 60, and 150 seconds], 1µl of reaction was quenched in 2µl of 0.5M EDTA. Undigested plasmid DNA represented the 0 second time point. Quenched samples were visualized on a 1% agarose gel stained with SybrSafe and visualized using a Bio-Rad ChemiDoc MP imager. Each reaction was performed in triplicate. For each time point, relative amounts of each plasmid species were calculated via densitometry analysis in FIJI and analyzed by non-linear regression using KinTek modelling software. Loss of supercoiled substrate was fit to a single exponential curve [$y = a * (1 - e^{-k*x})$] in which 'a' represents the amount of SC substrate at t=0. The rate of decay '*k*' was then used as a fixed value for the rate of appearance of the Ni species or to represent the apparent lag in the appearance of the Li product species. Ni and Li species were fit to a double exponential curve [$y = a1 * (1 - e^{-k1*x}) + a2 * (1 - e^{-k2*x}) + a0$]. The rate of Ni species disappearance or Li product appearance (*k2*) are the same values, but the equations differ in the relative amplitudes.

To perform oligoduplex cleavage assays[54], oligos were designed with an NGG PAM and Lambda1 target sequence and a 5′-FAM fluorescent label was added to either the target or non-target strand. These duplexes were formed by annealing complementary oligos at 4µM in annealing buffer [10mM Tris-HCl pH 7.5, 50mM NaCl, 1mM EDTA] by heating to 95° C and slow cooling at room temperature. Purified Cas9 cluster mutants were assembled with gRNA as described above. Cas9 cleavage was initiated at 37 °C by adding 10 nM of the FAM-labelled DNA duplex (labelled on either the target or non-target strand). At the designated time points, 1µl of reaction was quenched in 2µl of 0.5M EDTA. Each reaction was performed in triplicate. Target and non-target strand cleavage events were resolved through capillary electrophoresis using an Applied Biosystems DNA sequencer (ABI 3130xl) to determine fraction cleaved over time. These

time courses were fit to a single exponential curve (shown above) by non-linear regression using GraphPad Prism.

To perform Li^{+}, Mg^{2+}, and Al^{3+} metal ion cleavage assays, *Streptococcus pyogenes* Cas9 ribonucleoprotein (RNP) complexes were assembled by combining Cas9 protein with crRNA and tracrRNA at a molar ratio of 1:1.2:1 (Cas9:crRNA:tracrRNA). Cas9 protein was prepared from a 12 μM stock solution, while crRNA (IDT 26002-g1 crRNA; 55 μM stock) and tracrRNA (IDT Alt-R CRISPR-Cas9 tracrRNA; 89.4 μM stock) were diluted in an annealing buffer of 10 mM Tris-HCl and 100 mM KCl at pH 7.5. Guide RNAs were annealed by mixing equimolar concentrations of crRNA and tracrRNA (10 μM final concentration each), heating the mixture to 95 °C for 5 min, and gradually cooling to room temperature over 30 min. RNP complexes were subsequently assembled to a final concentration of 1 μM and incubated at room temperature for 10 min prior to use in cleavage assays. Double-stranded DNA substrates consisted of a 483 bp linear PCR amplicon containing the target protospacer and PAM sequence. DNA substrates were generated using DreamTaq Green PCR Master Mix and mouse genomic DNA template under the following cycling conditions: 35 amplification cycles with a 60 °C annealing step and a 45 s extension step. Purified DNA substrates were used at a final concentration of 50 ng/μL (16.8 nM). Cleavage reactions were performed in a cleavage buffer of 20 mM Tris-HCl, 100 mM KCl, 5% (v/v) glycerol, and 1 mM DTT at pH 7.5. Reactions were supplemented with the indicated metal ion species (Mg^{2+}, Li^{+}, or Al^{3+}) at final concentrations between 0 – 10,000 nM. Cas9 RNP complexes were added to a final concentration of 500 nM in a total reaction volume of 10 μL. Reaction mixtures were assembled on ice, incubated at 37 °C for 30 min, and terminated by heating to 65 °C for 10 min. Cleavage products were analyzed by electrophoresis on 1.5% agarose gels stained with GelRed nucleic acid stain and visualized by UV transillumination. For gel loading, 6 μL of reaction product was mixed with 2 μL of 6x DNA loading dye prior to electrophoresis. No-RNP control reactions were included for each experimental condition. All cleavage assays were performed in triplicate. Cleavage buffer reagents were prepared from concentrated stock solutions consisting of 4x base buffer (80 mM Tris-HCl, 400 mM KCl, and 20% glycerol at pH 7.5), 8x metal ion stocks, and freshly prepared 8x DTT solution derived from a 1 M stock.

S1.12. NMR Spectroscopy

The HNH-L2 domain (residues 772-925) of *S. pyogenes* Cas9 was engineered into a pET-28a(+) vector including an N-terminal His_6-tag and a TEV protease cleavage site. The plasmid was transformed into BL21-Gold (DE3) competent cells (Agilent) and expressed in M9 minimal media isotopically enriched with $^{15}NH_4Cl$ and $^{13}C_6H_{12}O_6$ (Cambridge Isotope Laboratories) as the

sole nitrogen and carbon sources and supplemented with $MgSO_4$, $CaCl_2$, and MEM vitamins. Cells were grown at 37°C to an OD_{600} of 0.8 – 1.0, induced with 1.0 mM IPTG, incubated for 16-18 hours at 20°C, and subsequently harvested by centrifugation.

For purification of HNH-L2, cells were resuspended in a buffer of 20 mM HEPES, 300 mM potassium chloride, and 10 mM imidazole at pH 8.0 with an EDTA-free protease inhibitor cocktail tablet (Sigma Aldrich) and lysed by sonication. Following centrifugation, the supernatant was incubated with Ni-NTA resin, washed with lysis buffer, and protein was eluted with a buffer of 20 mM sodium phosphate, 250 mM sodium chloride, and 220 mM imidazole at pH 8.0. The N-terminal His-tag was cleaved by TEV protease overnight at 4°C during dialysis into lysis buffer and subsequently removed by Ni-NTA chromatography. HNH-L2 was further purified by size exclusion chromatography via a HiLoad 26/600 Superdex 200 column (GE) in a buffer of 20 mM HEPES and 200 mM potassium chloride at pH 8.0. Fractions containing purified HNH-L2 were then concentrated to 0.5 mM and dialyzed into a buffer containing 20 mM HEPES at pH 7.4 with 1 mM DTT and either 60 mM potassium chloride (for apo HNH-L2), 60 mM magnesium chloride (for Mg^{2+}-saturated HNH-L2), or calcium chloride (for Ca^{2+}-saturated HNH-L2).

NMR experiments were performed on a Bruker Avance NEO 600 MHz spectrometer at 25°C. NMR data were processed using NMRPipe[55] and analysed in Sparky[56] with in-house scripts. Backbone resonances for HNH-L2 were assigned in CARA[57] using an HSQC and triple resonance correlation experiments[58] including HNCACB, HN(CO)CACB, HN(CA)CO, HNCO, and compared to the backbone assignments of a prior HNH domain construct (BMRB Entry 27949)[59]. $^{1}H^{15}N$ combined chemical shift perturbations were determined from $^{1}H^{15}N$ HSQC spectra by $\Delta\delta = |\delta_{WT} - \delta_{variant}|$, where $\delta = \sqrt{\frac{1}{2}((\delta_{HN}^2) + (\delta_N^2/25))}$ [60]. Spin relaxation experiments were performed with the ^{1}H and ^{15}N carriers set to the water resonance and 117 pm, respectively[61]. Longitudinal relaxation rates (R_1) were measured with T_1 delays of 0, 20, 60(x2), 100, 200, 300, 600(x2), 800, and 1200 ms. Transverse relaxation rates (R_2) were measured with T_2 delays of 0, 8.48, 16.96(x2), 33.92, 50.88, 67.84(x2), 84.8, 101.76, 118.72, and 135.68(x2) ms. The recycle delay in these experiments was 1.25 s and these data were collected in a temperature-compensated manner. Longitudinal and transverse relaxation rates were extracted by nonlinear least squares fitting of the peak heights to a single exponential decay using in-house software. Uncertainties in these rates were determined from replicate spectra with duplicate relaxation delays. Steady-state ^{1}H-[^{15}N] NOE were obtained by interleaving pulse sequences with and without proton saturation and calculated from the ratio of peak heights[61]. Carr-Purcell-Meiboom-Gill (CPMG) NMR experiments were adapted from Palmer and coworkers[62], and performed at 25ºC with a constant

relaxation period of 40 ms, a 2.0 second recycle delay, and ν_{CPMG} values of 25, 50 (x2), 75, 100, 150, 250, 500 (x2), 750, 800 (x2), 900, 1000 Hz. Relaxation dispersion profiles were generated by plotting R_2 vs. $1/\tau_{cp}$ and fitting the data with in-house scripts. For pK_a determination, histidine ε_1 sidechain chemical shifts were measured by ^{1}H-^{13}C HMQC at selected pH values between pH 6 – 8[63]. pH was adjusted by the addition of hydrochloric acid and monitored with a micro pH electrode. pK_a values were calculated by fitting the ^{1}H chemical shift trajectories to a modified Henderson-Hasselbach equation $\Delta\delta = \left(\frac{\Delta\delta_{max} \times 10^{(pH-pK_a)}}{1+10^{(pH-pK_a)}}\right) + \Delta\delta_{min}$, where $\Delta\delta_{min}$ is expected to be approximately equal to zero and it is assumed that Δδ is equal to zero in the fully monoprotonated state.

S1.13. Differential Scanning Fluorimetry (DSF)

Differential scanning fluorimetry (DSF) was performed to evaluate the thermal stability of four Cas9 variants (WT SpCas9, M1, M2, and M3) under three conditions: apo, Mg^{2+}-bound, and Mg^{2+} and guide RNA-bound. Protein samples were prepared in a buffer of 20 mM HEPES, 200 mM KCl, and 1 mM DTT at pH 7.5. SYPRO Orange dye was added to a final 10x concentration immediately prior to analysis. For Mg^{2+}-containing conditions, $MgCl_2$ was added to the indicated final concentration, and guide RNA was included where specified. Thermal denaturation experiments were performed by heating samples from 25-95 °C while continuously monitoring fluorescence intensity. Thermal unfolding curves were analyzed using the publicly available analysis tools through the DSFworld platform[64]. Melting transition temperatures (T_m) were determined by fitting fluorescence profiles to sigmoidal denaturation models. Reported T_m values represent the mean ± standard deviation from quadruplicate measurements (n=4).

S2. Supplementary Results

S2.1. Ion-dependent modulation of HNH conformational activation

To characterize ion-dependent conformational responses of the HNH domain, MD simulations were performed on the pre-active Cas9 structure (PDB: 5F9R[1]) in the presence of monovalent (Li^+), divalent (Be^{2+}, Mg^{2+}, Ca^{2+}, Mn^{2+}, Co^{2+}, Zn^{2+}, Ra^{2+}), and trivalent (Al^{3+}) cations under high (10 mM) and low (10 μM) concentration conditions. Conformational changes were characterized using two structural observables: the distance between the catalytic residue H840 and the scissile phosphate (P_{SCI}), reporting on catalytic positioning, and the distance between the centre of mass of HNH and P_{SCI}, reporting on global domain movement (Supplementary Fig. 13a). For each ion, concentration-dependent effects were evaluated as differences between low- and high-concentration ensembles (Δd), such that positive values indicate closer approach to P_{SCI} under high-concentration conditions (Supplementary Fig. 13b,c). To quantify the extent to which these motions occur concurrently, an activation probability (P) was computed as the fraction of configurations satisfying both criteria. Its concentration dependence ($\Delta P = P_{HIGH} - P_{LOW}$) reports the extent to which high ion concentrations favour simultaneous sampling of catalytic positioning and domain alignment (Supplementary Fig. 13d). Details in Supplementary Methods (Section 1.4).

In the presence of Li^+, both catalytic positioning and HNH domain movement remain largely unchanged between high- and low-concentration conditions (Supplementary Fig. 13b,c), and the corresponding activation probability is negligible (Supplementary Fig. 13d). These results indicate that monovalent ions do not promote sampling of activation-like conformations under the conditions tested. Consistently, NMR HSQC titrations of the HNH–L2 construct performed at 298 K in the presence of Li^+ (Supplementary Fig. 21) show minimal perturbation of the HNH–L2 region even at ~160-fold excess ion concentration, with essentially no significant chemical shift changes observed.

In contrast, divalent ions favour sampling of activation-like conformations. Across this class, increased sampling of configurations in which the catalytic residue approaches P_{SCI} is consistently observed (Supplementary Fig. 13b), accompanied by varying degrees of domain movement toward P_{SCI} (Supplementary Fig. 13c). These effects translate into increased activation probability compared to monovalent ions, reflecting enhanced sampling of configurations in which catalytic positioning and domain alignment occur concurrently (Supplementary Fig. 13d). The extent of this coupling varies across ions, suggesting differences in the degree of synchronization between catalytic positioning and domain alignment.

Trivalent ions alter this balance. In the presence of Al^{3+}, catalytic positioning is observed, whereas HNH domain movement exhibits a smaller average shift accompanied by substantial variability (Supplementary Fig. 13b-c). Consistently, NMR HSQC titrations of the HNH–L2 construct performed at 298 K reveal significant chemical shift perturbations throughout the HNH–L2 region upon addition of Al^{3+} (Supplementary Fig. 21). However, due to the weak paramagnetic character of Al^{3+}, NMR signals became progressively broadened, limiting the titration to ~15-fold excess ion concentration before substantial signal loss occurred. This suggests that highly charged ions perturb the HNH conformational landscape in a manner distinct from divalent ions.

To further assess the functional consequences of these ion-dependent conformational effects, Cas9 DNA cleavage assays were performed in the presence of Mg^{2+}, Li^{+}, and Al^{3+} (Supplementary Fig. 14). Mg^{2+} supported efficient DNA cleavage, whereas Li^{+} and Al^{3+} did not support productive cleavage activity under the tested conditions. These findings further support the association between divalent ions and productive Cas9 activation.

Overall, these observations indicate that divalent ions promote sampling of activation-like conformations in Cas9, in agreement with single-molecule experiments[18], whereas the monovalent ion Li^{+} does not and the trivalent ion Al^{3+} perturbs this behaviour. Finally, we note that modelling ions with higher charge density or more specific coordination preferences (e.g., Be^{2+}, Al^{3+}, Zn^{2+}) within classical force fields presents known challenges[65]; accordingly, results for these ions are interpreted qualitatively, focusing on relative trends rather than absolute quantitative differences.

S3. Supplementary Figures

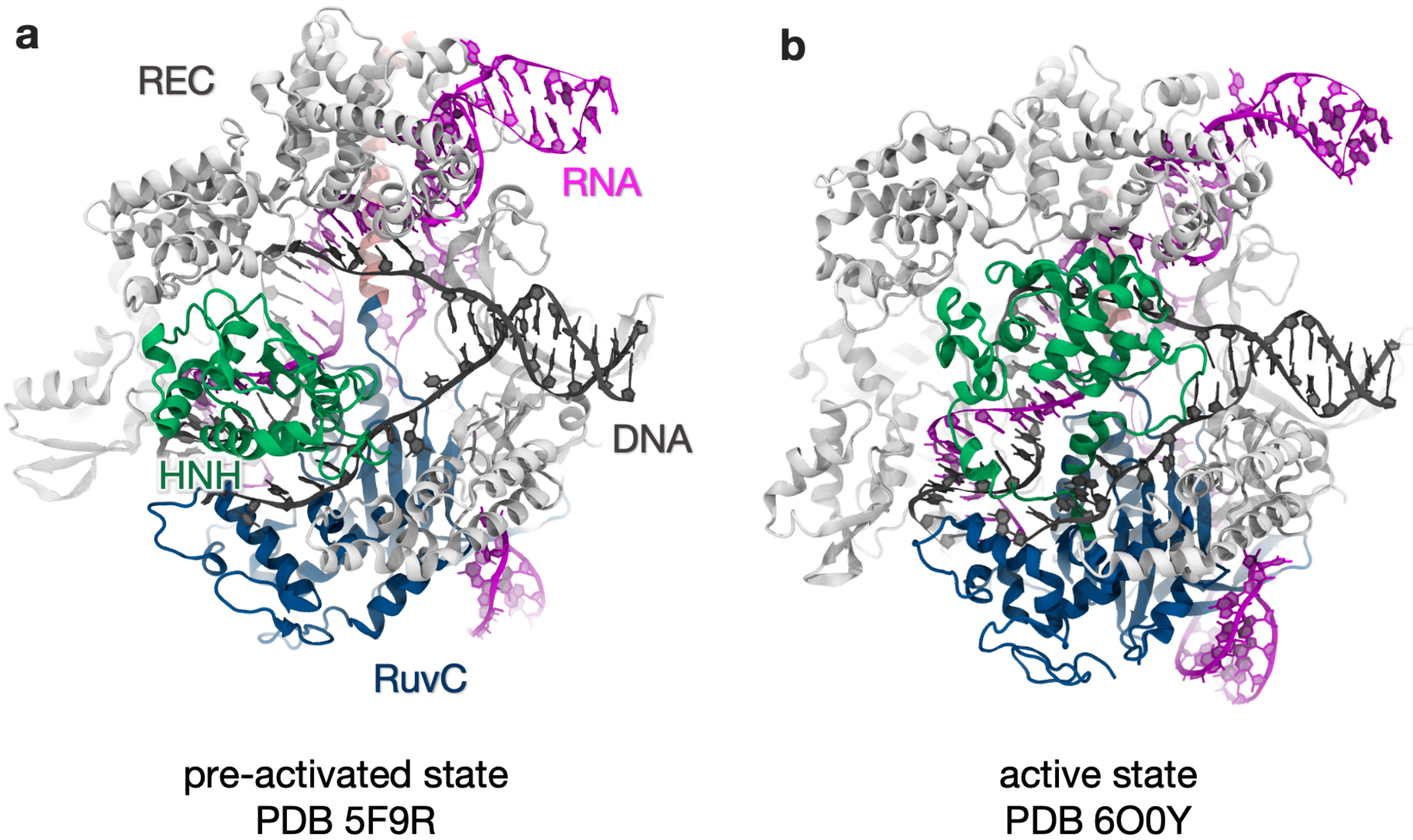

Supplementary Figure 1. Structural comparison of the *S. Pyogenes* Cas9 in the pre-activated and active conformational states. a. Structure of the Cas9 enzyme in its pre-activated conformation determined via X-ray crystallography at a resolution of 3.40 Å (PDB 5F9R)[1]. In this state, the HNH domain is approximately 14 Å away from the scissile phosphate on the target DNA strand, indicating a poised configuration that is not yet fully prepared for catalysis. **b.** Structure of the active conformation of Cas9 resolved using cryo-electron microscopy (cryo-EM) at a resolution of 3.37 Å (PDB 6O0Y)[2]. The HNH domain is docked at the target DNA strand, representing the enzyme's fully activated form.

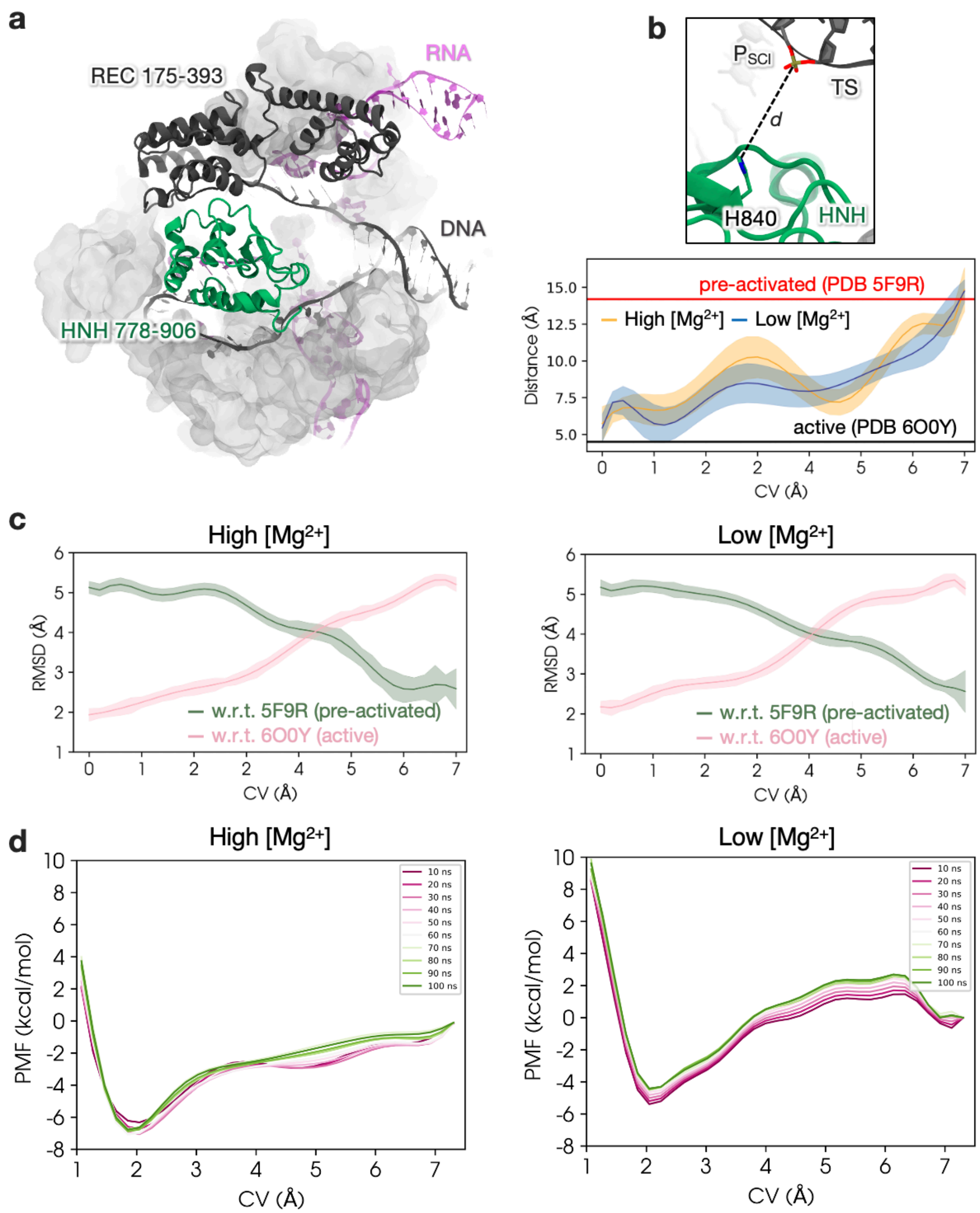

Supplementary Figure 2. Details on Umbrella Sampling (US) simulations of the HNH conformational transition. a. Overview of the *S. pyogenes* Cas9 (PDB 5F9R)[1], highlighting regions of the HNH domain (residues 778-906, green) and recognition lobe (REC, residues 175-393, black) used to define the reaction coordinate (RC) in US simulations. The RC was set as the difference in root-mean-square deviation (RMSD) of the Cα atoms in the above-mentioned HNH and REC regions relative to the pre-activated (PDB 5F9R)[1] and active (PDB 6O0Y)[2] states. **b.** The distance *d* between the HNH catalytic residue (H840, Nε) and the scissile phosphate (P_{SCI}) on the DNA target strand (TS) is plotted along the RC to demonstrate the movement of the HNH

domain from the pre-activated to the active state at high (10 mM, yellow) and low (10 μM, blue) concentration of Mg^{2+}. The reference distances in the experimental structures are indicated using red (pre-activated) and black (active) lines. Error bar represents the standard deviation of each US bin. **c.** Conformations of the Cas9 protein relative to experimental structures. The RMSD of the Cas9 backbone atoms was calculated with respect to the pre-activated (5F9R, green) and active (6O0Y, pink) experimental structures and plotted along the RC of US simulations conducted under high (left) and low (right) Mg^{2+} conditions. Error bar represents the standard deviation of each US bin. At RC ~7 Å (pre-activated state), the RMSD with respect to 5F9R is low, reflecting the system's resemblance to the pre-activated state, while the RMSD with respect to 6O0Y is high. Conversely, at RC ~2 Å (active state), the trend reverses, with low values of the with respect to 6O0Y, indicating the system's transition to the active state. While sampling from RC ~7 Å (pre-activated state) to RC ~2 Å (active state), the conformational transition is evident, as demonstrated by the progression of the distance *d* (panel b) and of the RMSD. These results confirm successful sampling from the pre-activated to the active state. **d.** Convergence of the Potential of Mean Force (PMF, in kcal/mol) relative to the HNH transition from the pre-activated to the active states at high (left) and low (right) Mg^{2+} concentrations. The PMFs were computed over the last ~100 ns of simulations per each window, for sampling from 10 to 100 ns, in steps of 10 ns, after removing the biases incurred by the restraints during the sampling (details in Supplementary Methods). The sampling converges efficiently for both systems, stabilizing the free energy profiles over the sampled RC range.

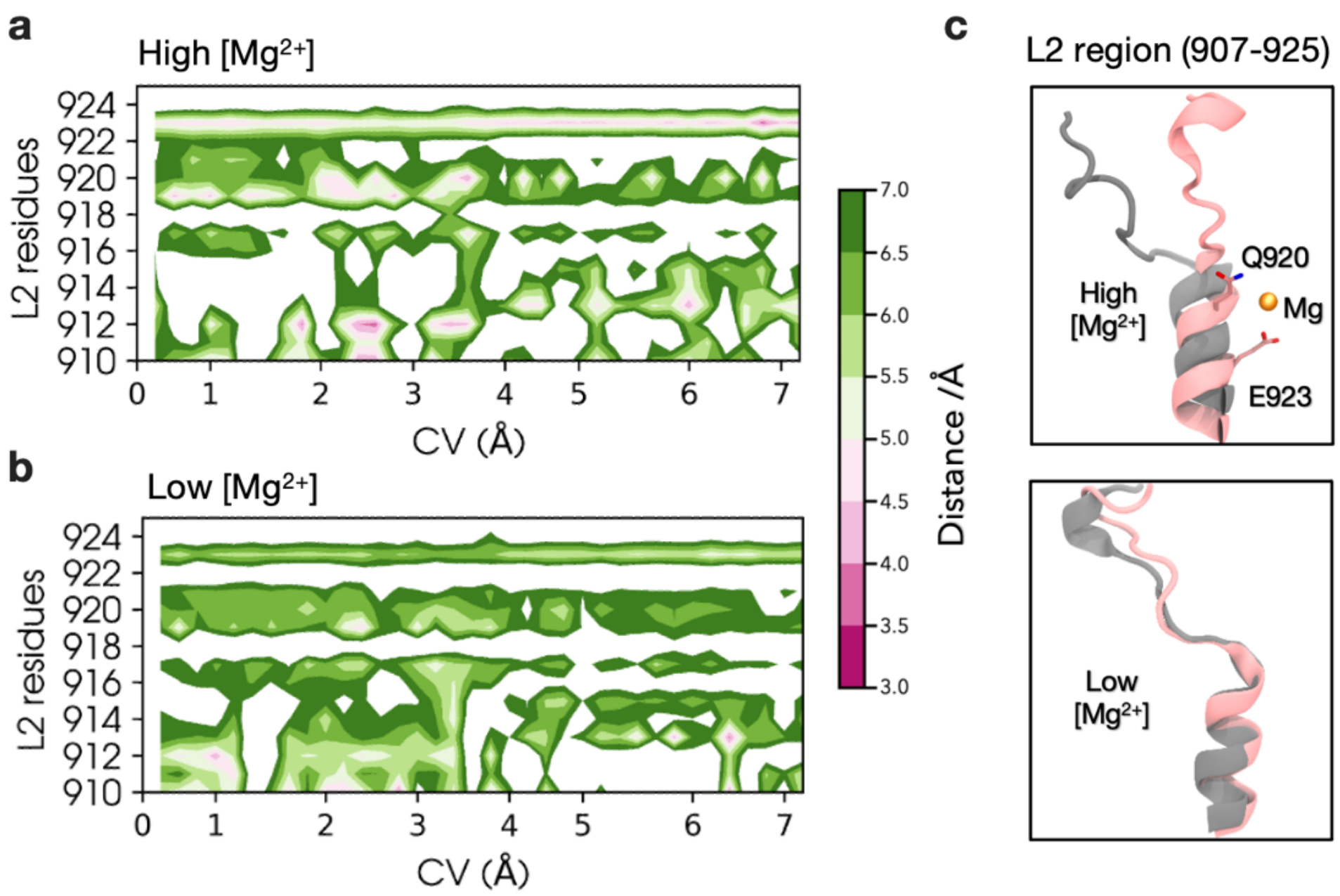

Supplementary Figure 3. Interactions of the L2 region with Mg^{2+} along the HNH conformational transition. a-b. The minimum distances between the residues of the L2 region (residues 907-925) and the nearest Mg^{2+} ion are plotted along the Reaction Coordinate (RC) of Umbrella Sampling simulations at high (10 mM, **a**) and low (10 µM, **b**) concentration of Mg^{2+}. Distances are coloured according to the scale on the right. The RC spans values from ~7 Å, corresponding to the pre-activated state, to ~2 Å, reflecting the active state (details in Supplementary Fig. 2). The L2 region interacts more frequently with Mg^{2+} at high ionic concentration, facilitating smoother conformational transitions (Fig. 1, main text). **c.** Snapshots of the L2 region, captured as HNH moves toward the active state (i.e., in the transition region from RC ~7 to ~4 Å) at high (top) and low (bottom) concentration of Mg^{2+}. At high Mg^{2+} concentration, the interactions between the L2 residues Q920, E923, and Mg^{2+} are shown, illustrating their role in the noted conformational changes.

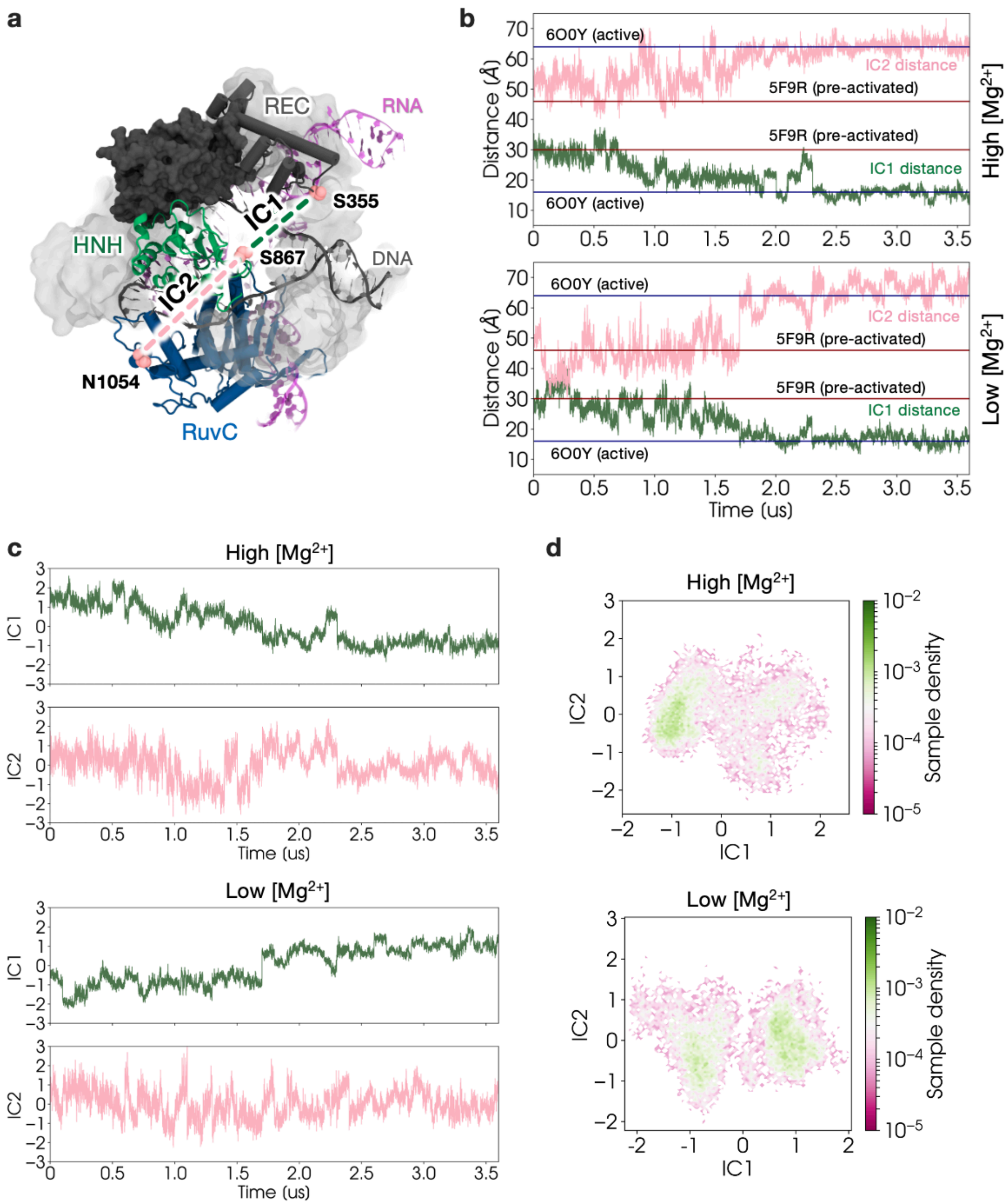

Supplementary Figure 4. Construction of Markov State Models (MSM) of the HNH conformational transition. a. Structure of the pre-activated Cas9 (PDB 5F9R[1]) showing the two distances used as features (i.e., independent components, IC) for building an MSM of the HNH transition: the distance between the Cα atoms of residues S355 and S867 (IC1), and residues S786 and N1054 (IC2). These distances were used in Förster Resonance Energy Transfer

(FRET) measurements to describe the transition of Cas9 from an inactive to an active state[18,29]. **b.** Time evolution of the selected features, i.e., Cα–Cα distances between S355–S867 (IC1, green) and S786–N1054 (IC2, pink) over the simulations at high (10 mM, top) and low (10 μM, bottom) concentrations of Mg^{2+}. Two horizontal lines indicate the reference values in the experimental structures of the pre-activated (PDB 5F9R) and active (PDB 6O0Y) states, indicating that the two distances represent conformations of HNH in the pre-active and active states. The two distances have been used as independent components (IC1 and IC2) for Time-lagged Independent Component Analysis (TICA) using a time lag of 10 ns (details in Supplementary Fig. 5), and to construct the MSM. **c.** Time evolution of IC1 (green) and IC2 (pink) over the simulations at high (top) and low (bottom) Mg^{2+} levels. **d.** Sample density distribution in the TICA space (i.e., IC1 vs IC2), coloured according to the scale on the right, reported at high (top) and low (bottom) concentration of Mg^{2+}. Density plots indicate the conformational states sampled during the simulations and highlight the differences in the population states under different of Mg^{2+} levels.

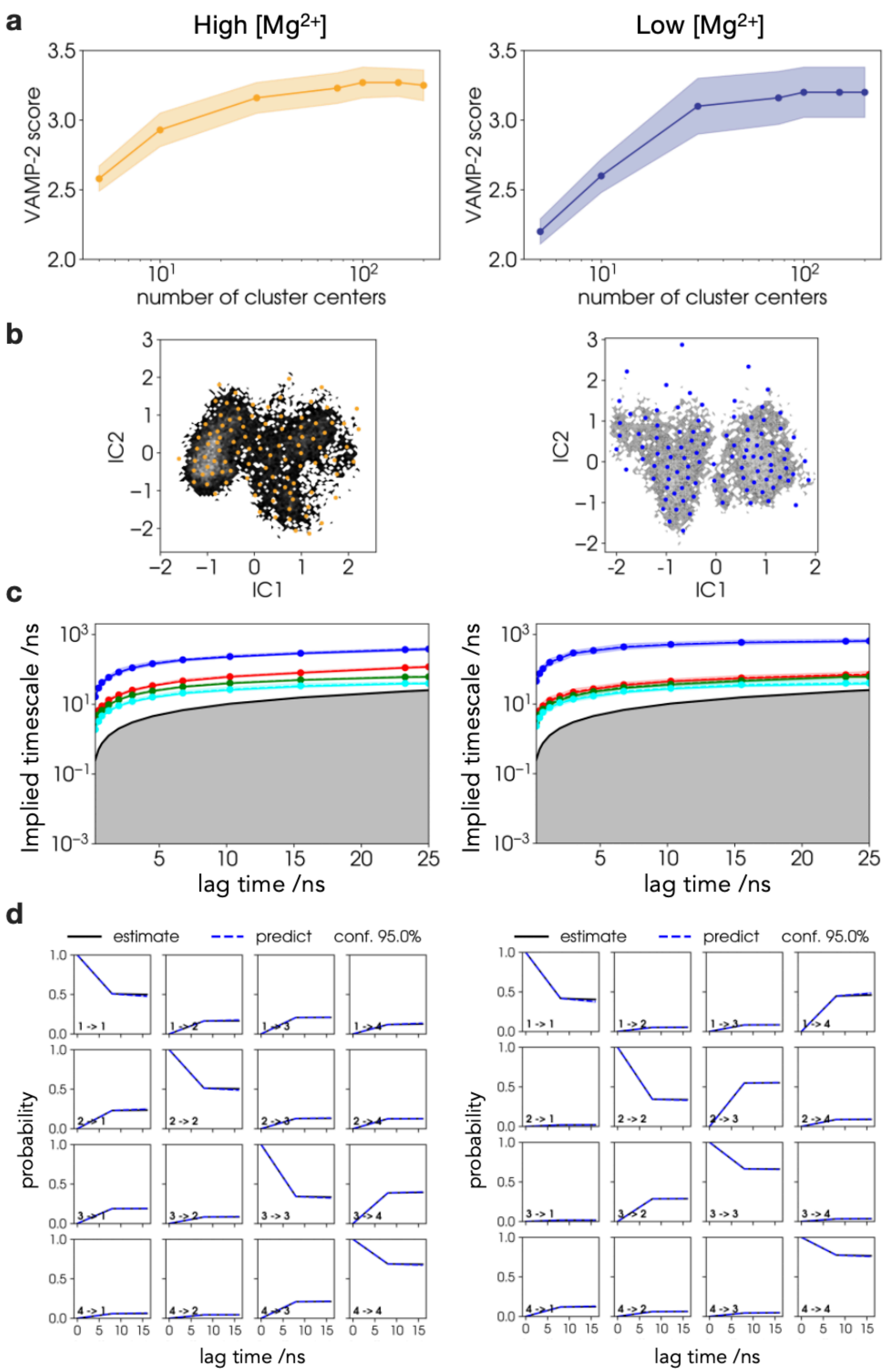

Supplementary Figure 5. Validation of Markov State Models (MSM) at high (10 mM, left) and low (10 μM, right) concentrations of Mg^{2+}. a. The VAMP-2 score is plotted as a function of the number of cluster centres. Showing the mean VAMP-2 scores, while the shaded regions indicate

the lower-to-upper range of the corresponding PyEMMA score estimates. At both concentrations of Mg^{2+}, the VAMP-2 score increases with the number of clusters, indicating that the models can predict the long timescale kinetics from molecular dynamics simulations [28]. The optimal number of clusters is observed at ~10^2, where the VAMP-2 score reaches a plateau, indicating a trade-off between model accuracy and computational efficiency. **b.** Clusters in the TICA space (i.e., IC1 vs. IC2 plots) showing the density and distribution of sampled conformational states under high (left) and low (right) concentrations of Mg^{2+}. **c.** The implied timescale is plotted as a function of the lag time at high (left) and low (right) Mg^{2+} levels. These plots are used to determine the optimal lag time for the MSM. The timescales stabilize around ~10 ns for both systems, indicating this as the suitable lag time for constructing the MSM. The stabilization of the timescales confirms the reliability and robustness of the MSM for capturing the slow dynamics of the system. **d.** Chapman-Kolmogorov test for MSM building at high (left) and low (right) concentrations of Mg^{2+}. This test compares the predicted and estimated probabilities of state transitions across different lag times. The close agreement between the predicted (solid lines) and estimated (dashed lines) probabilities demonstrate the accuracy of the MSM. Each subplot corresponds to a different state transition, demonstrating the model's capability to reliably predict the system's long-term dynamics. Overall, the analysis in panels **a-b** suggests that clustering with ~100 centres provide the best performance under both high and low Mg^{2+} concentrations. The implied timescale plots (panel **c**) validate the choice of a 10 ns lag time for the MSM, ensuring that the model captures the essential dynamics of Cas9. The Chapman-Kolmogorov test (panel **d**) further supports the MSM's accuracy, showing that the model's predictions align well with the observed transitions. This comprehensive validation indicates that the MSMs are well-constructed and reliable for studying the conformational dynamics of Cas9 under different concentrations of Mg^{2+}.

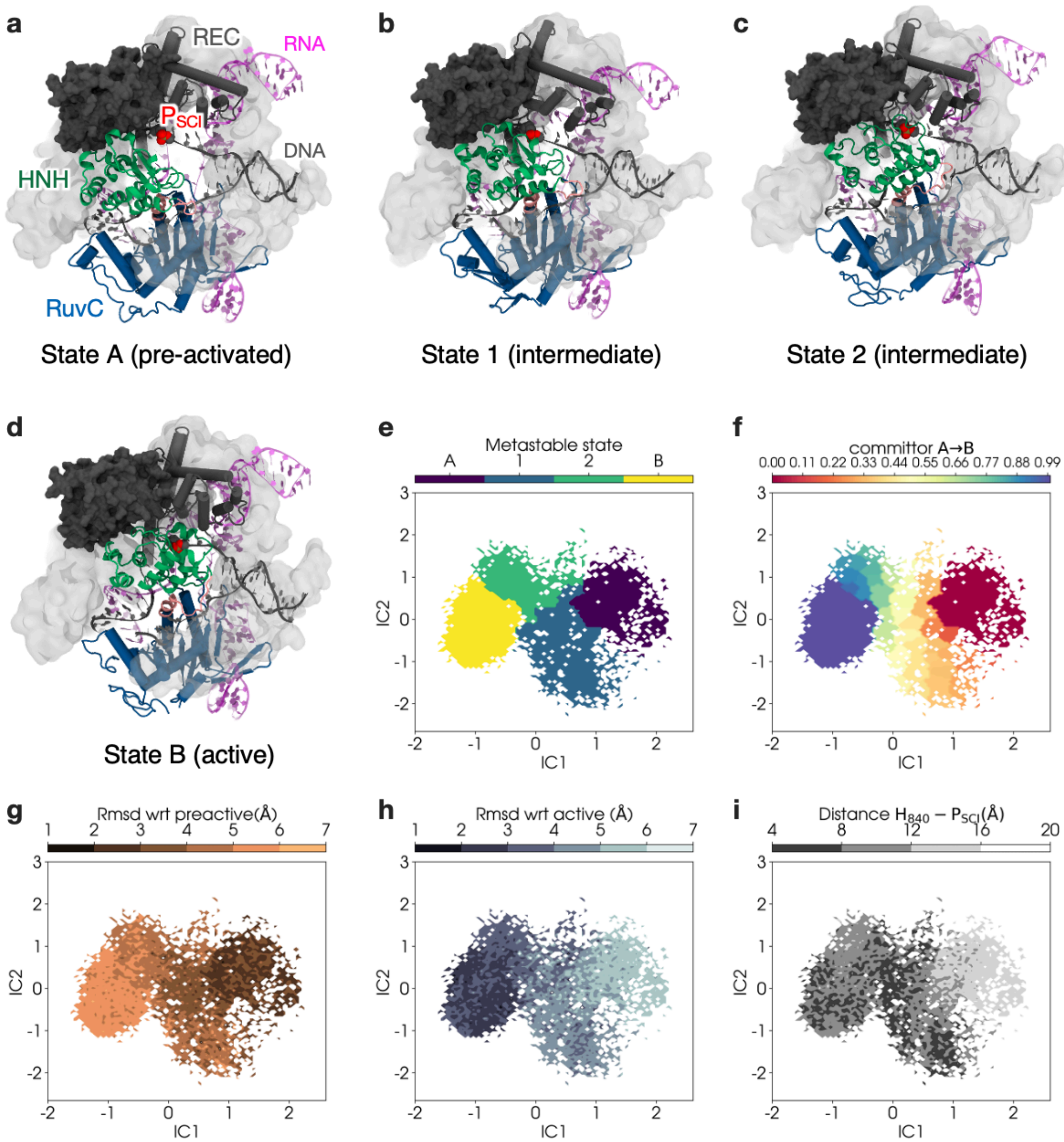

Supplementary Figure 6. Structural analysis of Cas9 metastable states from Markov State Models (MSM) at high concentration (10 mM) of Mg^{2+}. a-d. Conformational states of Cas9 identified from the MSM. **a.** State A, displaying the HNH domain in a pre-activated conformation (as in PDB 5F9R). **b.** State 1, metastable intermediate. **c.** State 2, metastable intermediate. **d.** State B, the HNH domain assumes an active conformation (as in PDB 6O0Y). **e.** TICA space (i.e., IC1 vs. IC2 plots) color-coded according to the metastable states identified through MSM. A Perron Cluster Cluster Analysis (PCCA++) was used to separate the conformational space into distinct metastable states by analysing the eigenvectors of the transition matrix (details in Supplementary Methods). States are labelled as A (pre-activated, purple), 1 (intermediate

metastable, blue), 2 (intermediate metastable, yellow), and B (active, red). **F.** TICA space color-coded according to the committor value, representing the probability of transitioning from State A to State B. Regions with high committor values (red) indicate a strong likelihood of transitioning to State B, while values near 0.5 signify an equal probability of transitioning to either State A or State B, identifying these regions as metastable intermediates, which facilitate the transition. **g.** TICA space color-coded according to the root-mean-square deviation (RMSD) of the protein backbone atoms with respect to the experimental structure of the pre-activated Cas9 (PDB 5F9R)[1]. High RMSD values indicate greater deviation from the pre-activated state. **h.** TICA space color-coded according to the RMSD of the protein backbone atoms concerning the experimental structure of the active Cas9 (PDB 6O0Y)[2]. High RMSD values indicate greater deviation from the active state. **i.** TICA space color-coded according to the distance between the catalytic residue H840 (Nε) and the scissile phosphate (P_{SCI}). This distance serves as a key metric for the conformational state of the HNH domain, where shorter distances indicate a state closer to the active conformation required for catalysis. This comprehensive analysis elucidates the transition pathway of Cas9 from the pre-activated to the active state.

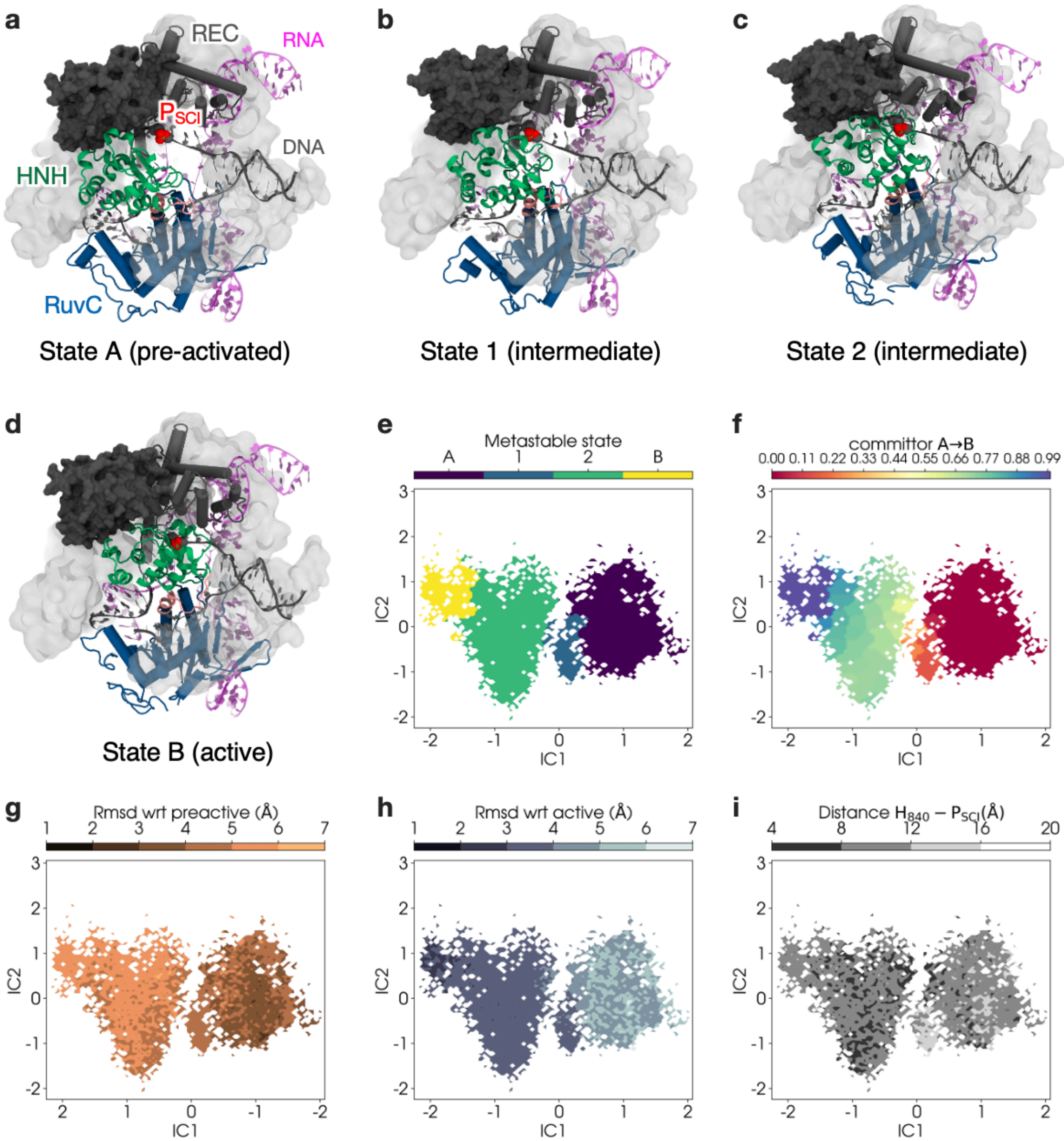

Supplementary Figure 7. Structural analysis of Cas9 metastable states from Markov State Models (MSM) at low concentration (10 µm) of Mg^{2+}. a-d. Conformational states of Cas9 identified from the MSM. **a.** State A, displaying the HNH domain in a pre-activated conformation (as in PDB 5F9R)[1]. **b.** State 1, metastable intermediate. **c.** State 2, metastable intermediate. **d.** State B, the HNH domain assumes an active conformation (as in PDB 6O0Y)[2]. **e.** TICA space (i.e., IC1 vs. IC2 plots) color-coded according to the metastable states identified through MSM. A Perron Cluster Cluster Analysis (PCCA++) was used to separate the conformational space into distinct metastable states by analysing the eigenvectors of the transition matrix (details in Supplementary Methods). States are labelled as A (pre-activated, purple), 1 (intermediate

metastable, blue), 2 (intermediate metastable, yellow), and B (active, red). **F.** TICA space color-coded according to the committor value, representing the probability of transitioning from State A to State B. High committor values (red) indicate a high likelihood of transitioning to State B. Regions with high committor values (red) indicate a strong likelihood of transitioning to State B, while values near 0.5 signify an equal probability of transitioning to either State A or State B, identifying these regions as metastable intermediates, which facilitate the transition. **g.** TICA space color-coded according to the root-mean-square deviation (RMSD) of the protein backbone atoms with respect to the experimental structure of the pre-activated Cas9 (PDB 5F9R)[1]. High RMSD values indicate greater deviation from the pre-activated state. **h.** TICA space color-coded according to the RMSD of the protein backbone atoms concerning the experimental structure of the active Cas9 (PDB 6O0Y)[2]. High RMSD values indicate greater deviation from the active state. **i.** TICA space color-coded according to the distance between the catalytic residue H840 (Nε) and the scissile phosphate (P_{SCI}). This distance serves as a key metric for the conformational state of the HNH domain, where shorter distances indicate a state closer to the active conformation required for catalysis. This comprehensive analysis elucidates the transition pathway of Cas9 from the pre-activated to the active state.

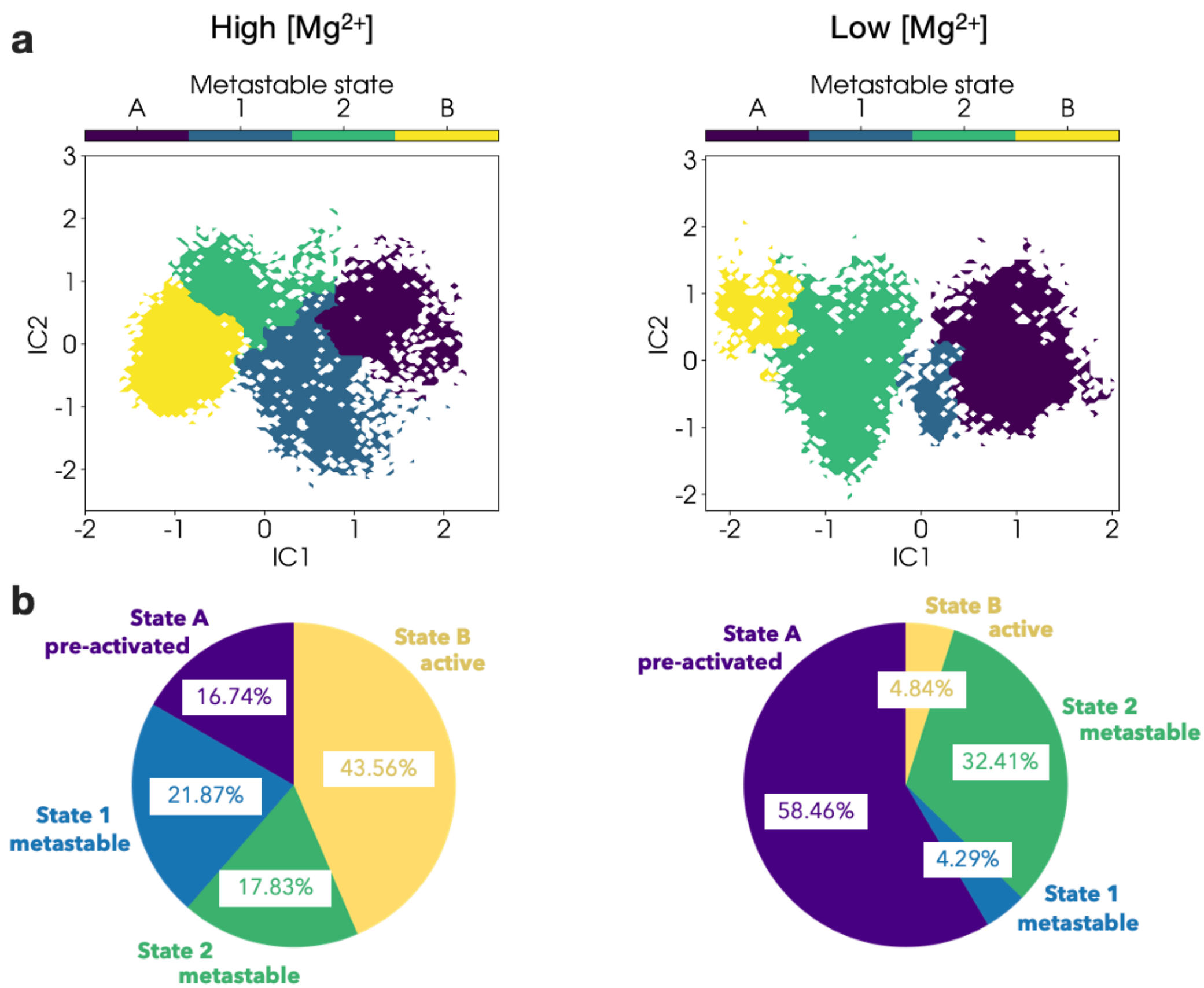

Supplementary Figure 8. Population of the metastable states from Markov State Models (MSM) at high (10 mM, left) and low (10 μM, right) concentrations of Mg^{2+}. a. TICA space (i.e., IC1 vs. IC2 plots) color-coded according to the metastable states identified through MSM. A Perron Cluster Cluster Analysis (PCCA++) was used to separate the conformational space into distinct metastable states by analysing the eigenvectors of the transition matrix (details in Supplementary Methods). States are labelled as A (pre-activated, purple), 1 (intermediate metastable, blue), 2 (intermediate metastable, yellow), and B (active, red). **b.** Pie chart showing the population (in percentage) of each state obtained from MSM analysis at high (left) and low (right) concentration of Mg^{2+}.

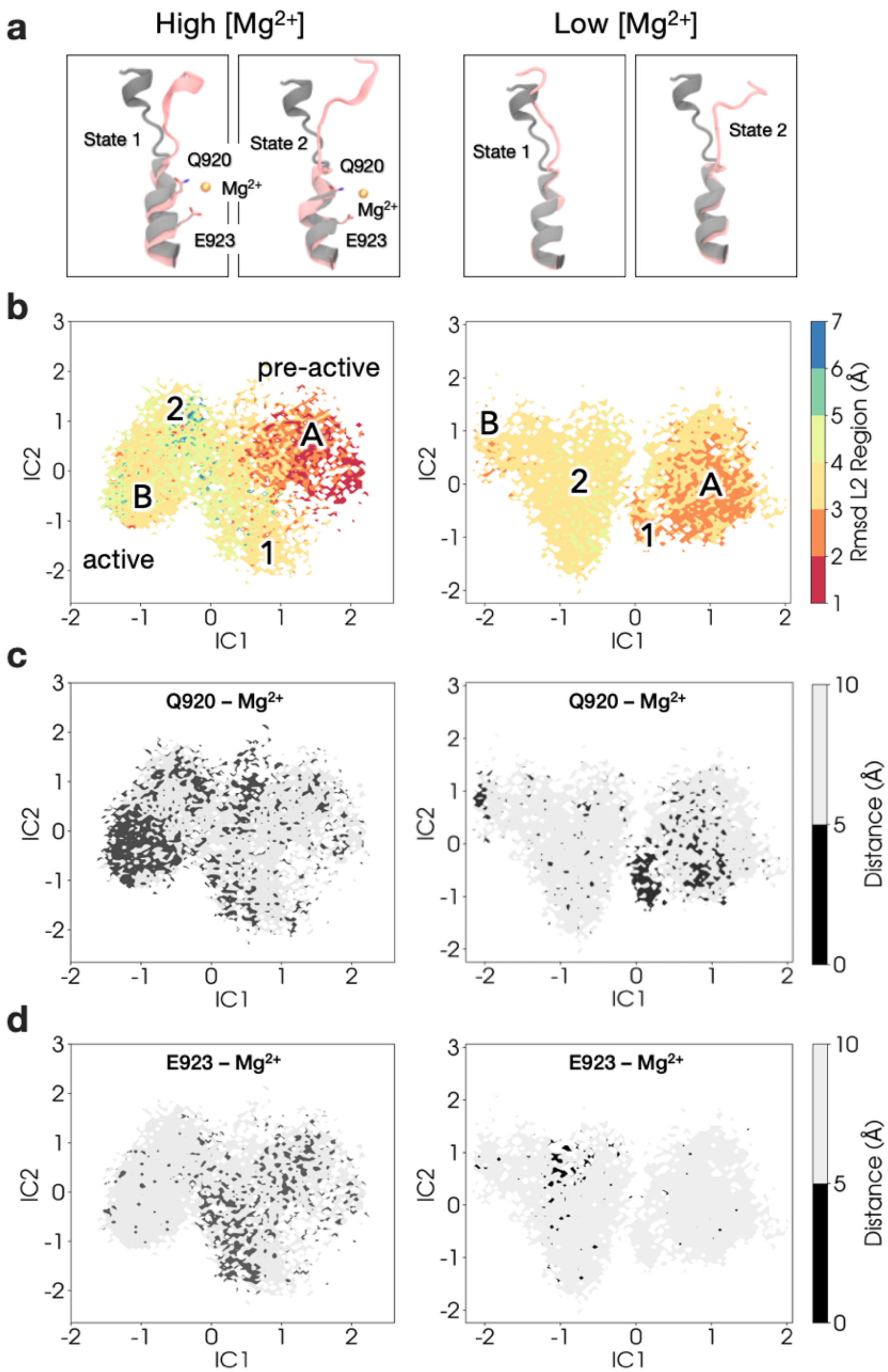

Supplementary Figure 9. Conformations of the L2 region from Markov State Models (MSM) at high (10 mM, left) and low (10 µM, right) concentrations of Mg^{2+}. a. Representative conformations of the L2 region (residues 907-925) observed in two intermediate metastable states (state 1 and state 2) from MSMs. At high concentration of Mg^{2+} (left), the Q920 and E923 residues interact with Mg^{2+}, while these interactions are absent at low concentration of Mg^{2+} (right). **b.** TICA space (i.e., IC1 vs. IC2 plots) coloured based on the root-mean-square deviation (RMSD) of the

L2 region. States are labelled as A (pre-activated), 1 and 2 (intermediate metastable), and B (active). Under high Mg^{2+} concentration (left), the L2 region shows a decrease in stability as HNH transitions from the intermediate states to the active state. **c-d.** Interactions involving the Q920 (**c**) and E923 (**d**) residues and their nearest Mg^{2+} ions (quantified considering the side-chain oxygens (OE1) of both residues and Mg^{2+}), plotted within the TICA space. At high concentration of Mg^{2+} (left), L2 interacts more frequently with Q920 and E923 during the HNH transition, compared to low Mg^{2+} levels (right).

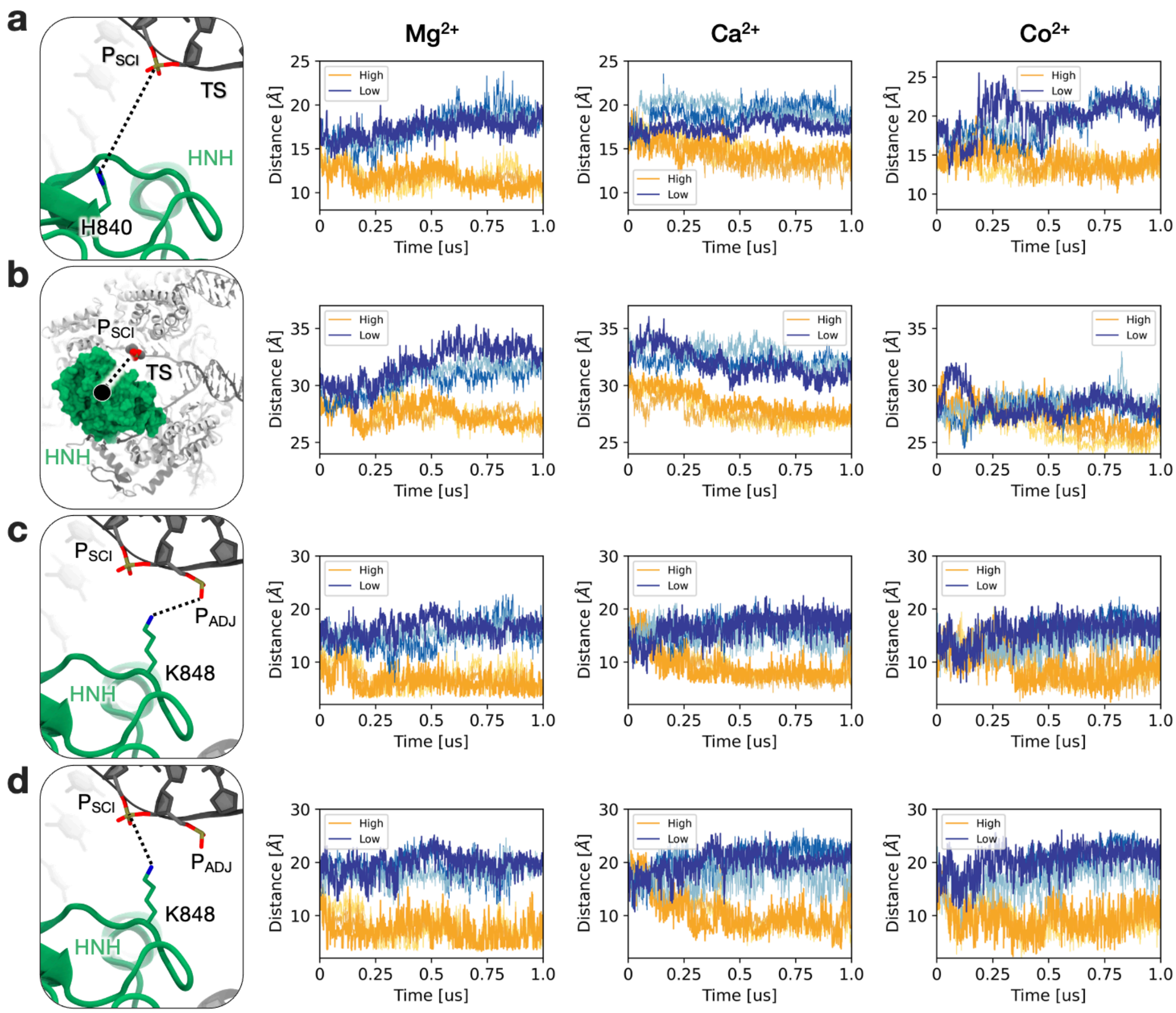

Supplementary Figure 10. Analysis of key distances bridging the HNH catalytic core and the DNA target strand (TS) under high (10 mM) and low (10 μM) concentrations of divalent metals. Structural representations displaying the distance measurements between key residues of HNH and the scissile phosphate (P_{SCI}) over MD simulations of the pre-active Cas9 (PDB 5F9R[1]) at high (yellow) and low (blue) concentrations of divalent ions: Mg^{2+} (second column), Ca^{2+} (third column), and Co^{2+} (fourth column). Data are shown for three independent replicates of ~1 μs each, for each ionic concentration. Structural representations and measurements are reported for the distances between the catalytic residue H840 and P_{SCI} (**a**), the centre of mass of the HNH domain and the P_{SCI} (**b**), K848 and the phosphate adjacent to P_{SCI} (P_{ADJ}) (**c**), K848 and P_{SCI} (**d**). These data indicate that high concentrations of divalent metal ions favour the transition towards the active state.

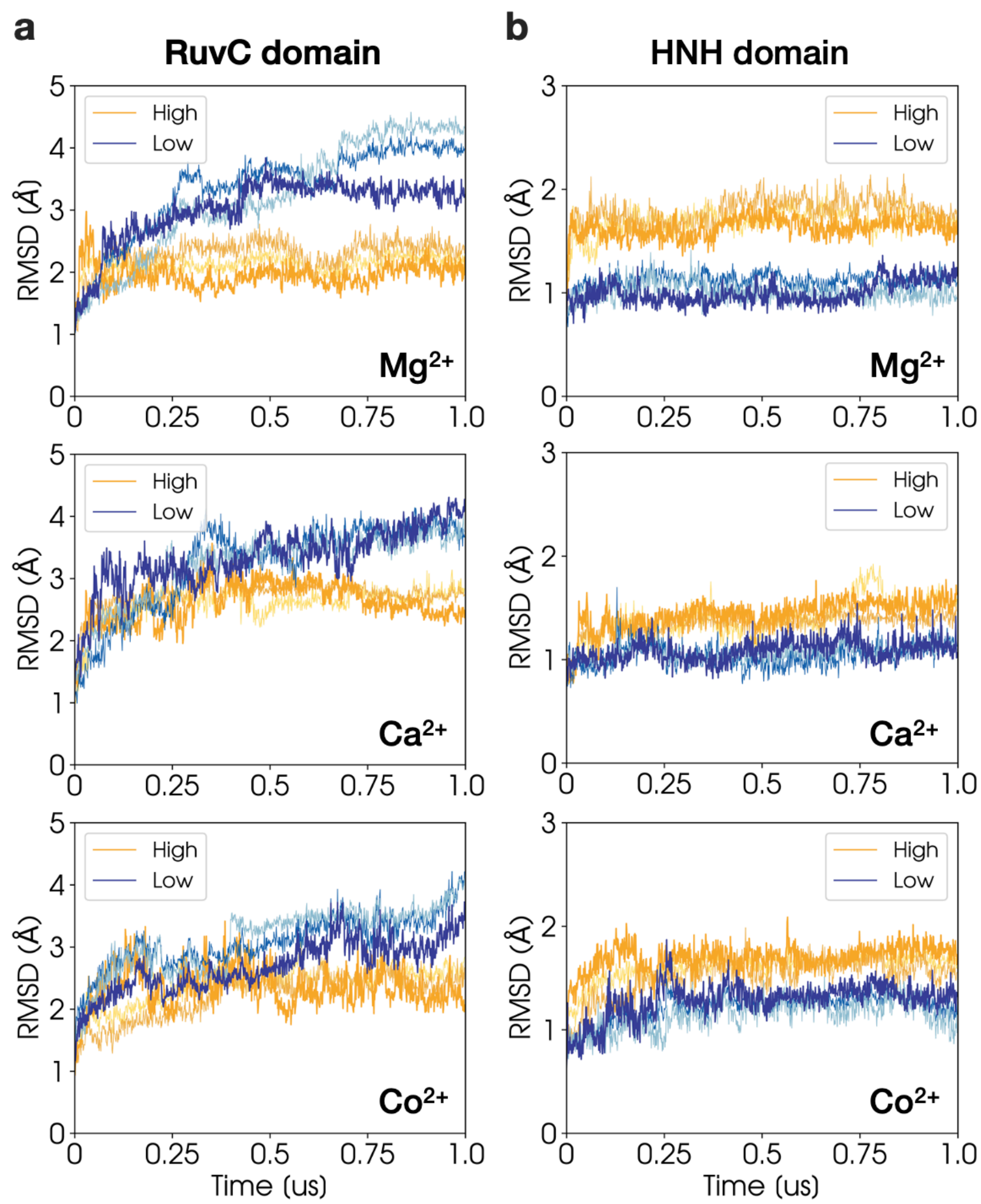

Supplementary Figure 11. Stability of the HNH and RuvC domains under high (10 mM) and low (10 μM) concentrations of divalent metals. Backbone root-mean-square deviation (RMSD) of the RuvC (**a**) and HNH (**b**) domains over MD simulations of the pre-active Cas9 (PDB 5F9R)[1] at high (yellow) and low (blue) concentrations of divalent ions: Mg^{2+} (first row), Ca^{2+} (second row), and Co^{2+} (third row). Data are shown for three independent replicates of ~1 μs each, for each ionic concentration. The data indicate that high concentrations of divalent ions stabilize the RuvC domain (**a**), while destabilizing the HNH domain (**b**). The increase in dynamics of the HNH domain at high ionic concentration supports the conformational changes necessary for the HNH domain to reach the active state.

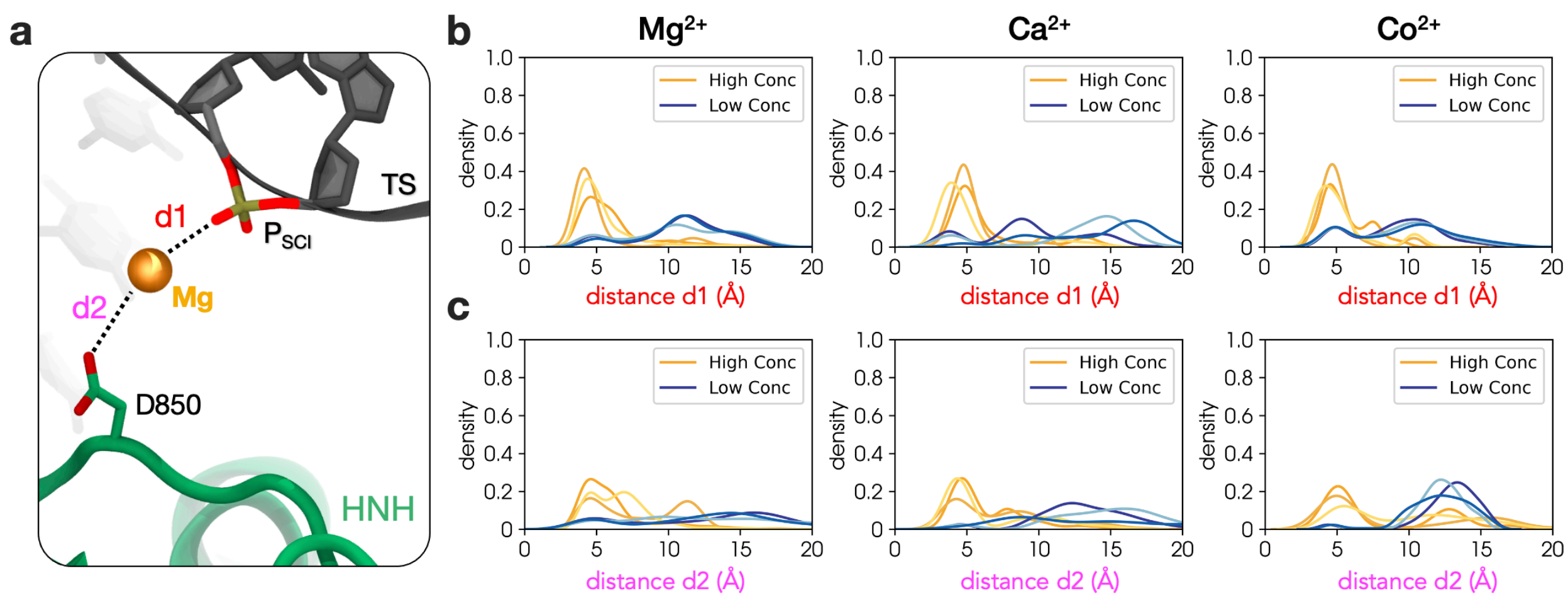

Supplementary Figure 12. Analysis of the interactions established by divalent metal ions within the HNH catalytic pocket. a. Overview of the HNH catalytic core showing the interactions established by divalent metals (Mg^{2+}, Ca^{2+} or Co^{2+}) with the scissile phosphate (P_{SCI}) on the DNA TS (distance d1), and with the D850 residue of HNH (distance d2). **b-c**. Probability distribution of the d1 (**b**) and d2 (**b**) distances over MD simulations of the pre-active Cas9 (PDB 5F9R[1]) at high (10 mM, yellow) and low (10 μM, blue) concentrations of divalent ions: Mg^{2+} (first column), Ca^{2+} (second column), and Co^{2+} (third column). Data are shown for three independent replicates of ~1 μs each, for each ionic concentration. This analysis indicates that, at high ionic concentration, the divalent ion forms bridging interactions between D850 and P_{SCI}, facilitating the transition of the HNH domain towards the active state. This bridging interaction is significantly less frequent or absent at low ionic concentration, underscoring the importance of divalent ions in achieving the active state transition of Cas9. The occurrence of this bridging interaction over the simulated ensemble is reported in Fig. 3c (main text) for Mg^{2+}, and Supplementary Fig. 17 (panels e) for Ca^{2+} and Co^{2+}.

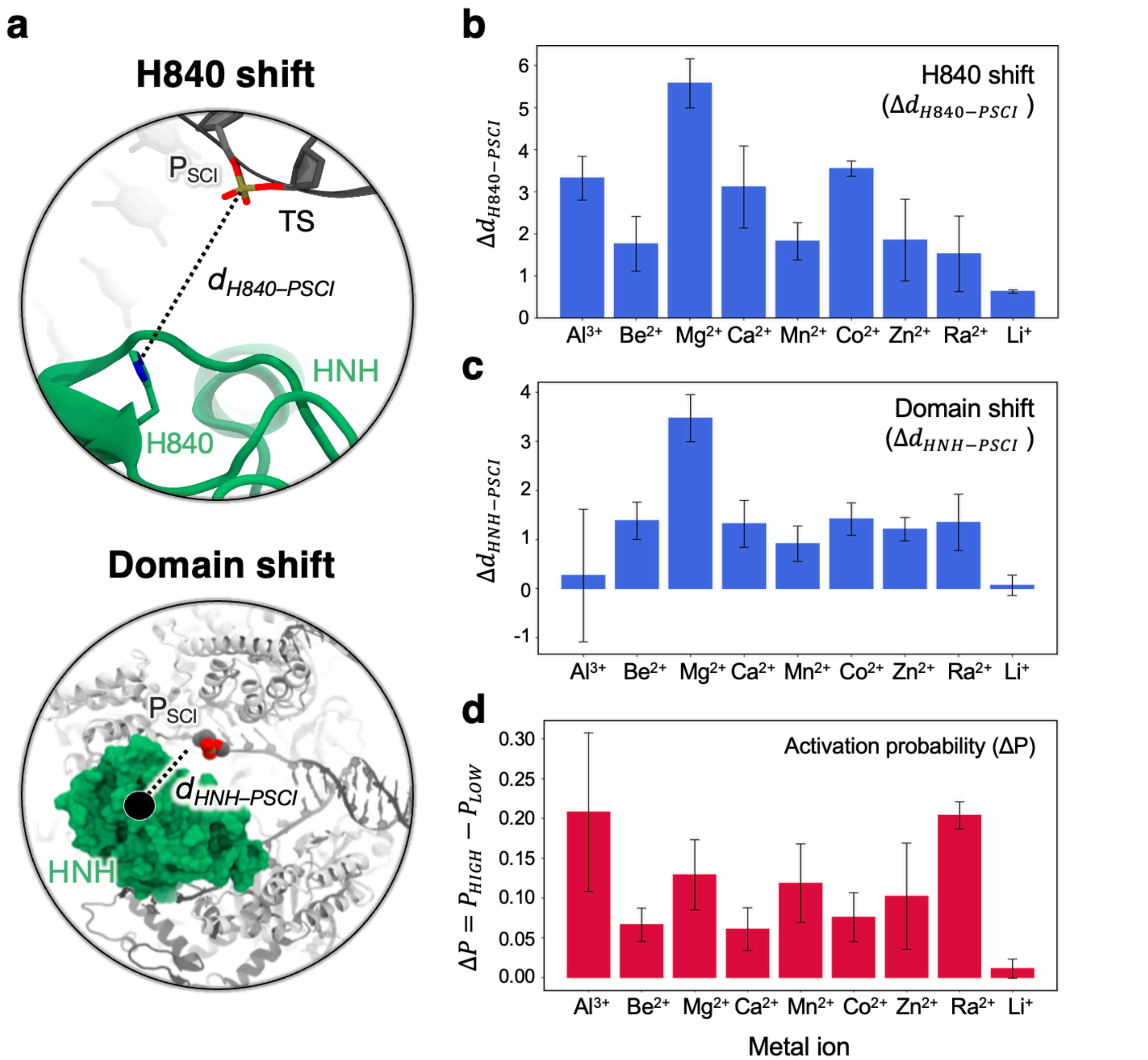

Supplementary Figure 13. Ion-dependent modulation of HNH conformational activation. a. Distances used to characterize HNH activation. *Top:* distance between the catalytic residue H840 (Cα) and the scissile phosphate (P_{SCI}) on the DNA target strand (TS), $d_{H840-PSCI}$. *Bottom:* distance between the center of mass of the HNH domain and P_{SCI}, $d_{HNH-PSCI}$ .These distances report on the positioning of the catalytic residue and global HNH domain movement, respectively. **b.** Change in catalytic residue positioning, $\Delta d_{H840\text{-}PSCI} = d^{LOW} - d^{HIGH}$,for each ion. Positive values indicate that H840 is positioned closer to PSCI under high-concentration conditions. **c.** Change in HNH domain positioning, $\Delta d_{HNH\text{-}PSCI} = d^{LOW} - d^{HIGH}$ .Positive values indicate closer approach of the HNH domain to P_{SCI} under high-concentration conditions. **d.** Change in activation probability, $\Delta P = P_{\text{HIGH}} - P_{\text{LOW}}$, where P is the fraction of configurations in which both $d_{\text{H840-PSCI}}$ and $d_{\text{HNH-PSCI}}$ fall below ion-specific thresholds defined from the high-concentration ensemble (25th percentile). Positive values indicate increased sampling of configurations in which catalytic positioning and domain alignment toward P_{SCI} occur simultaneously under high-concentration conditions. All

quantities were computed from molecular dynamics simulations of the pre-active Cas9 structure (PDB: 5F9R)[1] for monovalent (Li^{+}), divalent (Be^{2+}, Mg^{2+}, Ca^{2+}, Mn^{2+}, Zn^{2+}, Ra^{2+}), and trivalent (Al^{3+}) cations, using three independent ~1 µs replicas per ion and concentration condition (10 mM and 10 µM). For each ion and concentration condition, three ~1 µs replicas were analysed ($n = 3$ independent simulations per condition). Error bars in panels b–d represent propagated standard errors for the difference between the independently averaged high- and low-concentration ensembles, as described in Supplementary Methods and Supplementary Results.

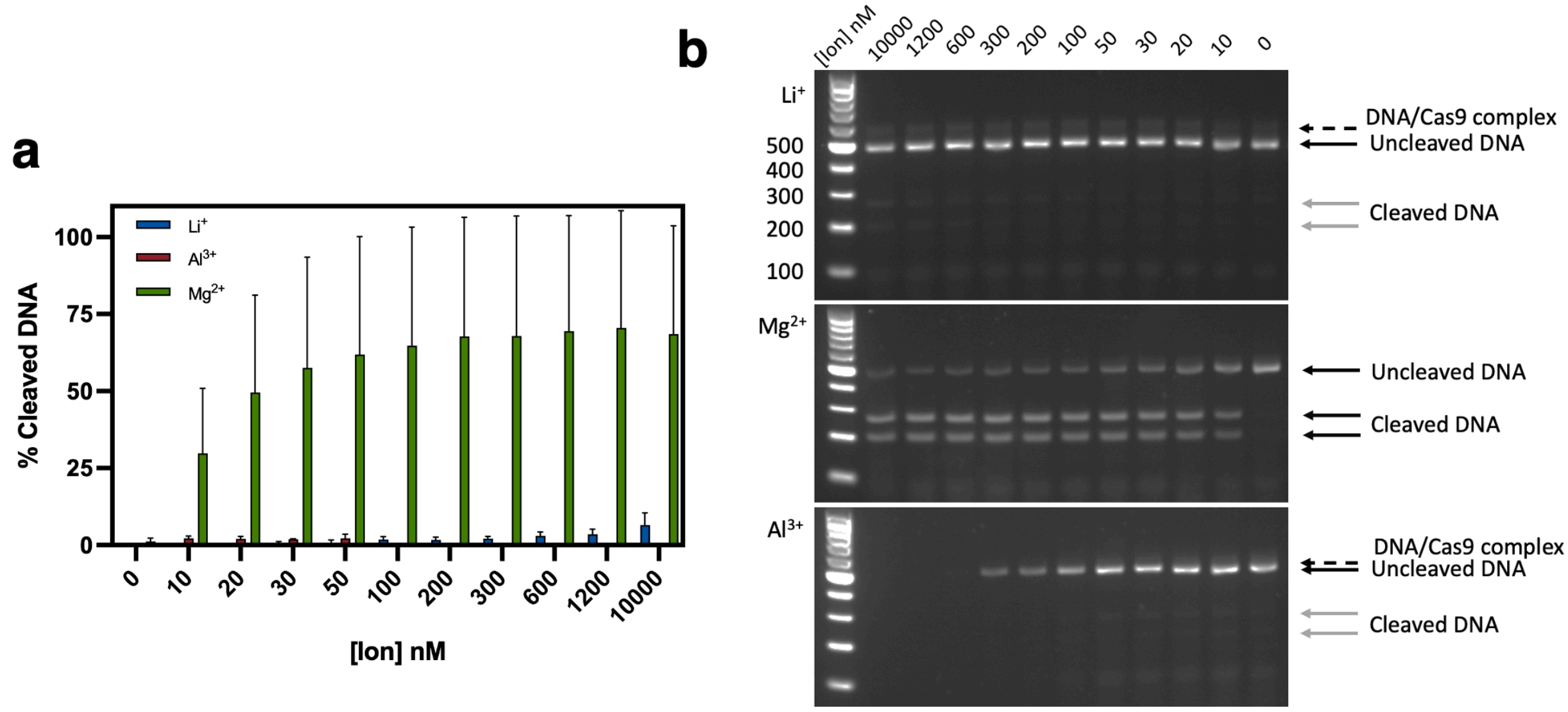

Supplementary Figure 14. ***In vitro*** **DNA cleavage assays testing the effect of Mg^{2+}, Li^{+}, and Al^{3+} on Cas9 activity. a.** Bar graph quantifying the DNA cleavage via band intensities from the agarose gel. Bars represent the mean ± s.d. from three technical replicates (n = 3 technical replicates per condition). Li^{+} showed negligible ability to activate Cas9 cleavage compared to Mg^{2+}, while DNA possible degradation by Al^{3+} resulted in no observable DNA at all at high concentrations. Cas9 RNPs were incubated with varying concentrations of metal ions and assayed for target DNA cleavage at 37 °C. **b.** Representative agarose gels visualizing cleaved DNA substrate in the presence of each metal ion.

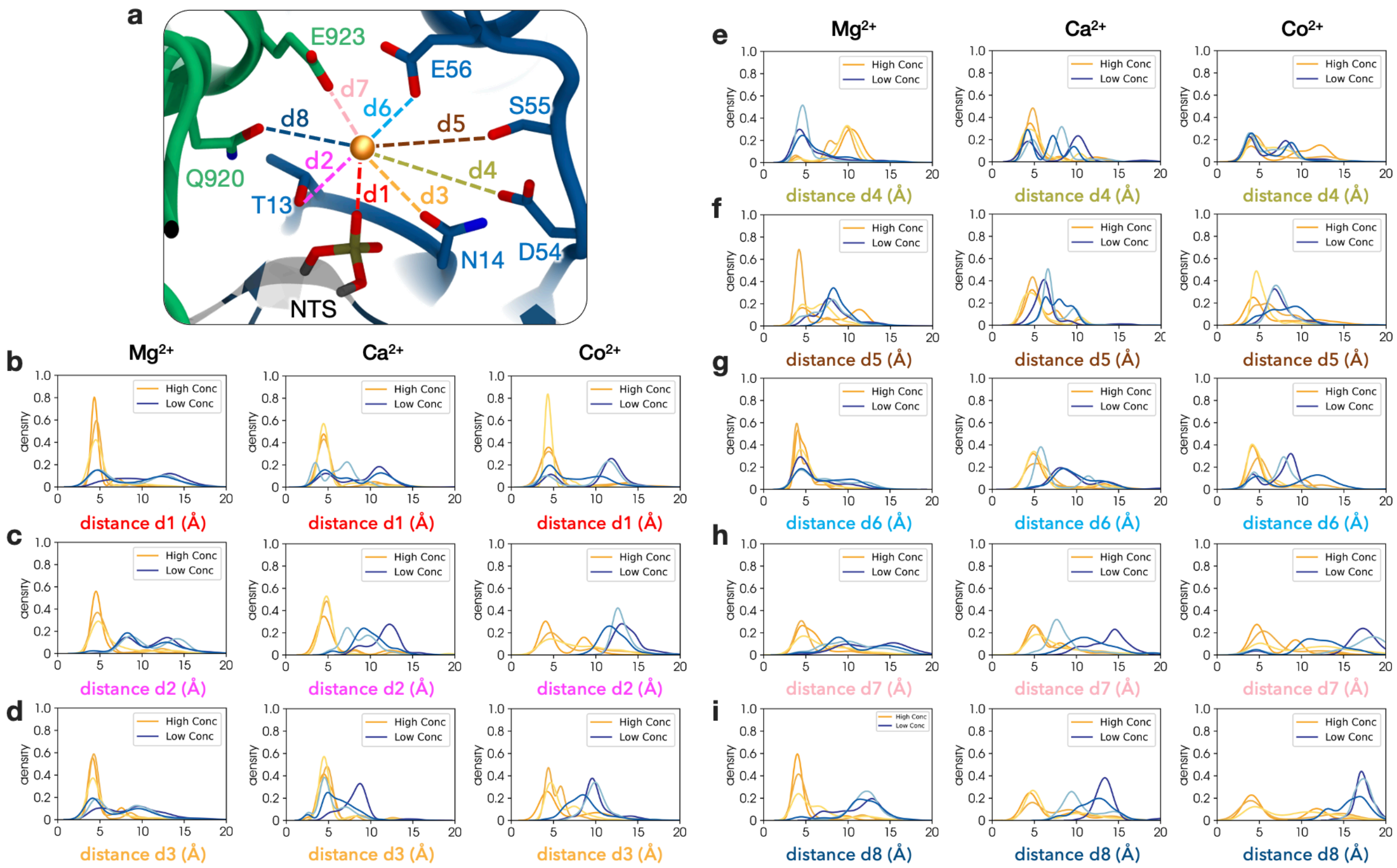

Supplementary Fig. 15. Analysis of the interactions established by divalent metal ions within the divalent metal binding pocket (DBP). a. Overview of the cryptic DBP formed during molecular dynamics simulations of the pre-active Cas9 (PDB 5F9R[1]). This pocket comprises residues of the RuvC domain and of the L2 region, as well as the DNA non-target strand (NTS), surrounding a divalent ion (Mg^{2+}, Ca^{2+}, or Co^{2+}). Key distances between the divalent ion and the surrounding DBP residues are highlighted (distances d1-d8). These distances connect the metal ion with the DNA NTS (atom P of nucleotide G-2, d1), and residues T13 (atom OG1, d2), N14 (atom OD1, d3), D54 (atom OD1, d4), S55 (atom OH, d5), E56 (atom OE1, d6), E923 (atom OE1, d7) and Q920 (ato atom OE1, d8). **b-i.** Probability distribution of the d1-d8 distances over MD simulations of the pre-active Cas9 (PDB 5F9R) at high (10 mM, yellow) and low (10 μM, blue) concentrations of divalent ions: Mg^{2+} (first column), Ca^{2+} (second column), and Co^{2+} (third column). Data are shown for three independent replicates of ~1 μs each, for each ionic concentration. This analysis indicates the formation of bridging interaction between the RuvC domain, the DNA NTS, and the L2 region. These interactions mainly form at high concentration of divalent ions and are consistent for all three divalent ions under investigation. The occurrence of these bridging interactions over the simulated ensemble is reported in Fig. 3d (main text) for Mg^{2+}, and Supplementary Fig. 17 (panels f) for Ca^{2+} and Co^{2+}.

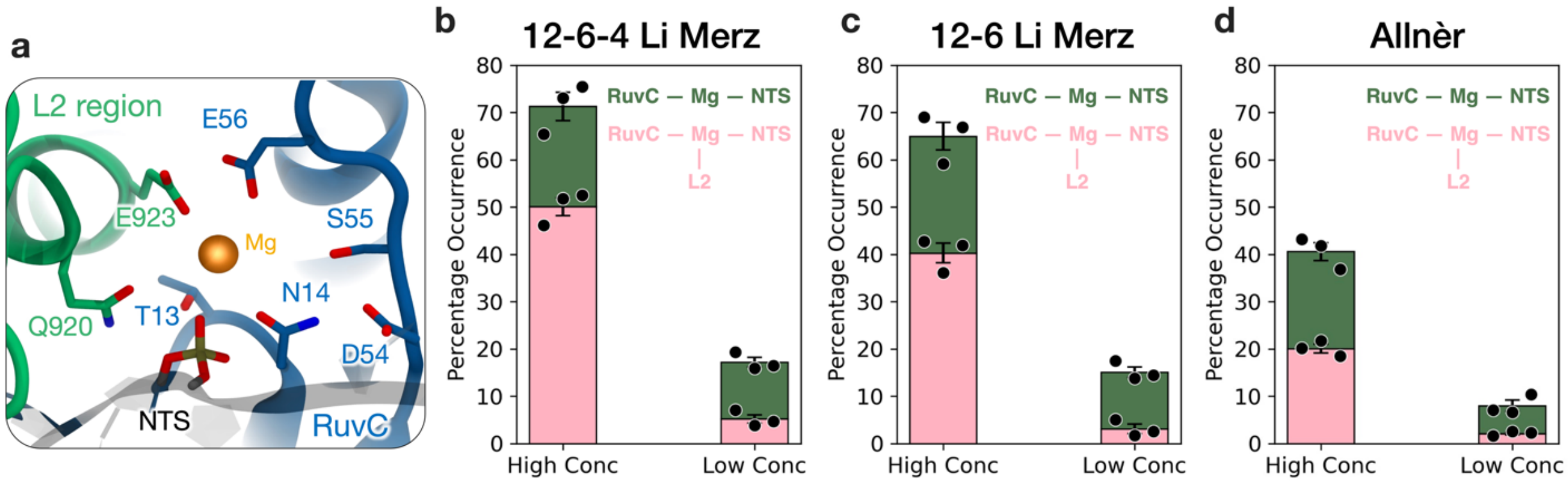

Supplementary Figure 16. Analysis of Mg^{2+} interactions within the divalent metal binding pocket (DBP) using different force field parameters. a. Overview of the cryptic DBP formed during molecular dynamics simulations of the pre-active Cas9 (PDB 5F9R[1]). **b–d.** Percentage occurrence of bridging interactions between Mg^{2+} and: (i) the RuvC domain and DNA NTS (green), and (ii) the RuvC domain, DNA NTS and L2 region (pink), at high (10 mM) and low (10 μM) Mg^{2+} concentrations. Interactions were identified using a 5 Å distance cutoff. Bars represent the mean ± s.e.m. across three independent ~1 μs MD simulation replicas (n = 3 independent simulations per condition). MD simulations were performed using three different force field parameter sets for Mg^{2+} ions: **b.** the 12-6-4 Li & Merz parameters, which incorporate both electrostatic and polarization effects. This model exhibits the highest percentage of bridging interactions, particularly at high Mg^{2+} concentrations, with a more pronounced distinction between high and low concentrations. **c.** the 12-6 Li & Merz parameters, a simplified version of the 12-6-4 model that primarily accounts for electrostatic interactions without polarization effects. This model yields bridging interactions comparable to those observed with the 12-6-4 polarizable model (panel b). **d.** the Allnér parameters, a widely used model for divalent cations that focuses primarily on nonbonded interactions. This model reports fewer bridging interactions compared to the Li & Merz models while maintaining similar trends. Mg^{2+} ions bind within the pockets, and an increased Mg^{2+} concentration enhances bridging interactions between the RuvC domain, the NTS, and the L2 region compared to low Mg^{2+} concentrations.

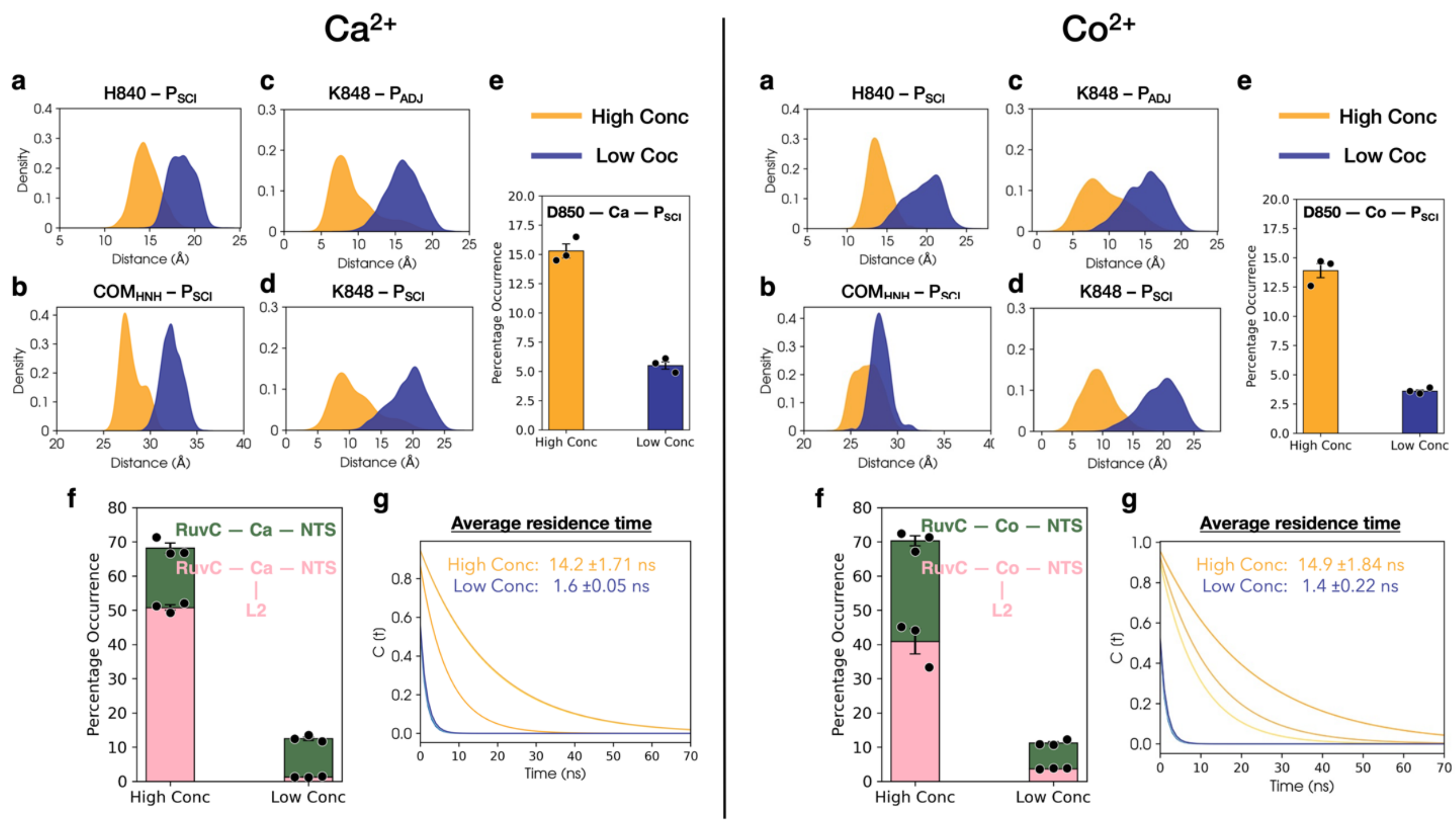

Supplementary Figure 17. Structural and functional analysis of divalent ion binding in Cas9, shown for Ca^{2+} (left) and Co^{2+} (right). a-d. Probability distribution of key distances bridging the HNH catalytic core and the DNA target strand (TS) over MD simulations of the pre-active Cas9 (PDB 5F9R[1]) at high (10 mM, yellow) and low (10 μM, blue) concentrations of divalent ions (Ca^{2+}, data on the left; Co^{2+}, data on the right). The distances measured include: the catalytic H840 and P_{SCI} (**a**), centre of mass (COM) of the HNH domain and P_{SCI} (**b**), K848 and P_{SCI} (**c**), K848 and the (P_{ADJ}) phosphate adjacent to P_{SCI} (**d**). Data from the aggregate ensemble of three simulation replicates (data from the individual simulation replicates are in Supplementary Fig. 10). **e.** Occurrence of metal (Ca^{2+}/Co^{2+}) interactions within the HNH active site. Percentage occurrence (mean ± s.e.m, n=3) of bridging interactions between the metal, D850 (HNH) and P_{SCI} at high and low ionic concentrations. **f.** Occurrence of metal (Ca^{2+}/Co^{2+}) interactions within the divalent metal binding pocket. Percentage occurrence (mean ± s.e.m, n=3) of bridging interactions between the metal and: (i) the RuvC domain and the DNA NTS (green), and (ii) the RuvC domain, DNA NTS, and the L2 region (pink) at high and low ionic concentrations. **g.** Average residence time (mean ± s.e.m, n=3) of metal ions (Ca^{2+}/Co^{2+}) calculated from the time correlation function (C_t), showing longer residence times at high concentrations compared to low concentrations of divalent ions. The same analysis was conducted in the presence of Mg^{2+}, with results presented in Fig. 3 (main text). Overall, higher concentrations of divalent ions promote the formation of bridging interactions, which in turn facilitate the transition of the HNH domain to its active state.

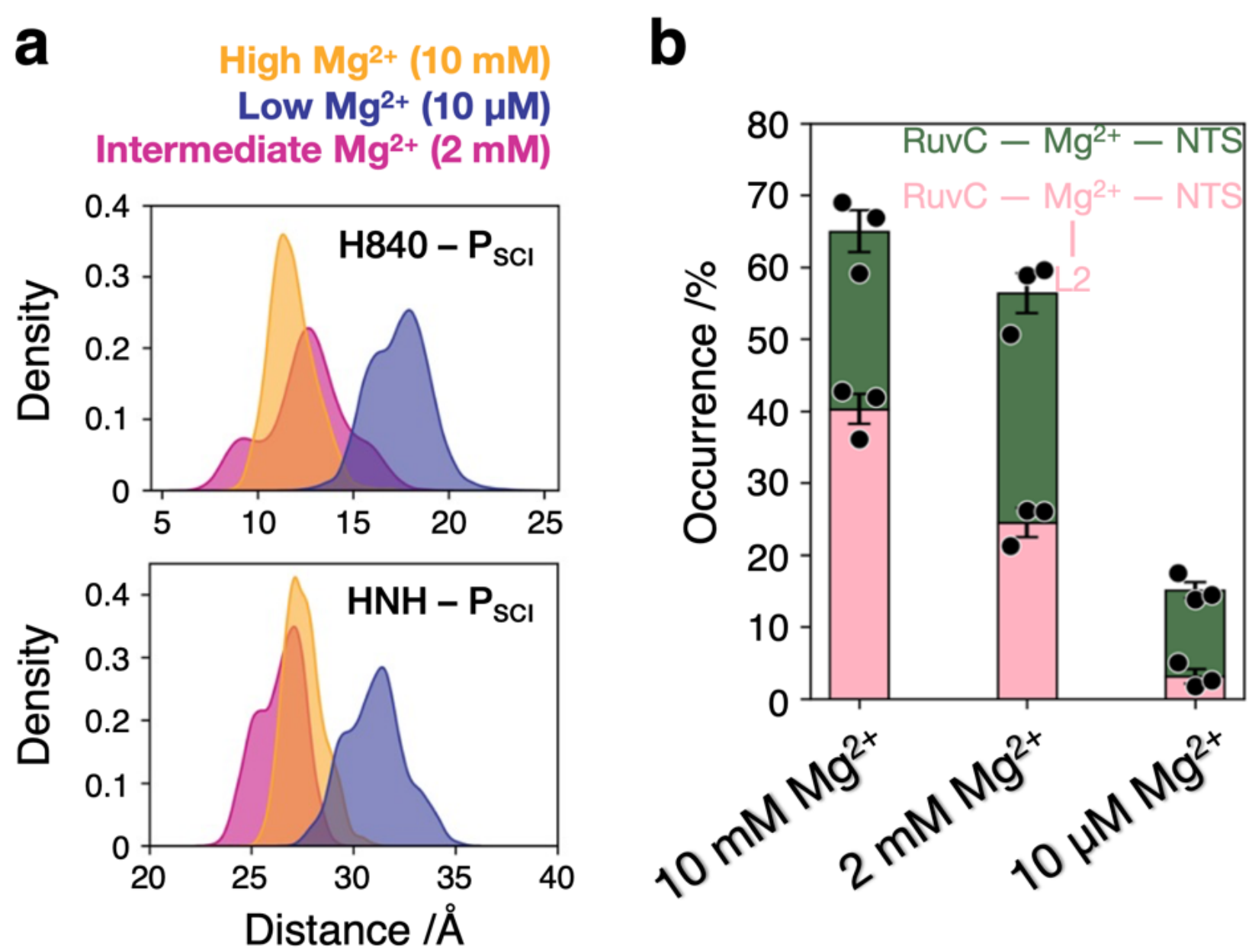

Supplementary Figure 18. Comparison of Mg^{2+}-mediated interactions at high, low, and intermediate Mg^{2+} concentrations. a. Probability distributions of key distances bridging the HNH catalytic core and the DNA target strand at high (10 mM, yellow), low (10 μM, blue) and intermediate (2 mM, magenta) Mg^{2+} concentrations ([Mg^{2+}]). Distances include those between residue H840 (Cα atom) and the scissile phosphate (P_{SCI}) (top), as well as the distance between the centre of mass of HNH and P_{SCI} (bottom). **b.** Percentage occurrence (mean ± s.e.m, n=3) of Mg^{2+}-mediated bridging interactions within the divalent metal binding pocket (DBP) at high and low [Mg^{2+}]. Within the DBP, Mg^{2+} interacts with the RuvC domain and the non-target strand (NTS) (green), while simultaneously bridging the L2 region (pink). Data were computed over the full simulation ensemble.

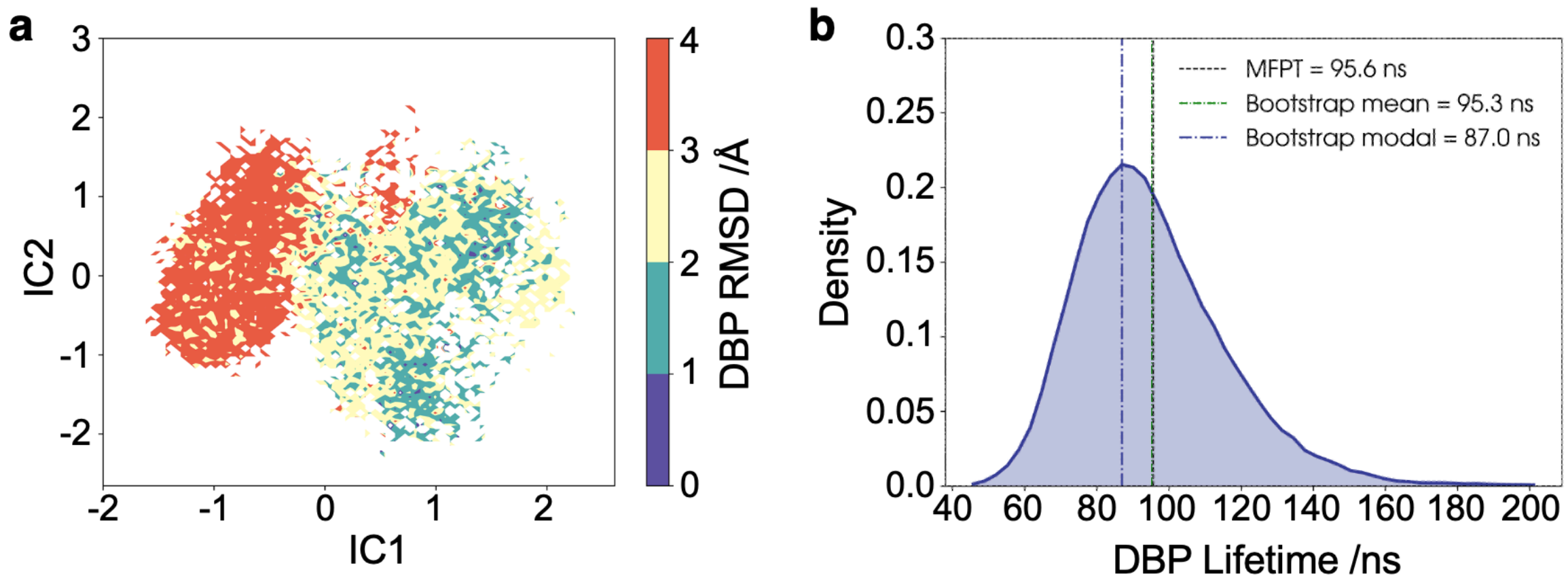

Supplementary Figure 19. Markov state model (MSM)-based estimation of the structural lifetime of the cryptic divalent binding pocket (DBP). a. Projection of the cryptic pocket root-mean-square deviation (RMSD) onto the time-lagged independent component analysis (tICA) landscape derived from MD simulations of the pre-active Cas9 state (PDB: 5F9R)[1] under high Mg^{2+} concentration conditions. The pocket RMSD was calculated using all heavy atoms of the pocket-lining residues and mapped onto the first two time-lagged independent components. Lower RMSD values indicate retention of a native-like DBP geometry, whereas higher RMSD values indicate geometric distortion or disruption of the pocket architecture. **b.** Bootstrap distribution of the MSM-derived DBP lifetime. The pocket lifetime was estimated as the mean first passage time (MFPT) from the pocket-containing ensemble, defined by metastable states 0 and 1, to the disrupted-pocket ensemble, defined by metastable states 2 and 3. The distribution was obtained by block bootstrapping the metastable-state trajectory and recalculating the MFPT for each bootstrap replicate. The resulting distribution supports an ensemble-level DBP lifetime on the order of ~100 ns, consistent with the transient behaviour generally associated with cryptic pockets. Details in Supplementary Methods.

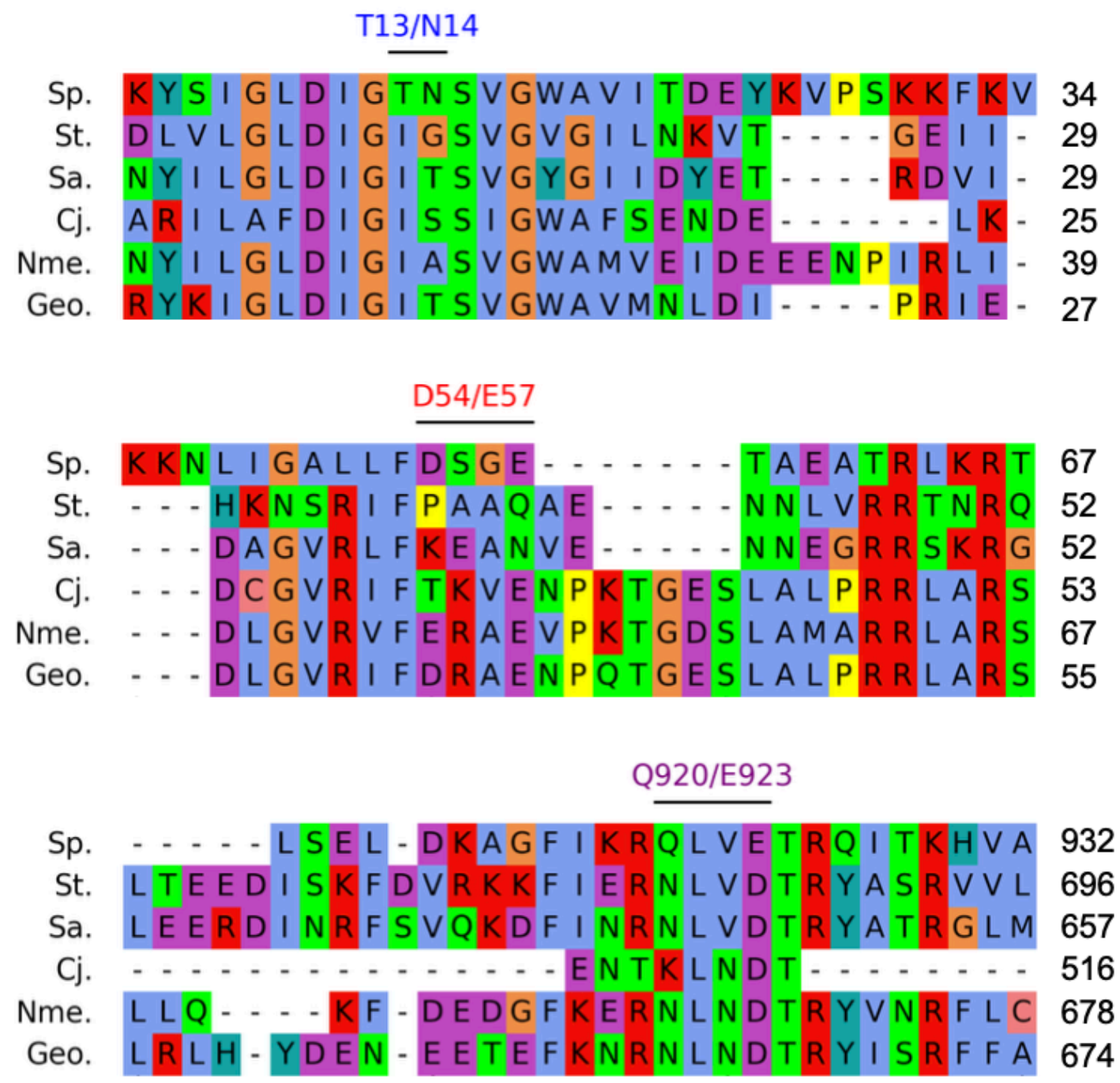

Supplementary Figure 20. Multiple sequence alignment of Cas9 orthologs in regions defining the divalent binding pocket (DBP). Multiple sequence alignment (MSA) of *Streptococcus pyogenes* Cas9 (SpCas9) with representative Cas9 orthologs, including *Streptococcus thermophilus* (StCas9), *Staphylococcus aureus* (SaCas9), *Campylobacter jejuni* (CjCas9), *Neisseria meningitidis* (NmeCas9), and *Geobacillus stearothermophilus* (GeoCas9). Sequence regions corresponding to residues involved in the divalent binding pocket (DBP) in SpCas9 are shown, including the T13/N14, D54/E57, and Q920/E923 regions. Residues mutated in the M1, M2, and M3 variants are indicated above the alignments. While strict sequence conservation is not observed across all positions, several acidic and polar residues are conserved or retain similar physicochemical properties, supporting preservation of a local environment compatible with divalent ion coordination and metal-dependent conformational regulation.

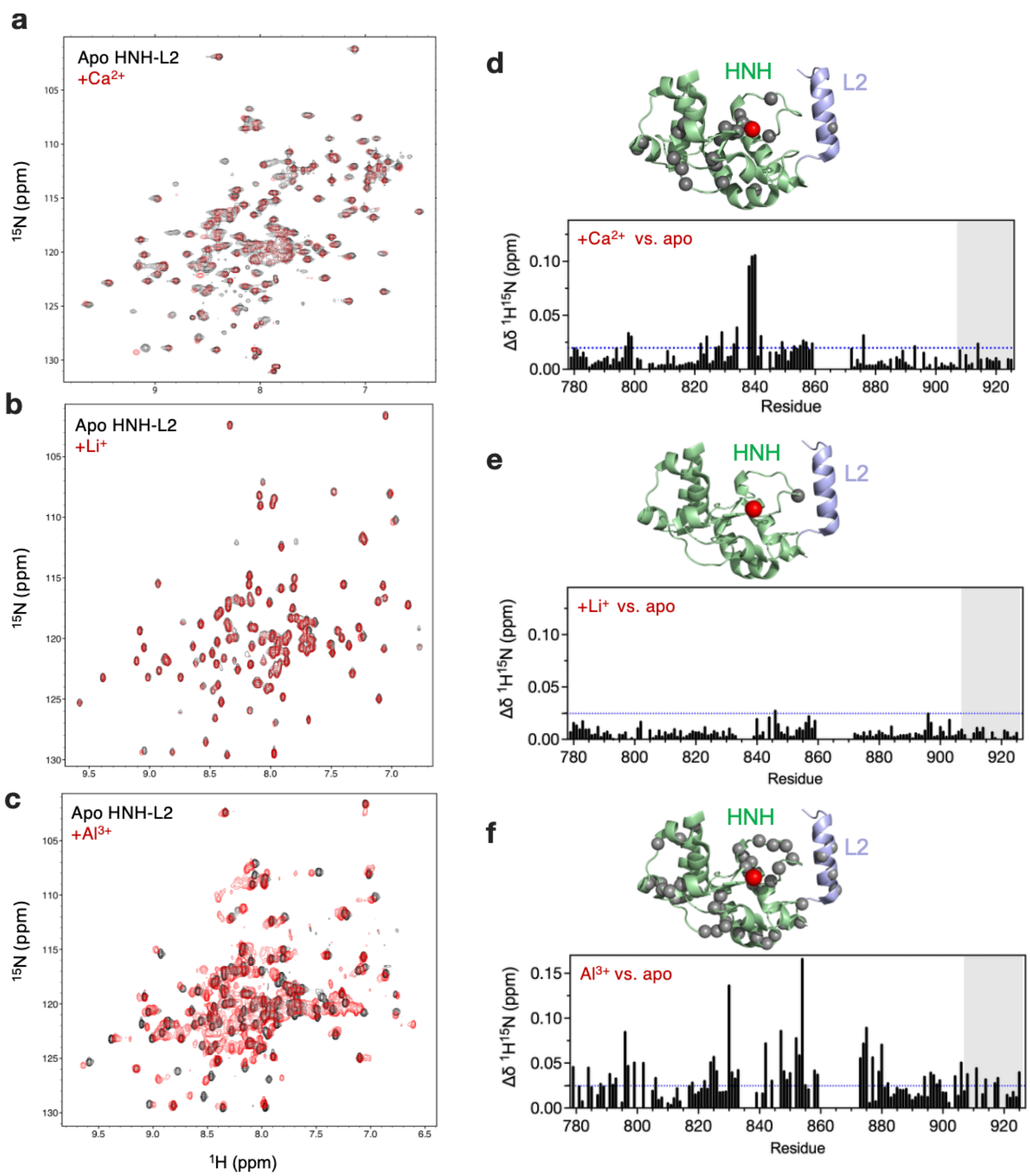

Supplementary Fig. 21. Structural Effects of Ca^{2+}, Li^{+} and Al^{3+} Binding to HNH-L2 by NMR. a-c. ^{1}H-^{15}N HSQC spectral overlays of apo HNH-L2 (grey) and HNH-L2 bound to Ca^{2+} (red, **a**), Li^{+} (red, **b**) and Al^{3+} (red, **c**). **d-f.** ^{1}H-^{15}N combined chemical shift perturbations (Δδ) for Ca^{2+}-bound HNH-L2 relative to apo (**d**), Li^{+}-bound HNH-L2 relative to apo (**e**) and Al^{3+}-bound HNH-L2 relative to apo (**f**). The grey area highlights L2 residues, the blue dashed line denotes 1.5σ above the 10% trimmed mean of all shifts. Chemical shift perturbations >1.5σ are mapped onto the structure of HNH-L2 (green, with L2 in blue) as grey spheres. The red sphere denotes a significant perturbation at the catalytic H840. Data for Mg^{2+} are reported in Fig. 4.

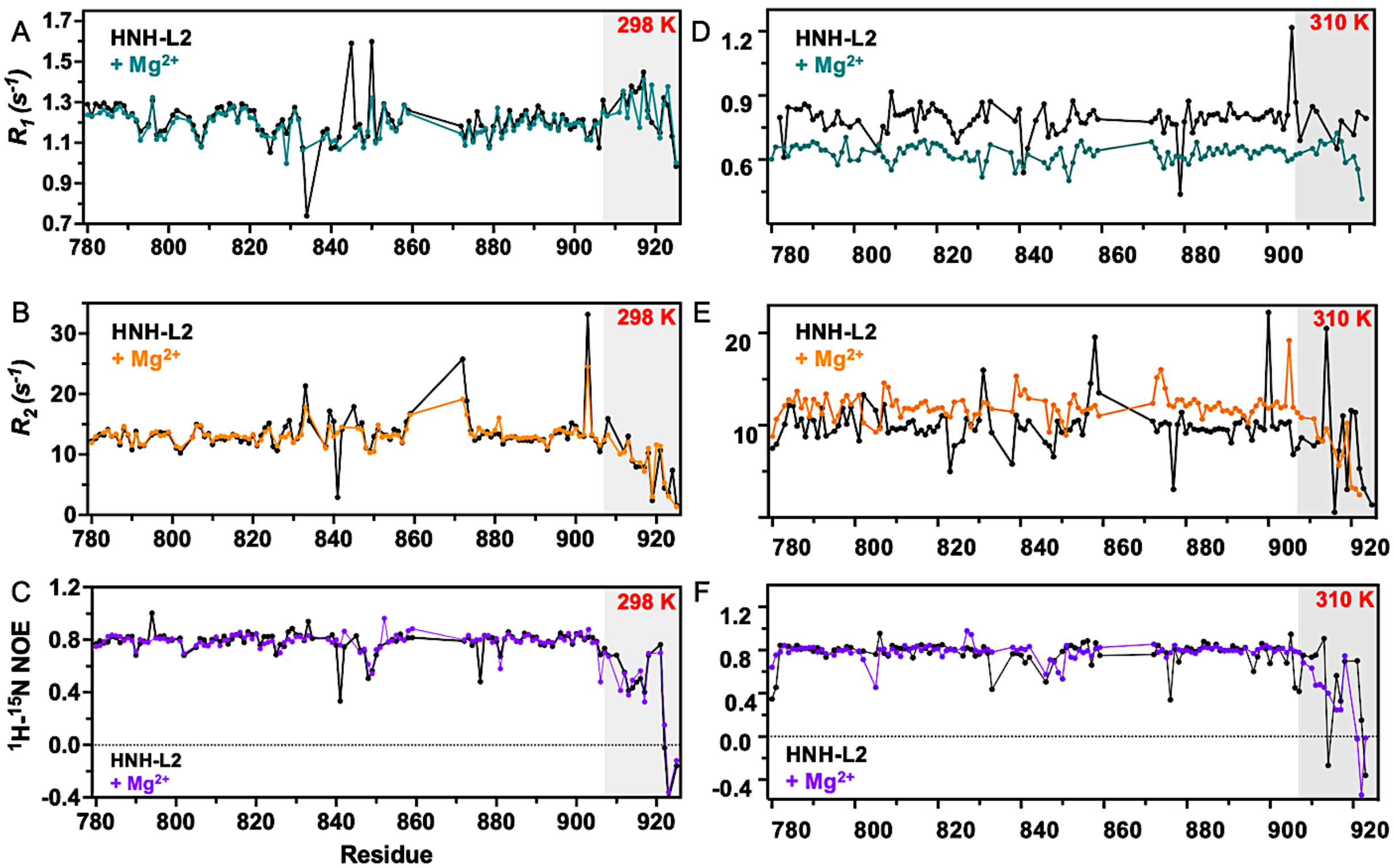

Supplementary Fig. 22. Dynamic Impacts of Mg^{2+} Binding to HNH-L2 by NMR. a-c. Fast timescale motions (ps – ns) assessed via longitudinal (R_1) and transverse (R_2) relaxation rates and ^{1}H-[^{15}N] heteronuclear NOEs for apo HNH-L2 (black) and Mg^{2+} bound HNH-L2 (teal, orange, and purple for R_1, R_2, and NOE, respectively). The grey area highlights L2 residues. Data were collected and analysed at two temperatures, 298 K and 310 K, encompassing the range of temperatures used in computational, biochemical, and biophysical experiments.

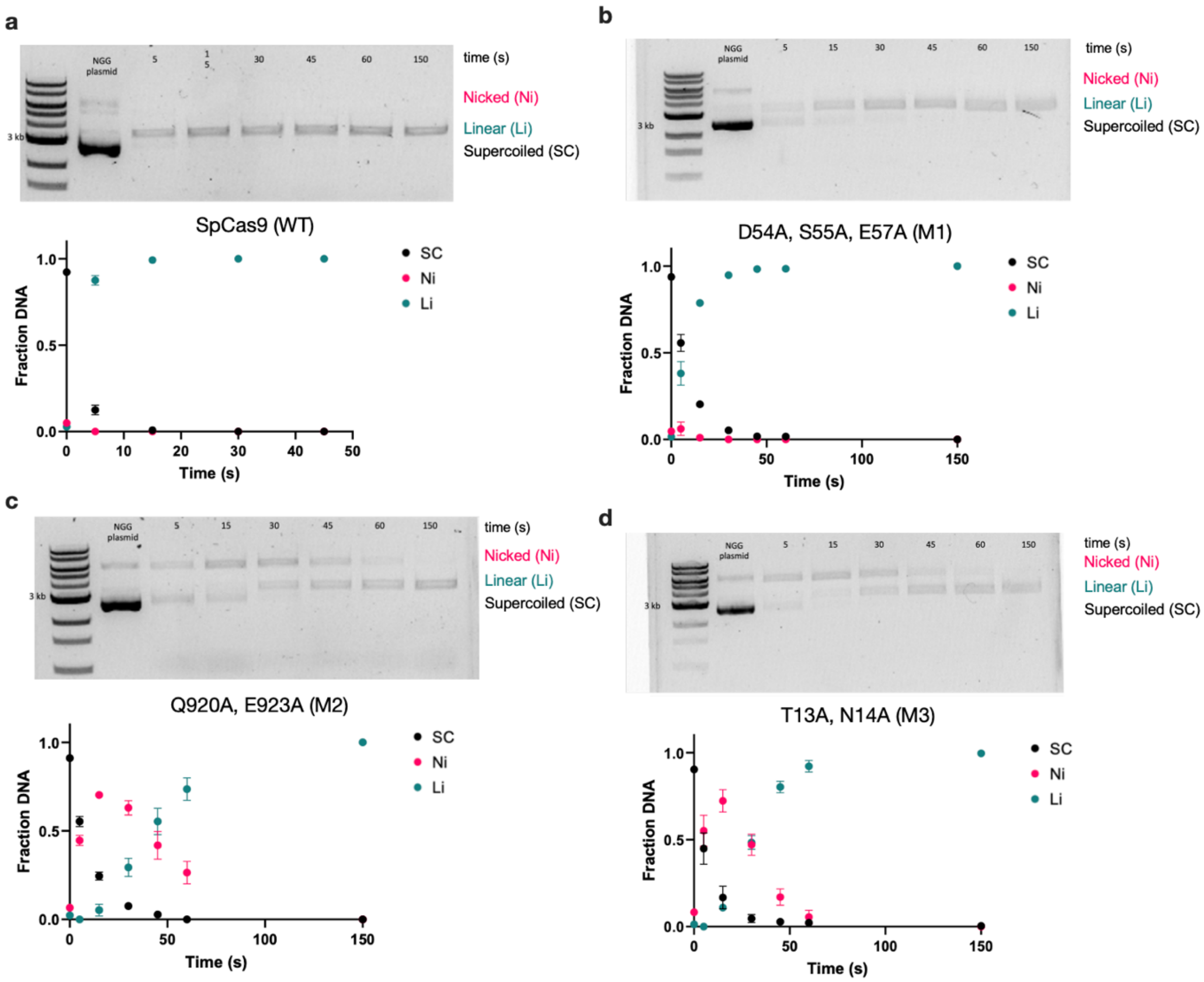

e

	WT		M1		M2		M3	
	Rate	SE	Rate	SE	Rate	SE	Rate	SE
**Disappearance of SC**	0.404995	0.0158961	0.106318	0.00435487	0.0904809	0.00343136	0.124783	0.0130058
**Appearance of Li**	0.404995	0.0158961	0.106	5.00E-07	0.0328934	0.0010782	0.051557	0.00108049

Supplementary Fig. 23. Plasmid DNA Cleavage Activity of SpCas9 and Mutants. Plasmid DNA cleavage assays were performed for wild-type SpCas9 (WT SpCas9) and mutants M1 (D54A, S55A, E57A), M2 (Q920A, E923A), and M3 (T13A, N14A). Reactions were monitored over time, quenched, and analysed by agarose gel electrophoresis. The DNA species – supercoiled (SC; substrate), nicked (Ni), and linear (Li; product) – are indicated on the gel images. For each protein variant, triplicate time courses were quantified and fit by non-linear regression using KinTek software: SC species to a single-exponential decay, Ni and Li species to double-exponential models. Quantified data (SC: black, Ni: pink, Li: green) are shown below each gel as mean ± standard error from three independent experiments. Panels: **a.** wild-type (WT) SpCas9, **b.** M1, **c.** M2, **d.** M3. Panel **e** shows the derived rate constants (s^{-1}, ± fit error) obtained from the kinetic fits, which were used for subsequent analysis.

a

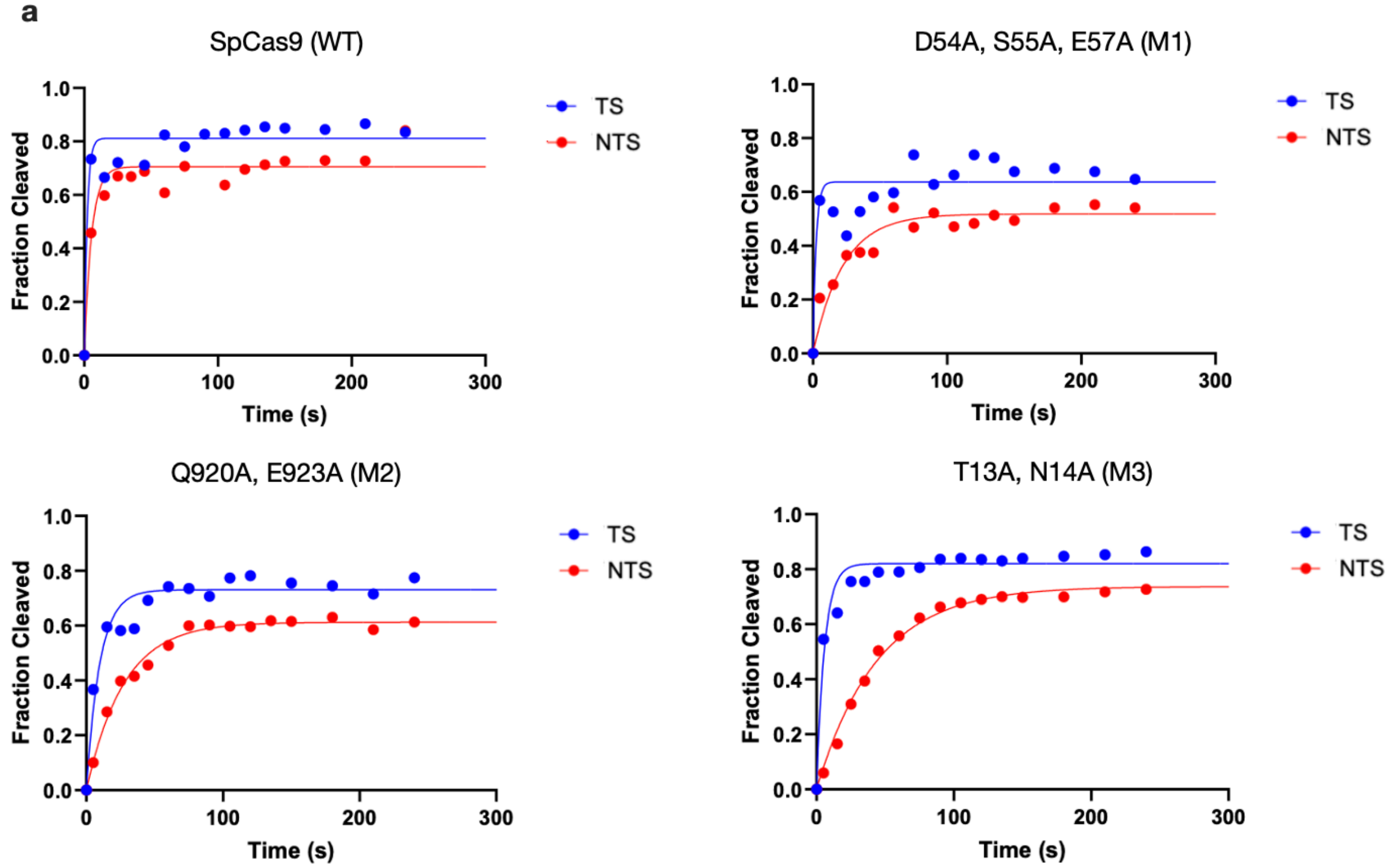

b

	WT		M1		M2		M3	
	Rate	SE	Rate	SE	Rate	SE	Rate	SE
**TS**	0.4585	0.162	0.432	0.2376	0.101	0.01561	0.1704	0.02603
**NTS**	0.1834	0.03918	0.04453	0.006242	0.03659	0.001939	0.02285	0.00102

Supplementary Fig. 24. Comparative Analysis of DNA Target Strand (TS) and Non-Target Strand (NTS) Cleavage Efficiency. a. Cleavage time courses for wild-type SpCas9 (WT) and mutants M1 (D54A, S55A, E57A), M2 (Q920A, E923A), and M3 (T13A, N14A) using fluorescently labelled oligoduplex DNA substrates under saturating conditions to measure the rates of TS and NTS cleavage. The fraction cleaved is plotted over time and fit to a single-exponential equation (solid lines) to obtain observed cleavage rates (k_{obs}). Data points represent experimental measurements for TS (red) and NTS (blue) cleavage. **b.** Summary of derived k_{obs} values (s^{-1}, ± fit error) for TS and NTS cleavage for each protein variant.

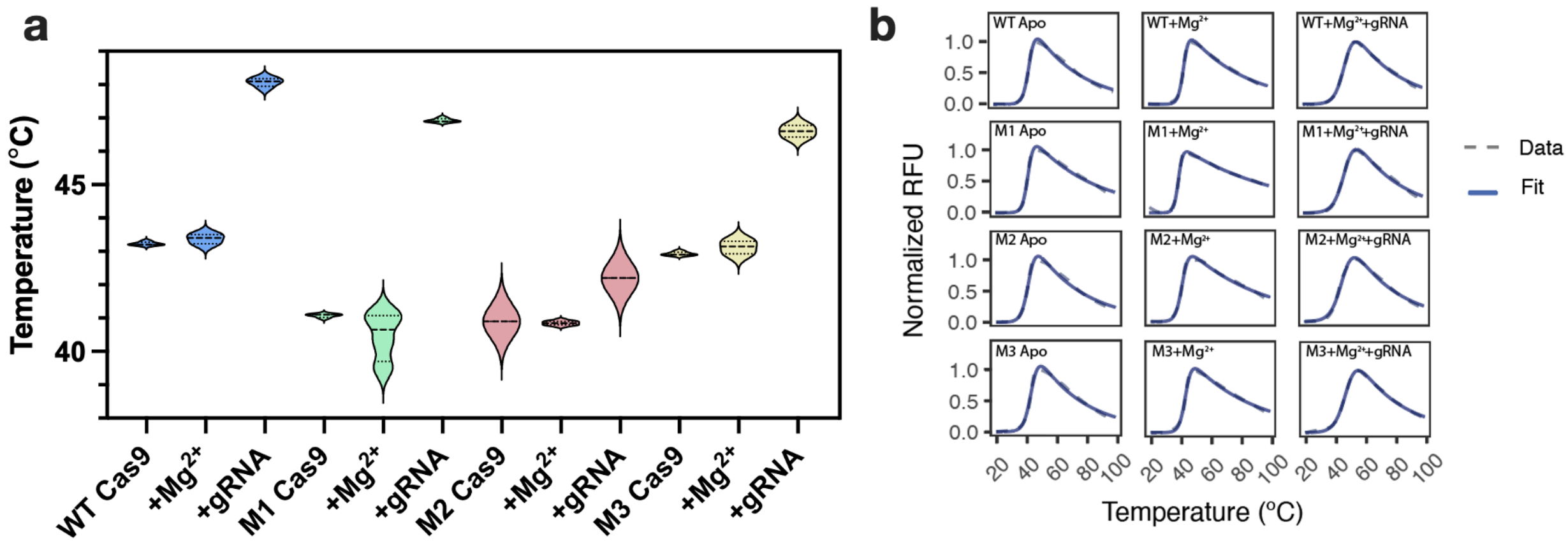

Supplementary Fig. 25. DSF-derived thermal stabilities of SpCas9 and its variants. a. Violin plots showing the distribution of melting transition temperatures (T_m) measured by differential scanning fluorimetry for WT Cas9 and the M1, M2, and M3 variants under apo, Mg^{2+}-bound, and Mg^{2+} and gRNA-bound conditions. Dotted horizontal lines indicate the median and quartiles for each condition. All variants exhibit melting temperatures comparable to WT Cas9, while guide RNA binding produces additional stabilization across all constructs. **b.** Representative raw DSF traces (dashed grey line) and corresponding sigmoidal fits (blue line) for each condition.

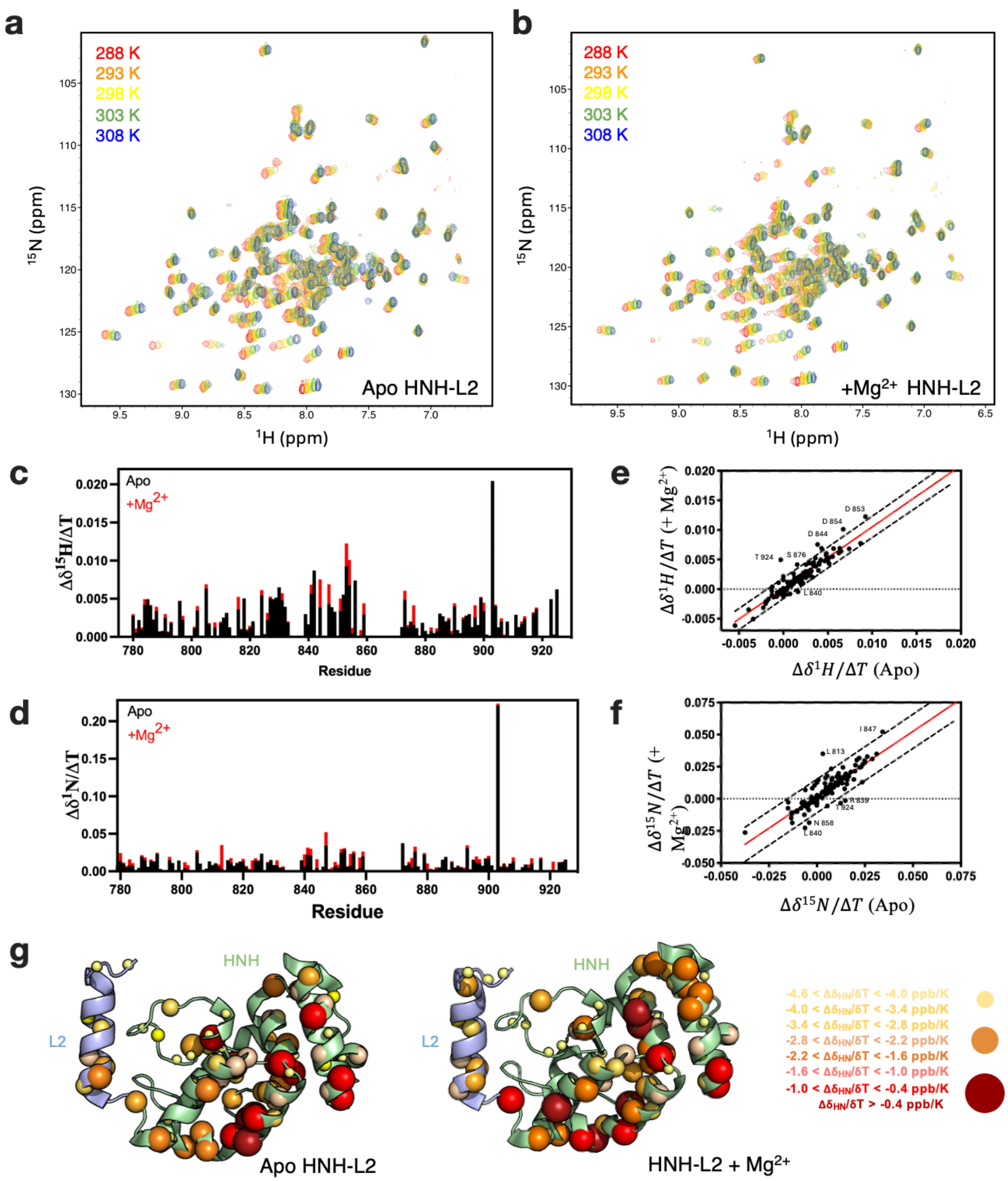

Supplementary Fig. 26. Temperature-dependent NMR characterization of the HNH–L2 construct in apo and Mg^{2+}-bound states. a-b. ^{1}H–^{15}N HSQC spectral overlays of the HNH–L2 construct acquired at temperatures ranging from 288 to 308 K in the absence (**a**) and presence (**b**) of Mg^{2+}. Spectra remain well dispersed across the temperature range, indicating preservation of the folded state under both conditions. **c-d.** Residue-specific temperature coefficients ($\Delta\delta^{1}H/\Delta T$ and $\Delta\delta^{15}N/\Delta T$) for the apo (black) and Mg^{2+}-bound (red) HNH–L2 construct. **e-f.** Correlation plots comparing residue-specific temperature coefficients measured for the apo and Mg^{2+}-bound states of HNH-L2 using ^{1}H **(e)** and ^{15}N **(f)** chemical shifts. Dashed black lines denote ±10% deviation

from the diagonal (red line). Residues outside these bounds exhibit the most significant changes to their ensemble over this temperature range **g.** Per-residue $\Delta\delta^{1}H/\Delta T$ temperature coefficients are mapped onto the structure of the HNH-L2 (green with L2 in blue) for the apo (left) and Mg^{2+}-bound (right) states. Sphere colour and size reflect the magnitude of the temperature coefficient. Overall, the data indicate preservation of the folded HNH–L2 ensemble, similar hydrogen bonding patterns, and comparable structural compressibility in both apo and Mg^{2+}-bound conditions across this temperature range.

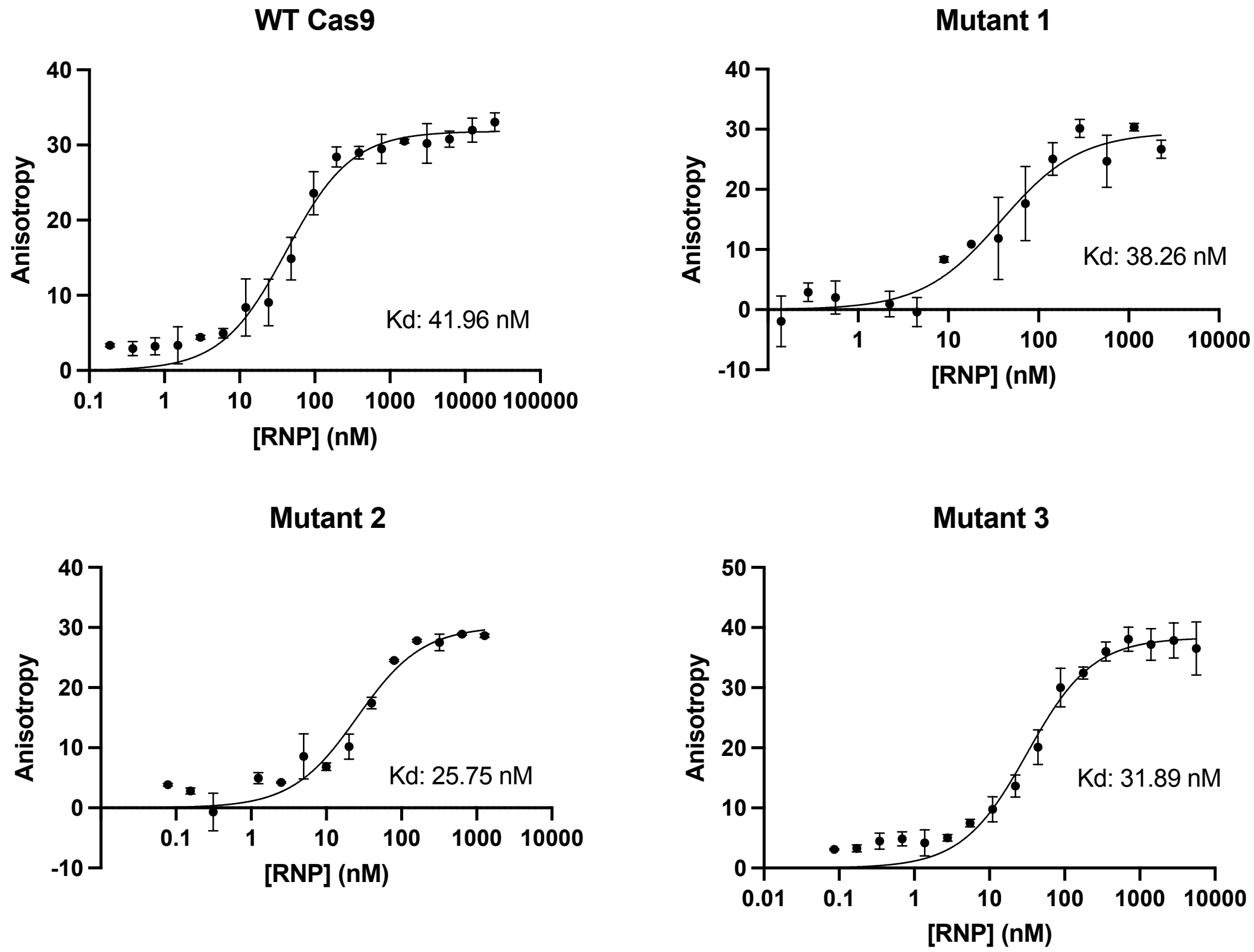

Supplementary Fig. 27. Cas9 mutants show similar binding affinities to target DNA. a-d. Fluorescence anisotropy binding assays used to determine apparent dissociation (K_d) constants for wild-type Cas9 (**a**), M1 (**b**), M2 (**c**) and M3 (**d**) variants binding target dsDNA. Cas9 proteins were pre-complexed with sgRNA prior to incubation with FAM-labelled target DNA. Increasing concentrations of ribonucleoprotein (RNP) complexes were incubated with 10 nM of FAM-labelled target dsDNA, and fluorescence anisotropy was measured following equilibration (10-minute incubation time at 37°C). Anisotropy values were plotted as a function of RNP concentration (nM) and fit using a one-site specific binding model to determine apparent dissociation constants. Data represent the mean ± SD from three biological replicates. Error bars indicate standard deviation.

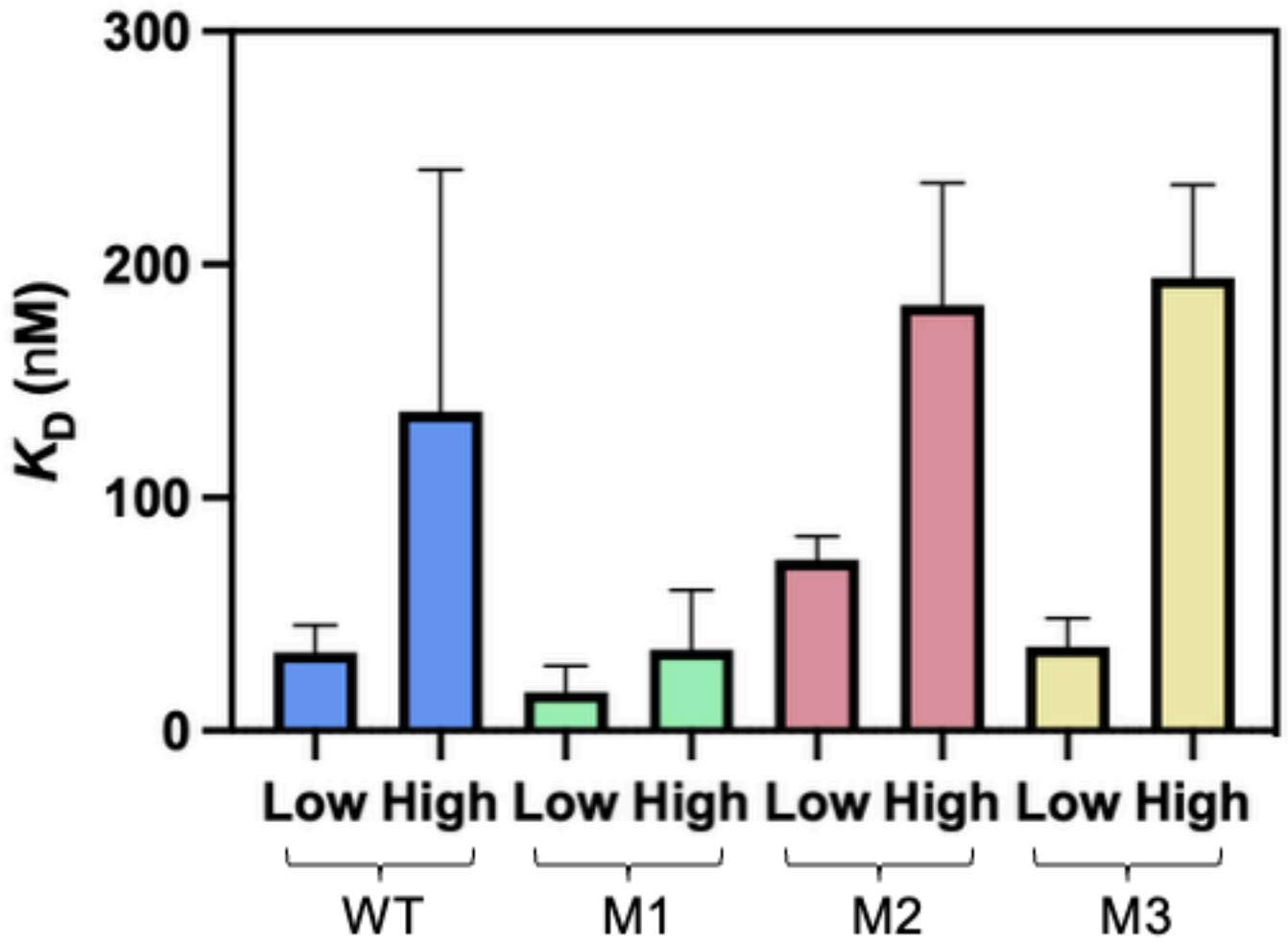

Supplementary Figure 28. MST-derived K_d values of WT Cas9, M1, M2, and M3 variants for gRNA in the presence of low (10 μM) and high (10 mM) Mg^{2+}. Values represent the mean ± s.d. from three technical replicates (n = 3 technical replicates per condition).

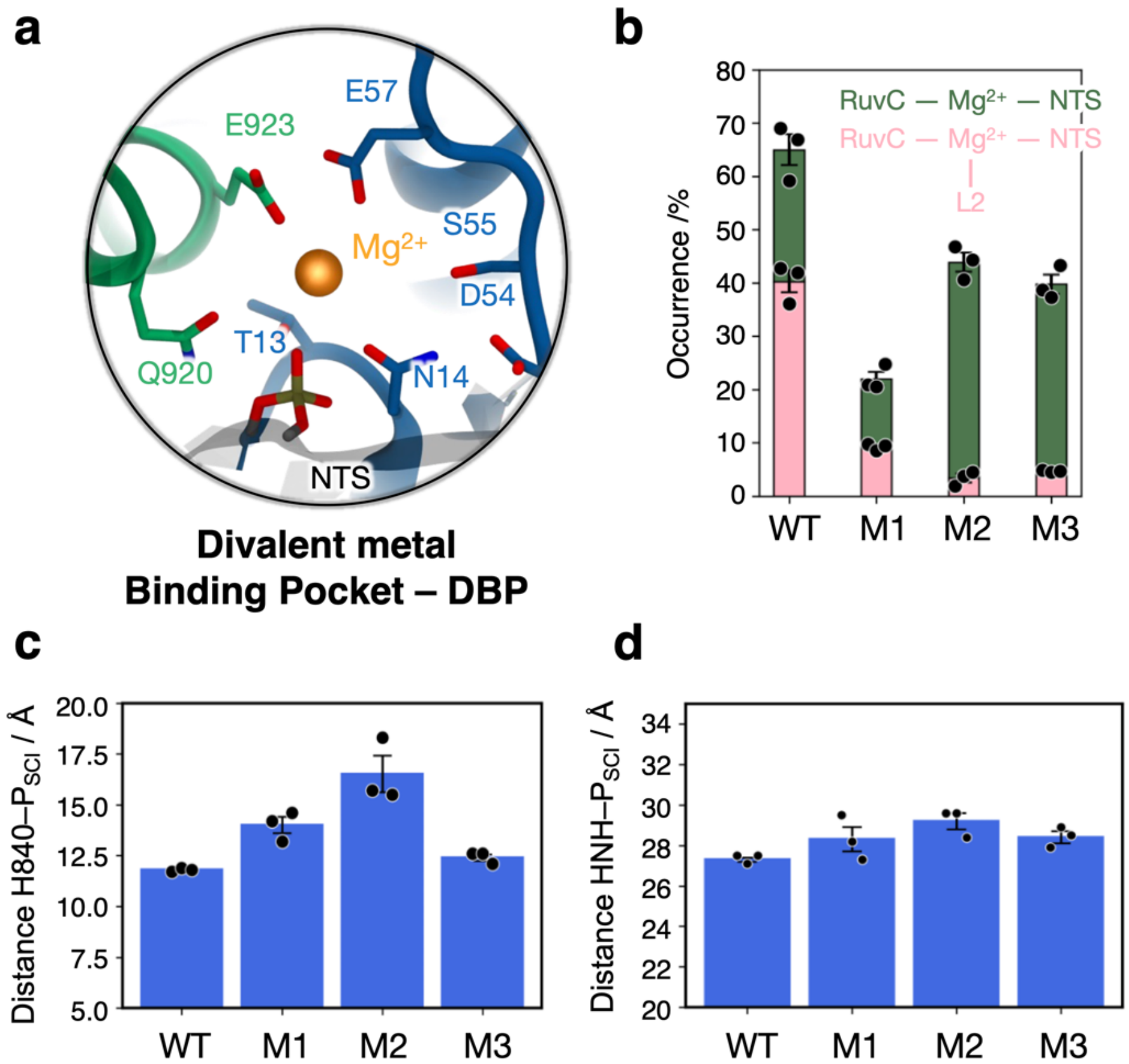

Supplementary Fig. 29. Effects of mutations on Mg^{2+}-mediated interactions and HNH positioning. a. Divalent binding pocket (DBP) identified in molecular dynamics (MD) simulations. The DBP is formed by residues from the RuvC domain, the L2 region, and the DNA non-target strand (NTS). The Mg^{2+} ion bridges interactions among these structural elements. Residues targeted by the M1 (D54A/S55A/E57A), M2 (Q920A/E923A), and M3 (T13A/N14A) mutations are shown. **b.** Percentage occurrence of Mg^{2+}-mediated bridging interactions within the DBP for the wild-type (WT) Cas9 and the M1, M2, and M3 variants under high Mg^{2+} concentration conditions (10 mM). Bridging interactions are categorized as Mg^{2+} simultaneously coordinating the RuvC domain and the NTS (green), or the RuvC domain, the NTS, and the L2 region (pink). Interactions were identified using a 5 Å distance cutoff. **c-d.** Mean distances between the catalytic residue H840 and the scissile phosphate (P_{SCI}) (**c**), and between the centre of mass of the HNH domain and P_{SCI} (**d**), reporting on catalytic positioning and global HNH domain positioning, respectively, in WT, M1, M2, and M3 systems. For panels **b–d**, values were computed from MD simulations of the pre-active Cas9 structure (PDB: 5F9R)[1] under high Mg^{2+} concentration conditions (10 mM), using three independent ~1 μs replicas per system (n=3). Error bars represent the standard error of the mean across replicas.

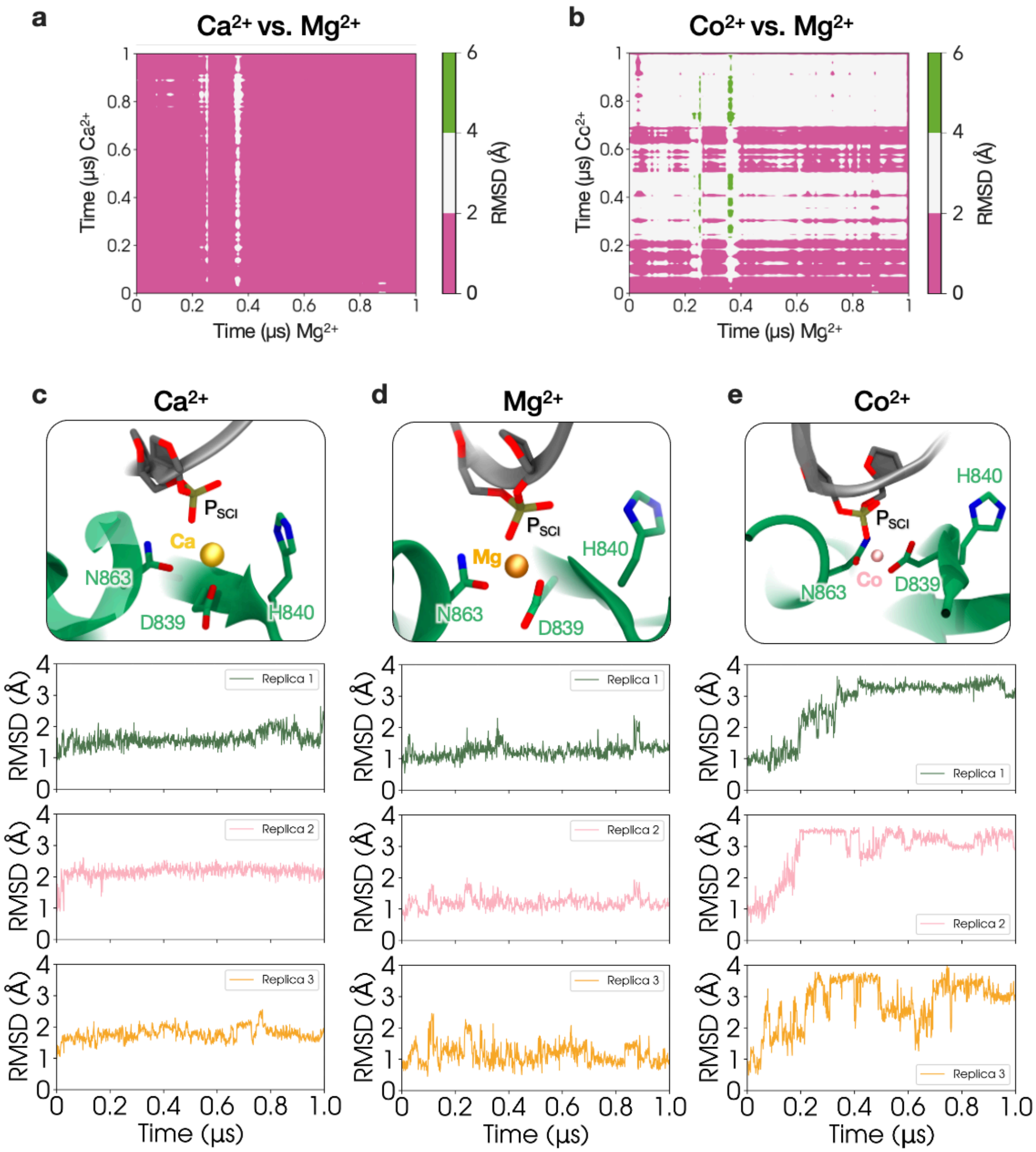

Supplementary Fig. 30. Structural analysis of the HNH active site in the presence of Ca^{2+}, Mg^{2+}, and Co^{2+}. a-b. Two-dimensional root-mean-square deviation (2D RMSD) plots comparing the HNH active site conformations from MD simulations of the active Cas9 (PDB: 6O0Y[2]) in the presence of Ca^{2+} vs. Mg^{2+} (**a**), as well as Co^{2+} vs. Mg^{2+} (**b**). RMSD calculations were performed considering all the heavy atoms of the active site residues and nucleobases within ~5 Å of the metal ion. The X-axis corresponds to the simulation time with Mg^{2+} at the catalytic centre, while the Y-axis corresponds to the simulation time with Ca^{2+} or Co^{2+}. **c-e.** Structural representations of the HNH active site (top panels) and plots of the active site's heavy atoms RMSD (bottom panels) in the presence of Ca^{2+} (**c**), Mg^{2+} (**d**), and Co^{2+} (**e**). For each system, data are computed from three independent ~1 µs long simulations (replica 1, 2, and 3).

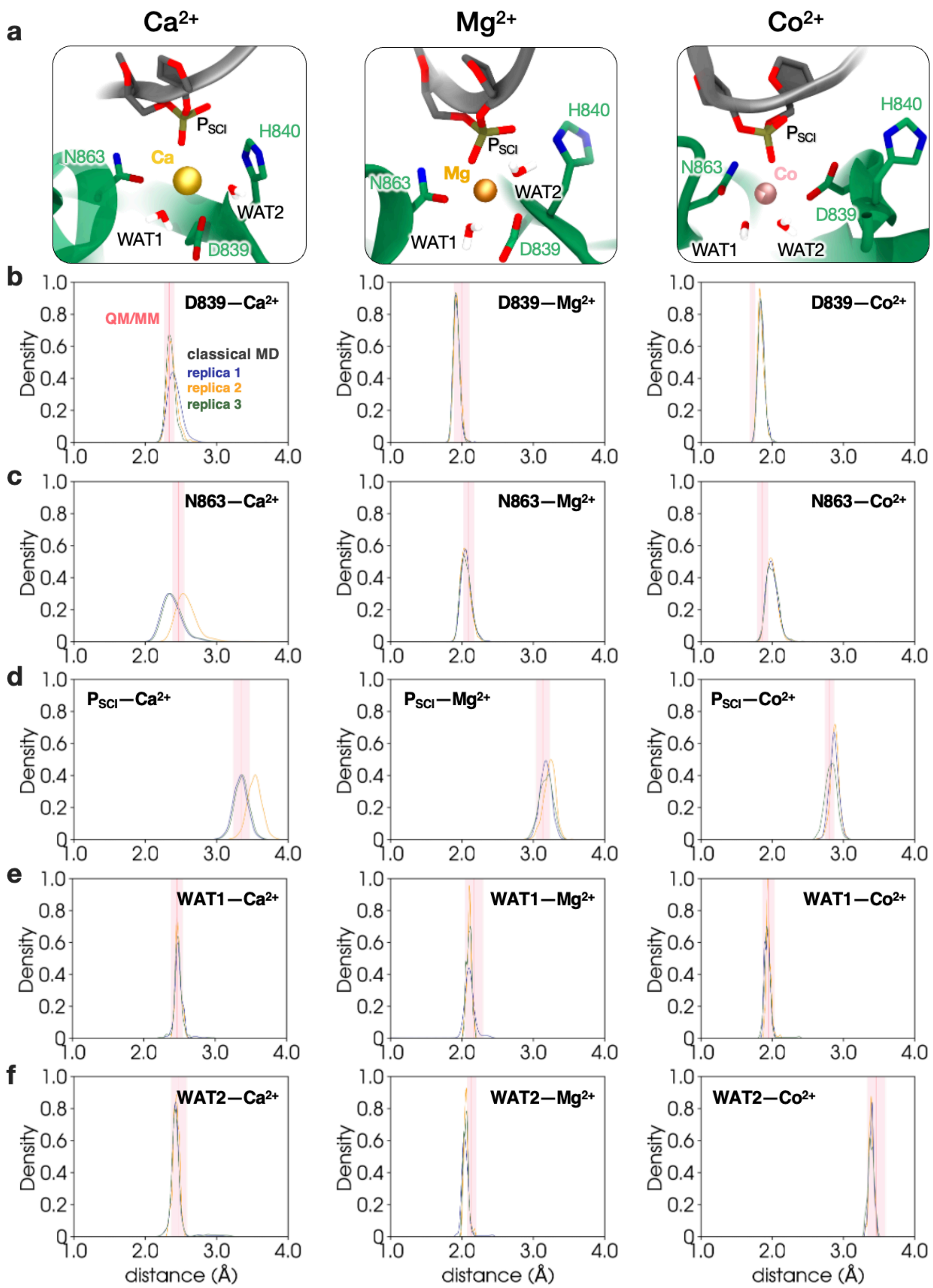

Supplementary Fig. 31. Metal coordination distances within the HNH active site in the presence of Ca^{2+}, Mg^{2+}, and Co^{2+}: comparison from classical MD and QM/MM simulations. **a.** Structural representations of the HNH active site (top panels) in the presence of Ca^{2+} (left), Mg^{2+} (middle), and Co^{2+} (right). Key active site residues and water molecules (WAT1 and WAT2) are highlighted, showing the coordination with the metal ions. **b-f.** Distributions of metal

coordination distances for Ca^{2+} (left), Mg^{2+} (middle), and Co^{2+} (right) obtained from classical MD simulations across three replicates. The red line in each plot denotes the average distance derived from QM/MM simulations, while the shaded region represents the standard deviation. **b.** Distance between D839 (atom OD2) and the metal ion. **c.** Distance between N863 (atom OD1) and the metal ion. **d.** Distance between P_{SCI} and the metal ion. **e.** Distance between WAT1 and the metal ion. **f.** Distance between WAT2 and the metal ion. The differences in coordination geometry between Ca^{2+}, Mg^{2+}, and Co^{2+} highlight the distinct effects these metal ions have on the geometry of the active site. Specifically, Ca^{2+} induces larger coordination distances, while Co^{2+} results in shorter coordination distances compared to Mg^{2+}. The QM/MM simulation distances serve as a reference, demonstrating that classical MD simulations align with the more detailed QM/MM results. This alignment indicates that classical MD simulations can replicate the coordination structures observed in QM/MM simulations.

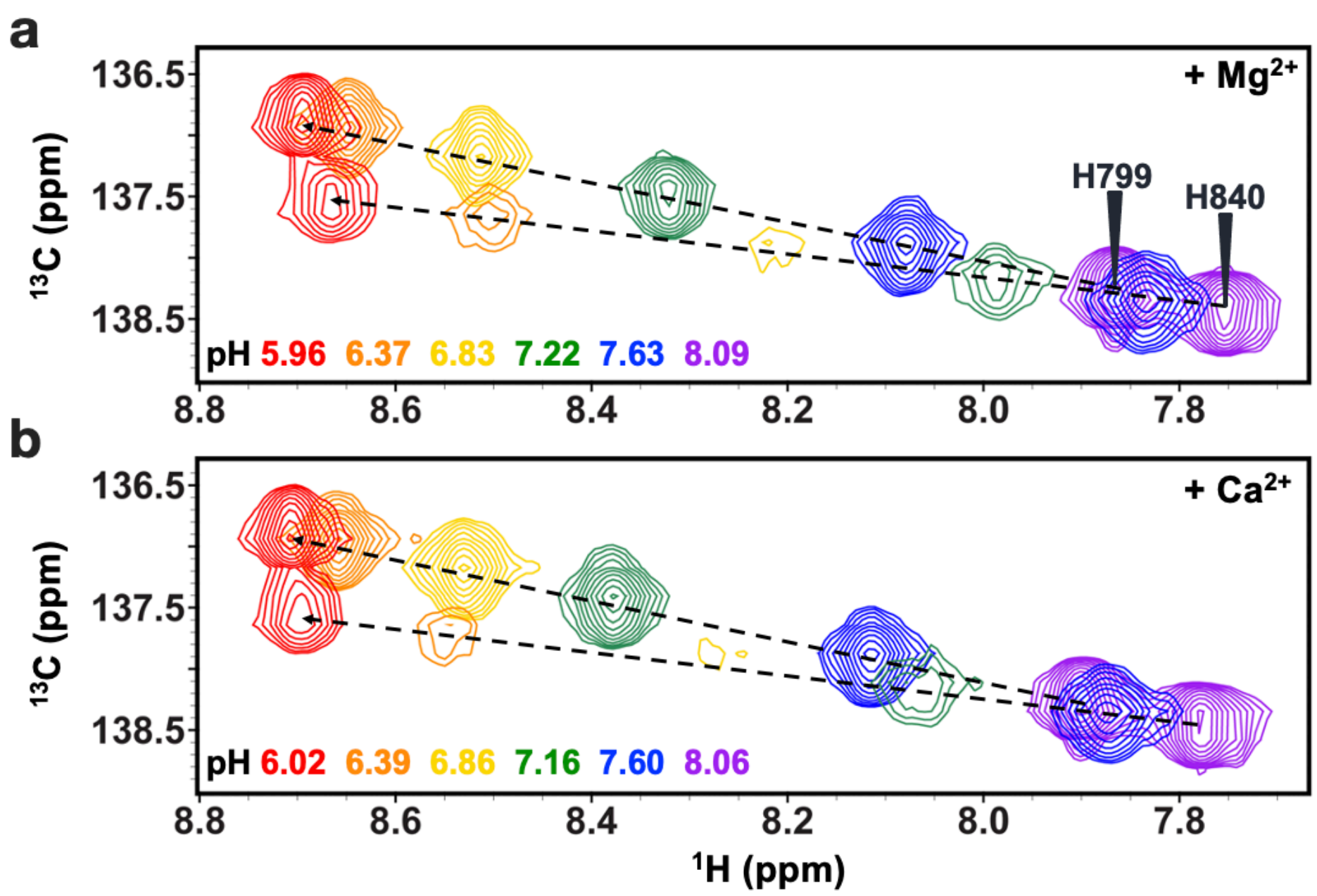

Supplementary Fig. 32. NMR pH Titration for pK_a Determination of the Catalytic H840 in the Presence of Metal Ions. pH titrations of the histidine side chains in HNH-L2 in the presence of Mg^{2+} and Ca^{2+} are shown in **a** and **b**, respectively, where the ^{1}H-$^{13}C_{\varepsilon 1}$ correlation changes as the histidine nitrogen atoms change protonation state. Resonances are coloured according to the pH values, and the chemical shift trajectories for H799 (upper resonances) and H840 (lower resonances) are indicated by dashed arrows.

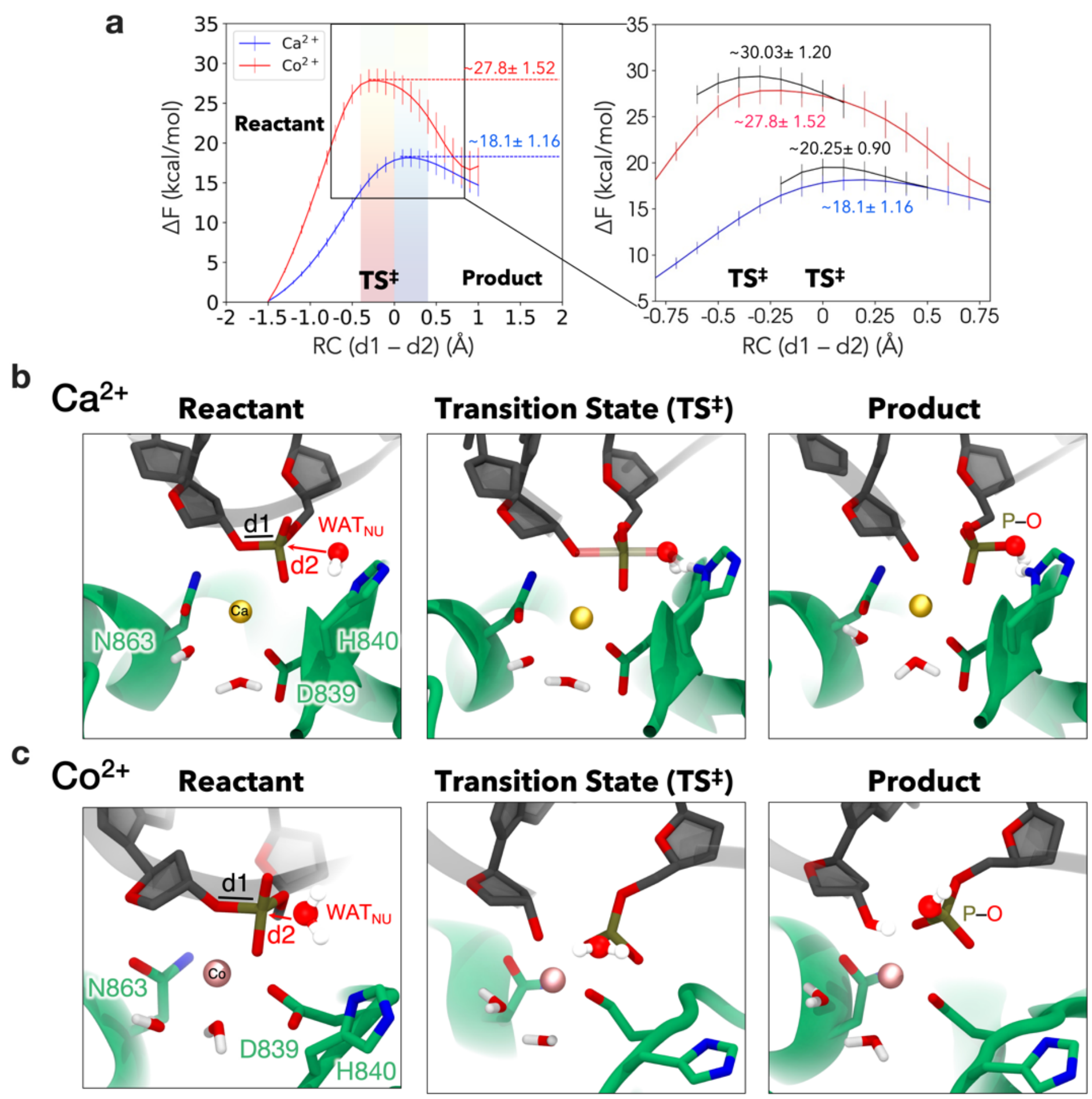

Supplementary Fig. 33. Catalytic mechanism of target strand cleavage in the presence of Ca^{2+} and Co^{2+}. a. Free energy profiles for phosphodiester bond cleavage in the presence of Ca^{2+} (blue) and Co^{2+} (red), computed through *ab-initio* QM/MM MD and a Thermodynamic Integration approach. The free energy profiles are computed along a reaction coordinate (RC) defined as the difference in distances between the breaking (d1, between P_{SCI} and O3') and the forming (d2, between the WAT_{nu} and P_{SCI}) P–O bonds, expressed as d1-d2. This RC is illustrated in the reactant state depicted in panels **b** and **c**. The free energy profiles evolve from the Reactants to Products passing through the Transition State (TS‡, region indicated using a red vertical shaded bar). A close-up view reports the forward and backward free energy profiles (black). Error bars show standard deviations obtained from error propagation analysis of the primary data set, with each data point representing the mean from the last ~6 to ~10 ps of converged *ab-initio* MD. The Helmholtz free energy is ~18.1 ± 1.16 kcal/mol in the presence of Ca^{2+}, and ~27.8 ± 1.52 kcal/mol in the presence of Co^{2+} (see Supplementary Methods). **b-c.** Representative snapshots illustrating key stages of the chemical step, including the Reactants, Transition States (TS‡) and Products in the presence of Ca^{2+} (**b**) and Co^{2+} (**c**).

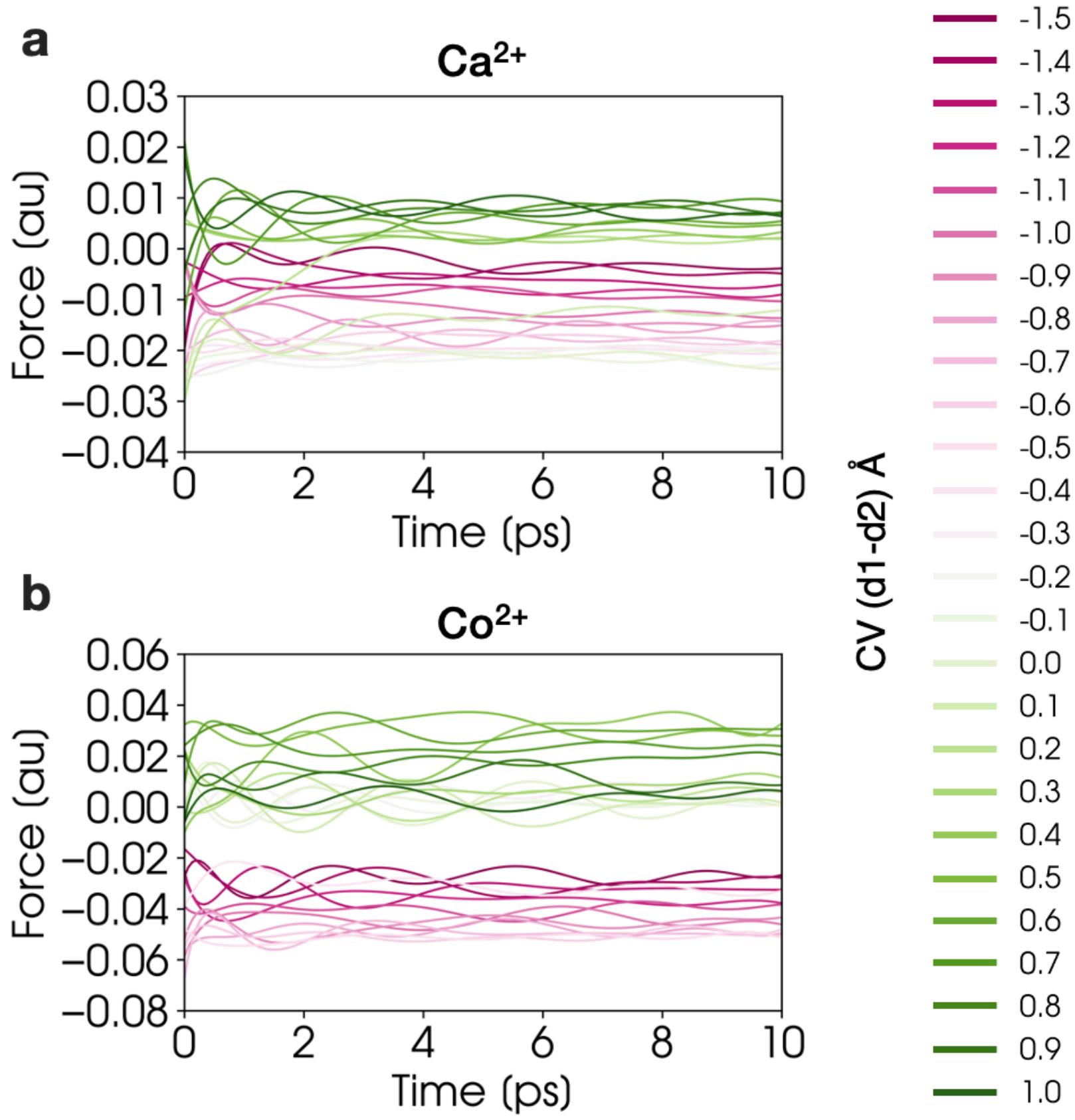

Supplementary Fig. 34. Convergence of QM/MM Thermodynamic Integration simulations in the presence of Ca^{2+} and Co^{2+}. The plots show the time evolution of constraint forces from Thermodynamic Integration simulations of DNA target strand cleavage in the presence of Ca^{2+} (**c**) and Co^{2+} (**d**). Data are presented for each Thermodynamic Integration window, color-coded according to the scale on the right, spanning ~10 ps of *ab-initio* QM/MM MD per window. The plots indicate force convergence after approximately 6 ps, with average forces from 6 to 10 ps utilized for integration within the Thermodynamic Integration formalism to obtain the free energy profiles shown in Fig. 5 and Supplementary Fig. 33.

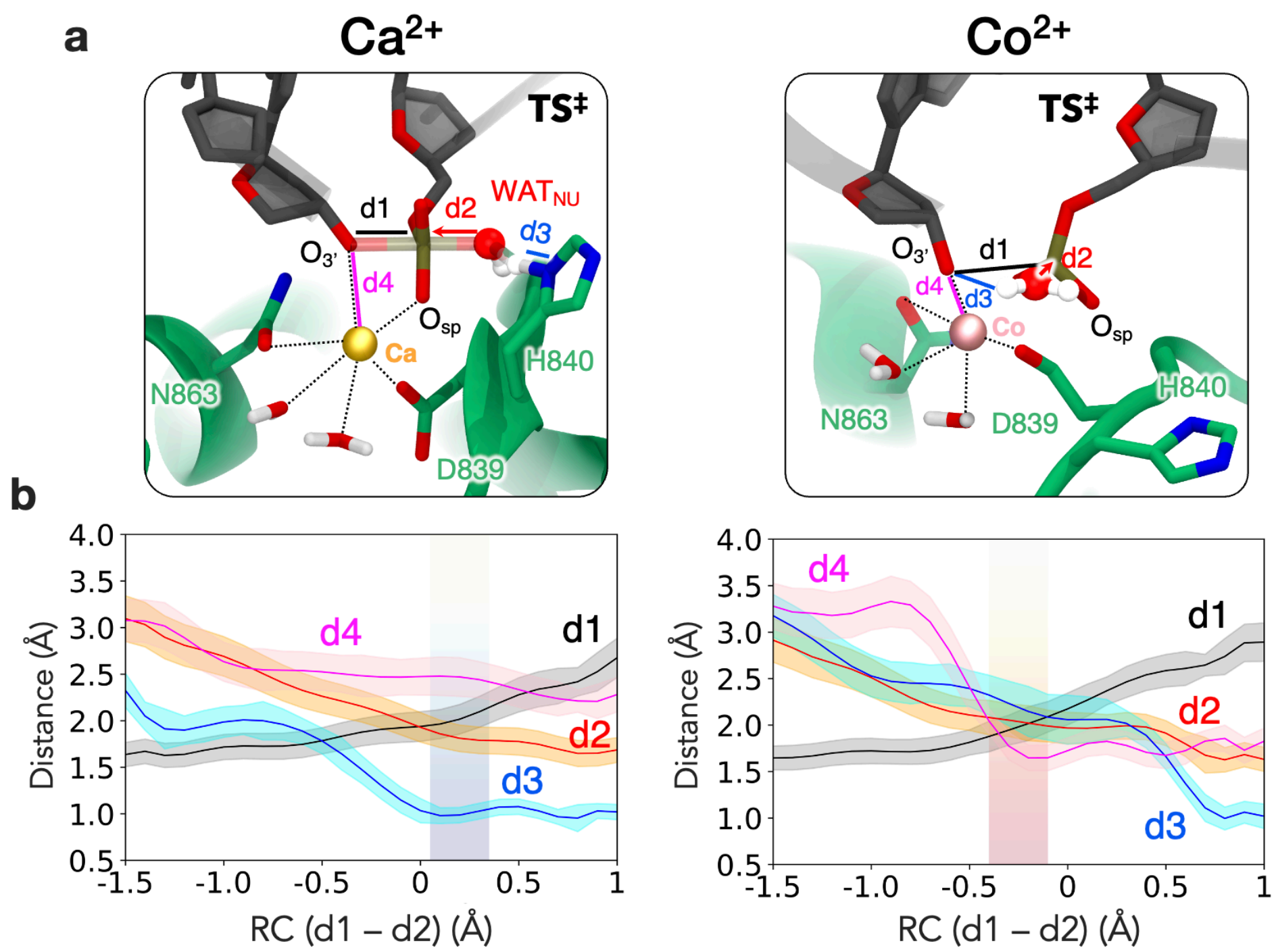

Supplementary Fig. 35. Details on the catalytic mechanism of target strand cleavage in the presence of Ca^{2+} and Co^{2+}. a. Transition state (TS‡) structures in the presence of Ca^{2+} (left) and Co^{2+} (right), highlighting the key distances involved in the chemical step. d1 is the distance between P_{SCI} and the O3' (breaking P–O bond distance), d2 is the distance between the water nucleophile (WAT_{nu}) and P_{SCI} (forming P–O bond distance). In the system bound to Ca^{2+}, d3 is the distance between the H840 base (N@H840) and the water nucleophile hydrogen (H@WAT_{nu}). In the system bound to Co^{2+}, d3 is the distance between the O3' and the water nucleophile hydrogen (H@WAT_{nu}). In this system, due to the displacement of H840 (Supplementary Figs. 21-22, and 23), H@WAT_{nu} is directly accepted by the O3'. In both systems, d4 is the distance between the metal ion and the O3'. **b.** Evolution of the key distances (d1 to d4) along phosphodiester bond cleavage, as computed through Thermodynamic Integration simulations in the presence of Ca^{2+} (left) and Co^{2+} (right). Data are computed along the reaction coordinate (RC, d1-d2) and averaged considering the last ~6 to 10 ps for each Thermodynamic Integration window (Supplementary Figs. 33-34), with shaded region representing the standard deviation. The TS‡ region is indicated using a vertical dashed bar. In the presence of Ca^{2+} (left), the catalysis follows an associative S_N2 mechanism, resulting in the formation and breaking of the P–O bonds at the TS‡. In the presence of Co^{2+} (right), due to the displacement of H840, a dissociative mechanism is observed, in which the breakage of the P–O3' bond happens first, followed by the proton transfer from the WAT_{nu} to O3'.

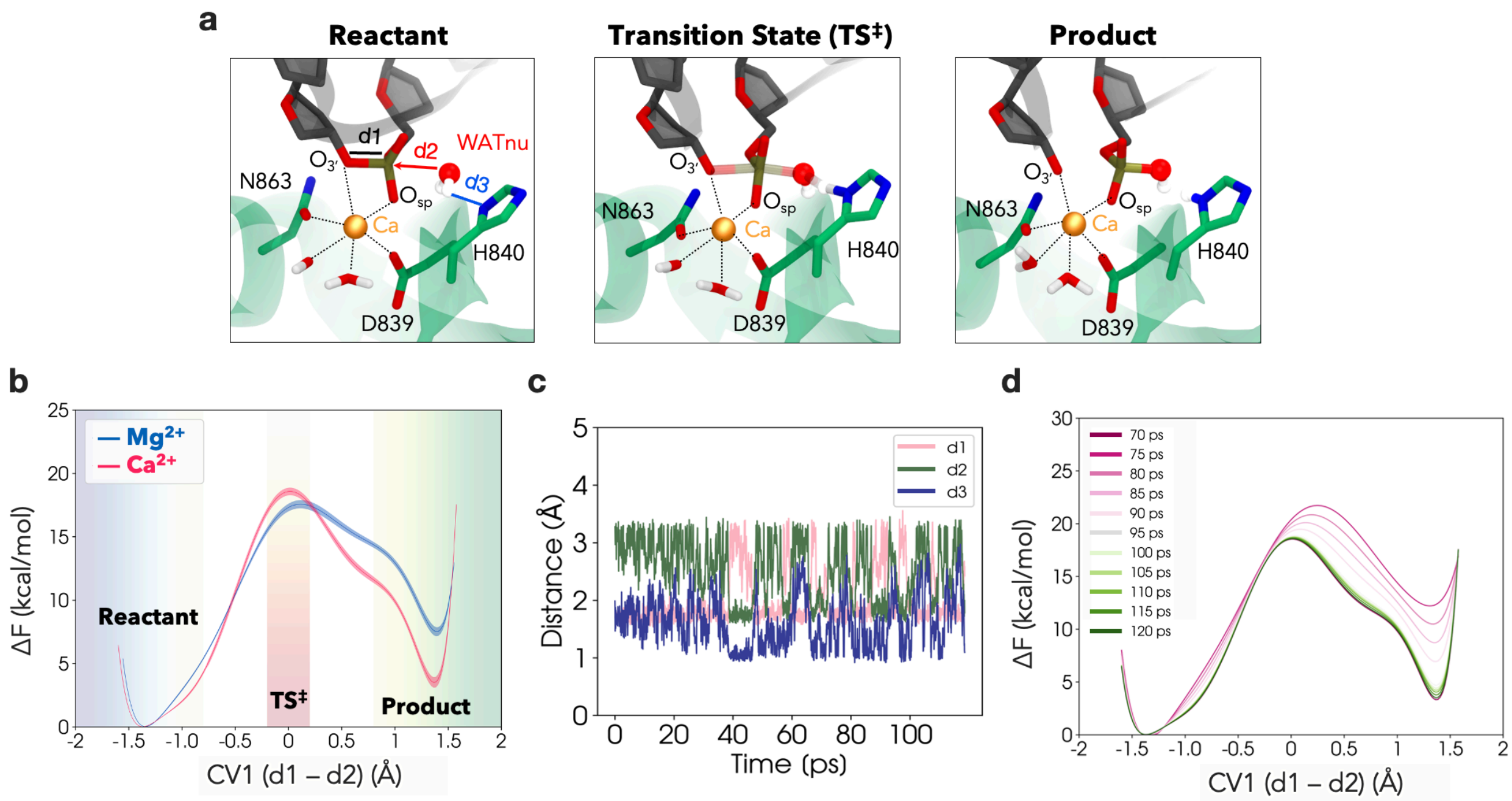

Supplementary Fig. 36. Catalytic mechanism of target strand cleavage in the presence of Ca^{2+} from QM/MM metadynamics simulations. a. Representative snapshots of the reactant, transition state (TS‡), and product states obtained from ~120 ps of QM/MM metadynamics simulations. Distances d1, d2, and d3 define the collective variables (CVs) used to describe phosphodiester bond cleavage and proton transfer. **b.** Minimum free energy pathway projected along the first CV1 for Ca^{2+} (red), compared with the corresponding profile obtained for Mg^{2+} (blue) using a similar QM/MM metadynamics approach reported by Nierzwicki et al[41]. **c.** Time evolution of the d1, d2, and d3 distances during ~120 ps of QM/MM metadynamics simulations. Multiple transitions between reactant and product states are observed, indicating extensive sampling of the transition region and convergence of the free energy profile. **d.** The convergence of the free energy profile is evaluated along CV1. The minimum free energy pathway, plotted at different time intervals (from ~70 ps to ~120 ps), exhibits a stable and converging energy barrier, confirming the reliability of metadynamics simulations.

a

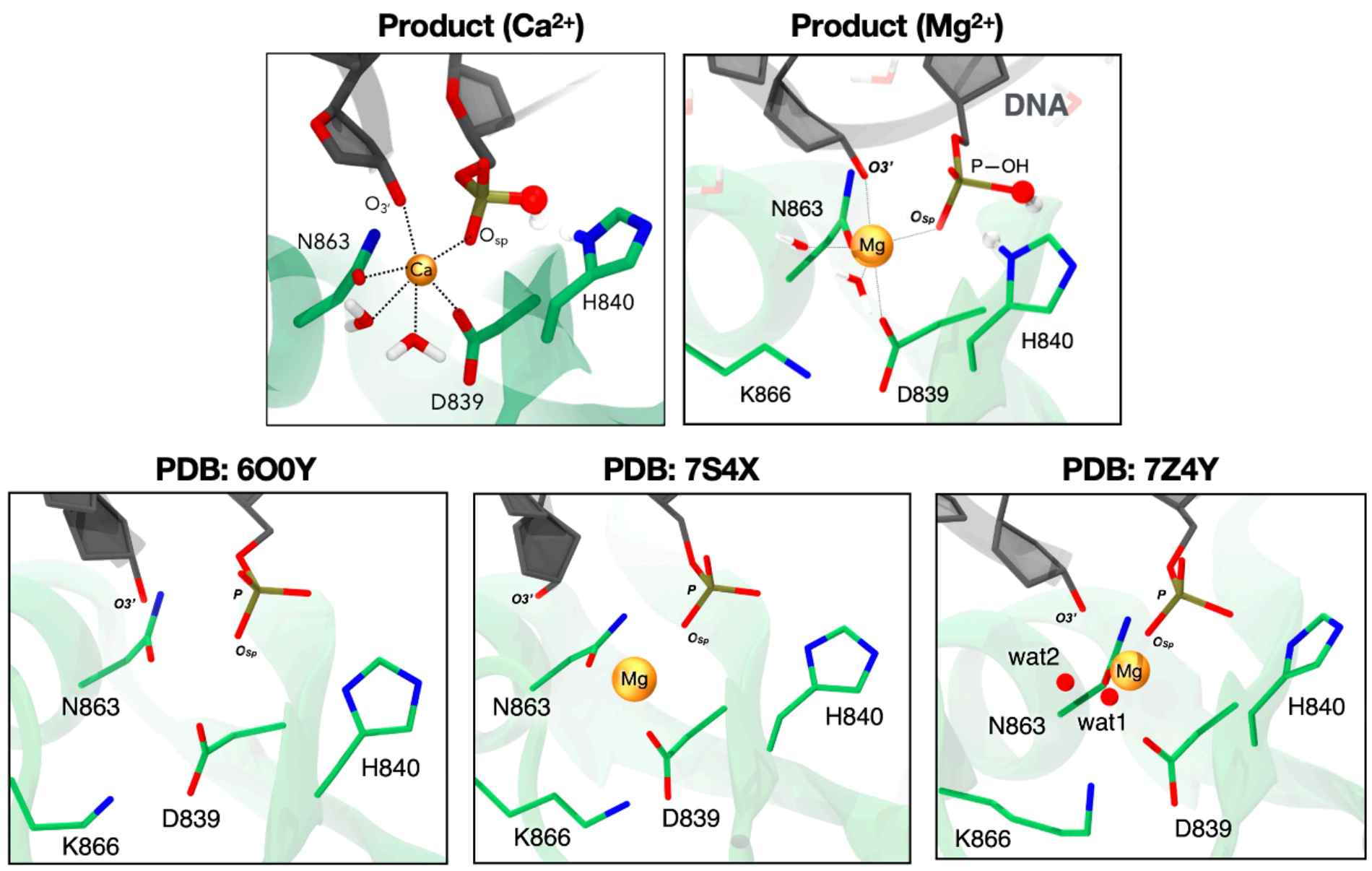

b

	Ca2+			Mg2+			Experimental Structures		
	**Reactant**	**TS‡**	**Product**	**Reactant**	**TS‡**	**Product**	**PDB: 7S4X**	**PDB: 7Z4Y**	**PDB: 6O0Y**
P_{SCI} - O3'	1.63 ± 0.13	1.93 ± 0.13	2.67 ± 0.20	1.65 ± 0.04	2.04 ± 0.06	2.33 ± 0.04	5.48	3.30	3.34
P_{SCI} - O_{WAT}	3.19 ± 0.14	1.93 ± 0.15	1.68 ± 0.13	3.13 ± 0.04	2.04 ± 0.06	1.74 ± 0.04	1.48	1.60	1.47
H_{WAT} - H840	2.32± 0.18	1.03 ± 0.13	1.02 ± 0.08	3.61 ± 0.36	1.02 ± 0.10	1.03 ± 0.03	-	-	-
M - O_{Sp}	2.31 ± 0.22	2.30 ± 0.19	2.28 ± 0.18	2.00 ± 0.08	2.02 ± 0.09	2.06 ± 0.08	2.47	1.86	-
M - O3'	3.07 ± 0.18	2.45 ± 0.13	2.20 ± 0.18	3.46 ± 0.20	2.85 ± 0.20	2.07 ± 0.08	4.34	2.31	-
M - D839	2.29 ± 0.16	2.37 ± 0.18	2.39 ± 0.19	1.99 ± 0.06	2.02 ± 0.07	2.14 ± 0.11	2.37	2.11	-
M - N863	2.45 ± 0.18	2.37 ± 0.19	2.39 ± 0.17	2.12 ± 0.08	2.03 ± 0.09	2.12 ± 0.08	3.05	2.05	-
M - wat1	2.39 ± 0.20	2.46 ± 0.19	2.44 ± 0.21	2.09 ± 0.11	2.11 ± 0.10	2.12 ± 0.12	-	2.11	-
M - wat2	2.47 ± 0.19	2.42 ± 0.18	2.48 ± 0.20	2.12 ± 0.10	2.13 ± 0.12	2.11 ± 0.10	-	1.93	-

Supplementary Fig. 37. Structural comparison of the HNH active site in the product state following target strand cleavage, based on QM/MM simulations and experimental structures. a. Structural representations of the product state following target strand (TS) cleavage in Cas9, obtained from QM/MM metadynamics simulations with Ca^{2+} (top, left) and Mg^{2+} (top, right). The structure with Mg^{2+} is adapted from Nierzwicki et al[41]. The bottom panels display experimental structures of Cas9 in the product state (PDB IDs: 6O0Y[2], 7S4X[66], and 7Z4Y[66]), illustrating the coordination environment in the absence of metal ions or in the presence of Mg^{2+}, as indicated. **b.** The table presents interatomic distances (in Å) for the reactant, transition state (TS‡), and product states, as obtained from QM/MM metadynamics simulations with Ca^{2+} and Mg^{2+}. Data for the simulation with Mg^{2+} are adapted from Nierzwicki et al [41]. These distances are compared with those observed in the cryo-EM structures mentioned above. Values are reported as mean ± standard deviation.

S4. Supplementary Tables

Supplementary Table 1: DSF-derived melting temperatures of Cas9 variants

**Condition**	**T_m (ºC)**	**T_m (ºC) SD**
**WT Cas9 apo**	**43.2**	**0.1**
**WT Cas9 + Mg^{2+}**	**43.3**	**0.2**
**WT Cas9 + Mg^{2+} + gRNA**	**47**	**0.1**
**M1 Cas9 apo**	**41.3**	**0.1**
**M1 Cas9 + Mg^{2+}**	**40.4**	**1**
**M1 Cas9 + Mg^{2+} + gRNA**	**46.7**	**0.1**
**M2 Cas9 apo**	**41**	**0**
**M2 Cas9 + Mg^{2+}**	**40.6**	**0.1**
**M2 Cas9 + Mg^{2+} + gRNA**	**44.3**	**0.1**
**M3 Cas9 apo**	**42.8**	**0.1**
**M3 Cas9 + Mg^{2+}**	**43**	**0.3**
**M3 Cas9 + Mg^{2+} + gRNA**	**46.4**	**0.2**

Supplementary Table 2: Primers used in site-directed mutagenesis of spCas9 for generation of cluster mutants

**Primer Name**	**Sequence**
M1_Forward	5' ggtgctACAGCGGAAGCGACTCGT
M1_Reverse	5'tgcggcAAATAAAAGAGCCCCTATAAGATTTTTTTTGATAC
M2_Foward	5' gtcgccACTCGCCAAATCACTAAG
M2_Reverse	5' aagagcGCGTTTGATAAAACCAGC
M3_Forward	5' gcgtcaGTCGGATGGGCGGTGATC
M3_Reverse	5' cgcaccGATATCTAAGCCTATTGAGTATTTCTTATCC

Supplementary Table 3: Sequences used in cleavage experiments

**Name**	**Sequence (5'-3')**	**Source**
TS Cleavage	TS: /6-FAM/agc tga cgt ttg tac tcc agc gtc tca tct tta tgc gtc agc aga gat ttc tgc t<br>NTS: agc aga aat ctc tgc tga cgc ata aag atg aga cgc tgg agt aca aac gtc agc t	IDT

NTS Cleavage	TS: agc tga cgt ttg tac tcc agc gtc tca tct tta tgc gtc agc aga gat ttc tgc t<br>NTS: /6-FAM/agc aga aat ctc tgc tga cgc ata aag atg aga cgc tgg agt aca aac gtc agc t	IDT
sgRNA	GACGCAUAAAGAUGAGACGC + 80-mer SpCas9 scaffold	Synthego

S5. Raw Data

Raw Gel images Figure S14

WTCasp9 (MG)

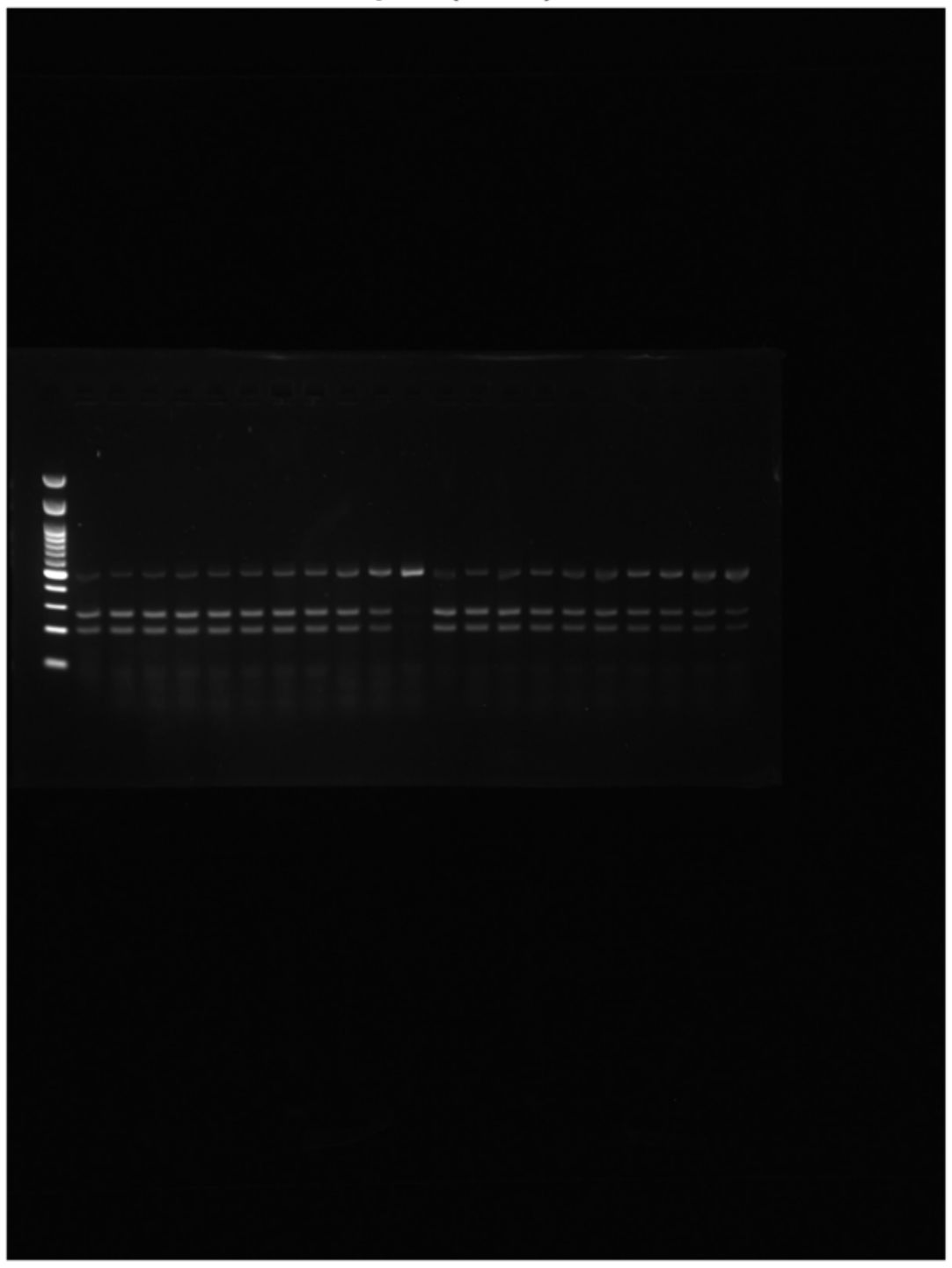

WTCasp9 (Li)

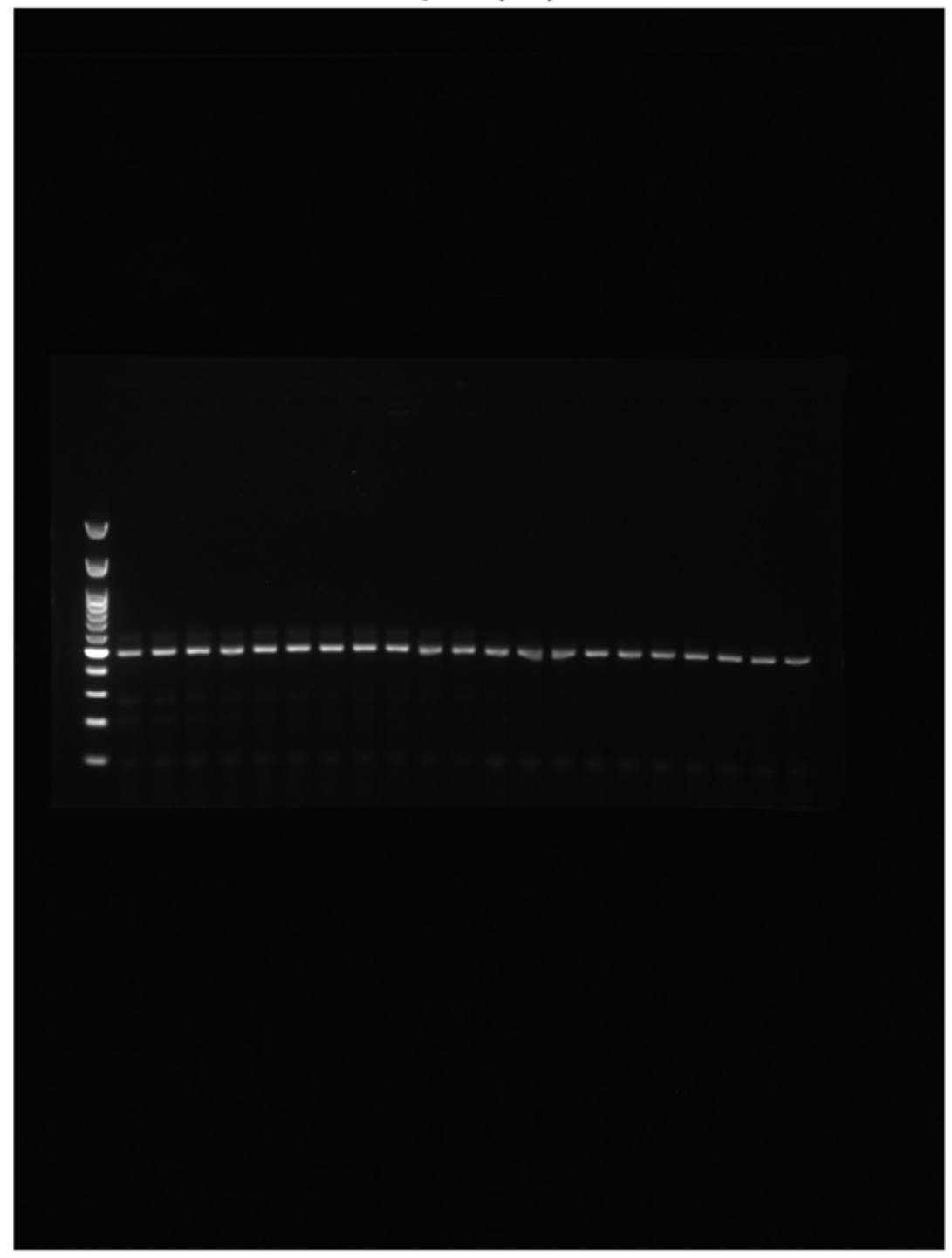

WTCasp9 (AL)

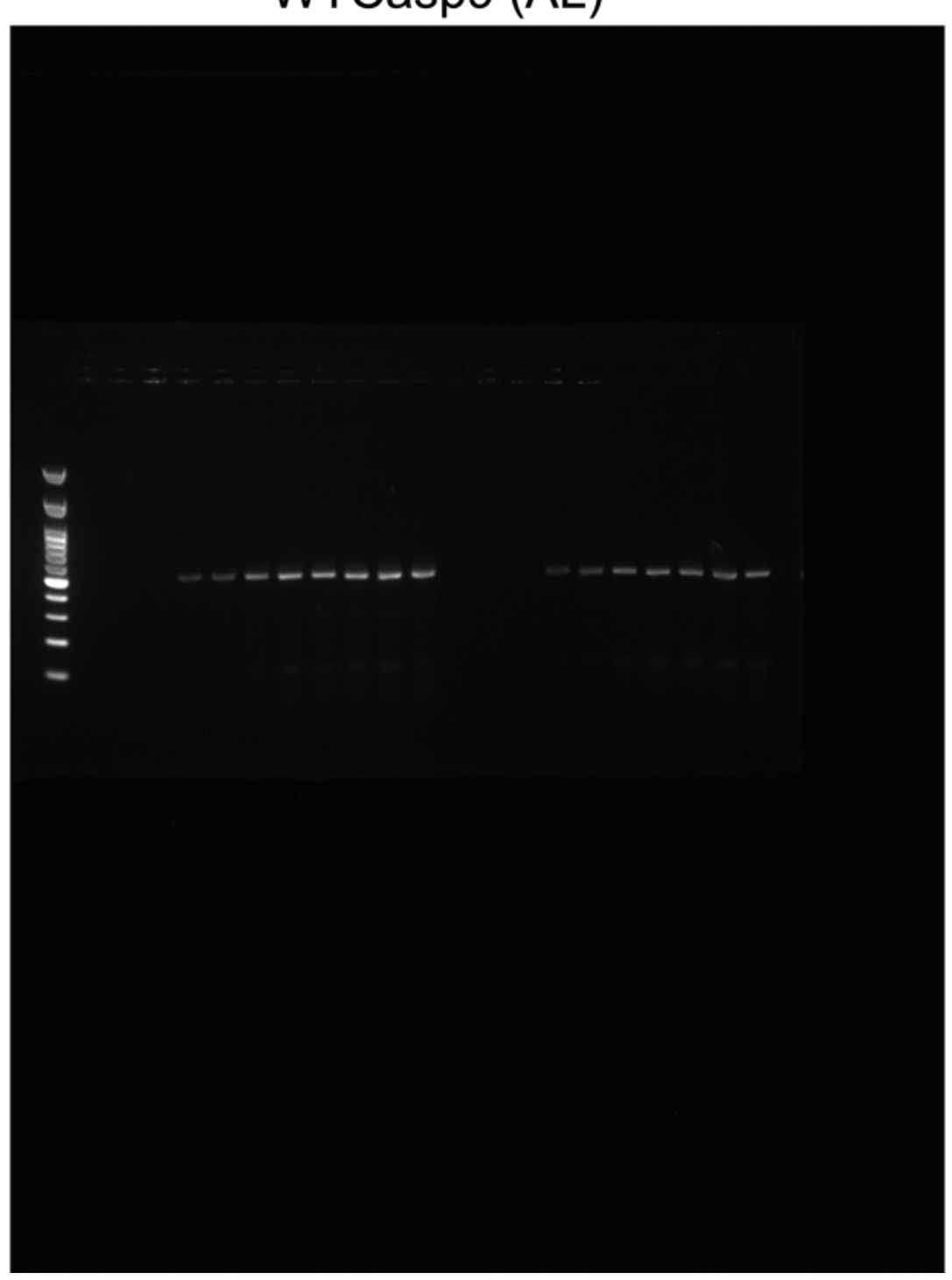

Raw Gel Images Figure S23

WTCasp9 rep1

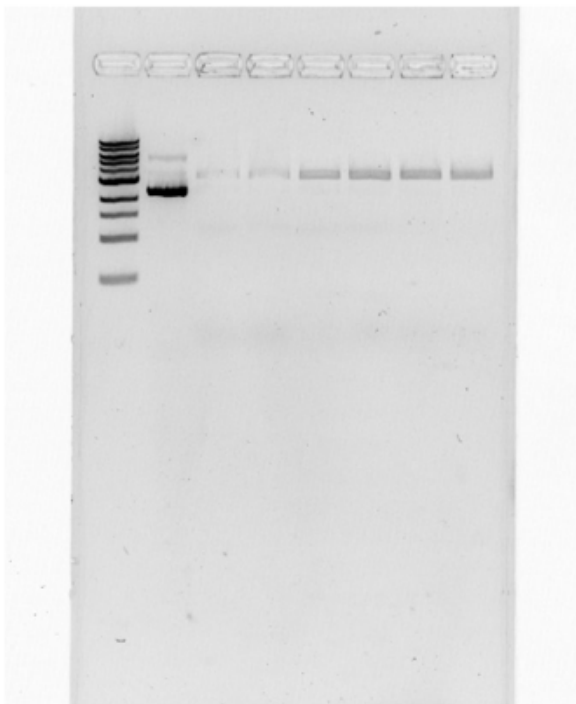

M1 rep1

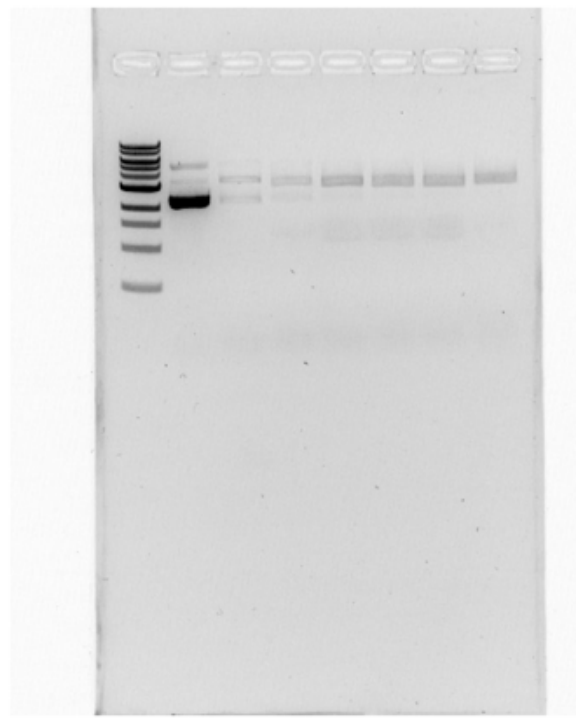

M2 rep1

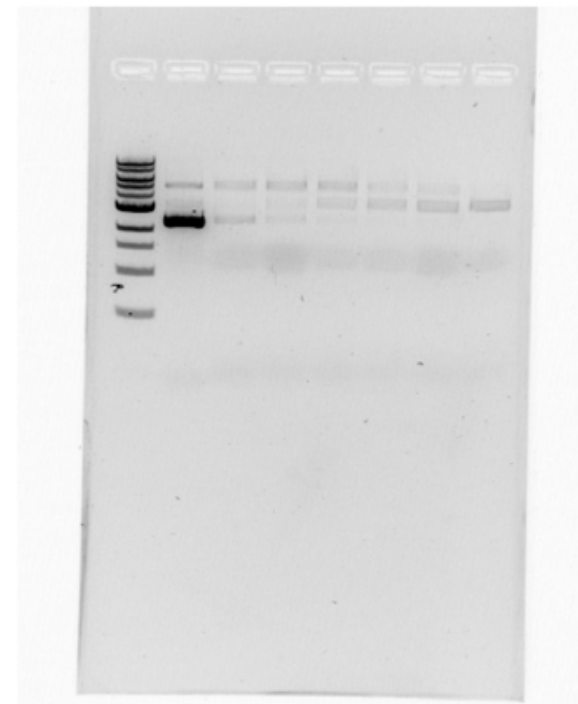

M3 rep1

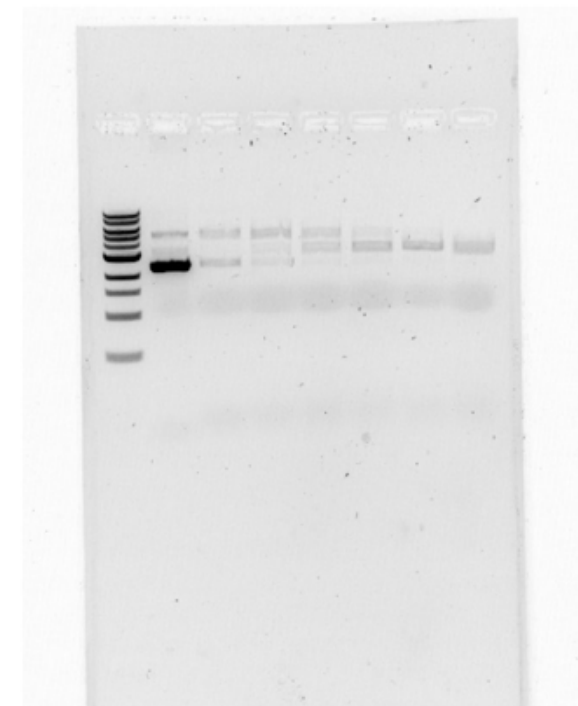

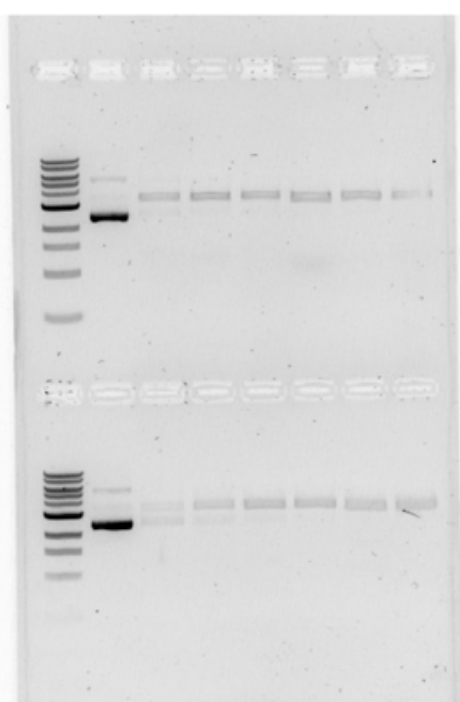

WTCasp9 rep2

M1 rep2

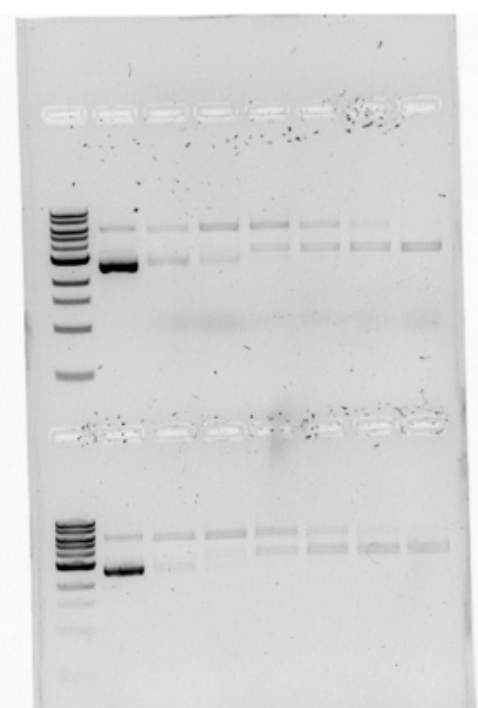

M2 rep2

M3 rep2

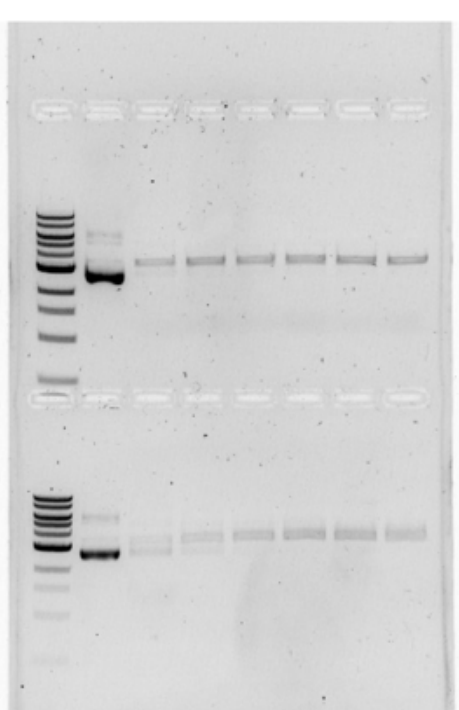

WTCasp9 rep3

M1 rep3

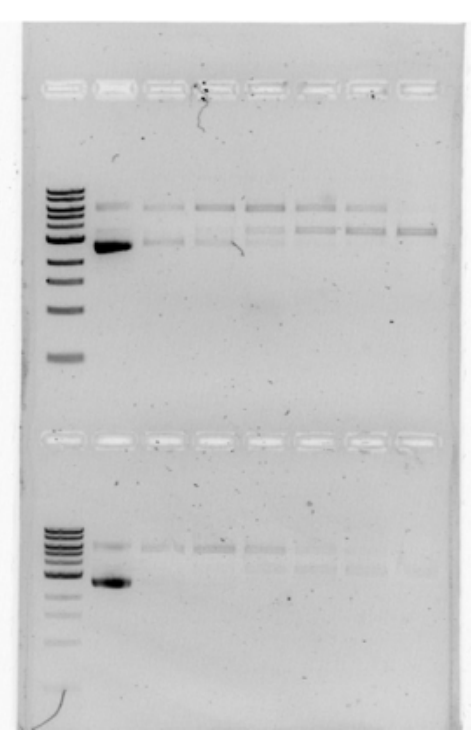

M2 rep3

M3 rep3

S6. Supplementary References

1. Jiang, F. *et al.* Structures of a CRISPR-Cas9 R-loop complex primed for DNA cleavage. *Science* **351**, 867–871 (2016).
2. Zhu, X. *et al.* Cryo-EM structures reveal coordinated domain motions that govern DNA cleavage by Cas9. *Nat. Struct. Mol. Biol.* **26**, 679–685 (2019).
3. Sinha, S., Pindi, C., Ahsan, M., Arantes, P. R. & Palermo, G. Machines on Genes through the Computational Microscope. *J. Chem. Theory Comput.* **19**, 1945–1964 (2023).
4. Saha, A. *et al.* An alpha-helical lid guides the target DNA toward catalysis in CRISPR-Cas12a. *Nat. Commun.* **15**, 1473 (2024).
5. Arantes, P. R. *et al.* Dimerization of the Deaminase Domain and Locking Interactions with Cas9 Boost Base Editing Efficiency in ABE8e. *Nucleic Acids Res.* **52**, 13931–13944 (2024).
6. Sinha, S. *et al.* Unveiling the RNA-mediated allosteric activation discloses functional hotspots in CRISPR-Cas13a. *Nucleic Acids Res.* **52**, 906–920 (2024).
7. Schwartz, E. A. *et al.* RNA targeting and cleavage by the type III-Dv CRISPR effector complex. *Nat. Commun.* **15**, 3324 (2024).
8. Tian, C. *et al.* ff19SB: Amino-Acid-Specific Protein Backbone Parameters Trained against Quantum Mechanics Energy Surfaces in Solution. *J. Chem. Theory Comput.* **16**, 528–552 (2020).
9. Galindo-Murillo, R. *et al.* Assessing the Current State of Amber Force Field Modifications for DNA. *J. Chem. Theory Comput.* **12**, 4114–4127 (2016).
10. Zgarbova, M. *et al.* Refinement of the Cornell et al. Nucleic Acids Force Field Based on Reference Quantum Chemical Calculations of Glycosidic Torsion Profiles. *J. Chem. Theory Comput.* **7**, 2886–2902 (2011).
11. Jorgensen, W. L., Chandrasekhar, J., Madura, J. D., Impey, R. W. & Klein, M. L. Comparison of simple potential functions for simulating liquid water. *J. Chem. Phys.* **79**, 926–935 (1983).
12. Li, P., Roberts, B. P., Chakravorty, D. K. & Merz, K. M. Rational Design of Particle Mesh Ewald Compatible Lennard-Jones Parameters for +2 Metal Cations in Explicit Solvent. *J. Chem. Theory Comput.* **9**, 2733–2748 (2013).
13. Li, P. & Merz, K. M. Taking into Account the Ion-Induced Dipole Interaction in the Nonbonded Model of Ions. *J. Chem. Theory Comput.* **10**, 289–297 (2014).
14. Li, Z., Song, L. F., Li, P. & Merz, K. M. Systematic Parametrization of Divalent Metal Ions for the OPC3, OPC, TIP3P-FB, and TIP4P-FB Water Models. *J. Chem. Theory Comput.* **16**, 4429–4442 (2020).
15. Allnér, O., Nilsson, L. & Villa, A. Magnesium Ion–Water Coordination and Exchange in Biomolecular Simulations. *J. Chem. Theory Comput.* **8**, 1493–1502 (2012).
16. Turq, P., Lantelme, F. & Friedman, H. L. Brownian Dynamics: Its Applications to Ionic Solutions. *J. Chem. Phys.* **66**, 3039 (1977).
17. Berendsen, H. J. C., Postma, J. P. M., van Gunsteren, W. F., DiNola, A. & Haak, J. R. Molecular Dynamics with Coupling to an External Bath. *J. Chem. Phys.* **81**, 3684 (1984).

18. Dagdas, Y. S., Chen, J. S., Sternberg, S. H. & Doudna, J. A. A Conformational Checkpoint between DNA Binding and Cleavage by CRISPR-Cas9. *Sci. Adv.* **3**, eaao002 (2017).

19. Raper, A. T., Stephenson, A. A. & Suo, Z. Functional Insights Revealed by the Kinetic Mechanism of CRISPR/Cas9. *J Am Chem Soc* **140**, 2971−2984 (2018).

20. Case, D. A. *et al.* AMBER 2022. *University of California, San Francisco* (2020).

21. Li, P., Song, L. F. & Merz, K. M. Systematic Parameterization of Monovalent Ions Employing the Nonbonded Model. *J. Chem. Theory Comput.* **11**, 1645–1657 (2015).

22. Li, P., Song, L. F. & Merz, K. M. Parameterization of Highly Charged Metal Ions Using the 12-6-4 LJ-Type Nonbonded Model in Explicit Water. *J. Phys. Chem. B* **119**, 883–895 (2015).

23. Kästner, J. Umbrella sampling. *Wiley Interdiscip Rev Comput Mol Sci* **1**, 932–942 (2011).

24. Kumar, S., Rosenberg, J. M., Bouzida, D., Swendsen, R. H. & Kollman, P. A. THE weighted histogram analysis method for free-energy calculations on biomolecules. I. The method. *J. Comput. Chem.* **13**, 1011–1021 (1992).

25. Hub, J. S., de Groot, B. L. & van der Spoel, D. g_wham—A Free Weighted Histogram Analysis Implementation Including Robust Error and Autocorrelation Estimates. *J. Chem. Theory Comput.* **6**, 3713–3720 (2010).

26. Chodera, J. D. & Noé, F. Markov state models of biomolecular conformational dynamics. *Curr. Opin. Struct. Biol.* **25**, 135–144 (2014).

27. Pande, V. S., Beauchamp, K. & Bowman, G. R. Everything you wanted to know about Markov State Models but were afraid to ask. *Methods* **52**, 99–105 (2010).

28. Scherer, M. K. *et al.* PyEMMA 2: A Software Package for Estimation, Validation, and Analysis of Markov Models. *J. Chem. Theory Comput.* **11**, 5525–5542 (2015).

29. Sternberg, S. H., LaFrance, B., Kaplan, M. & Doudna, J. A. Conformational control of DNA target cleavage by CRISPR–Cas9. *Nature* **527**, 110–113 (2015).

30. Noé, F. & Clementi, C. Kinetic Distance and Kinetic Maps from Molecular Dynamics Simulation. *J. Chem. Theory Comput.* **11**, 5002–5011 (2015).

31. Sculley, D. Web-scale k-means clustering. *Proceedings of the 19th international conference on World wide web (ACM, New York, NY, USA),* 1177–1178 (2010). doi:10.1145/1772690.1772862.

32. Arthur, D. & Vassilvitskii, S. K-means++: the advantages of careful seeding. in *Proceedings of the Eighteenth Annual ACM-SIAM Symposium on Discrete Algorithms (Society for Industrial and Applied Mathematics, USA),* 1027–1035 (2007).

33. Scherer, M. K. *et al.* Variational selection of features for molecular kinetics. *J Chem Phys* **150**, 194108 (2019).

34. Swope, W. C., Pitera, J. W. & Suits, F. Describing Protein Folding Kinetics by Molecular Dynamics Simulations. 1. Theory. *J. Phys. Chem. B* **108**, 6571–6581 (2004).

35. Noé, F. Statistical inefficiency of Markov model count matrices. *Preprint at Freie Universitat Berlin.* (2015) *https://publications.imp.fu-berlin.de/1699/1/autocorrelation_counts.pdf*.

36. Trendelkamp-Schroer, B., Wu, H., Paul, F. & Noé, F. Estimation and uncertainty of reversible Markov models. *J Chem Phys* **143**, (2015).

37. Prinz, J.-H. *et al.* Markov models of molecular kinetics: Generation and validation. *J. Chem. Phys.* **134**, (2011).

38. Röblitz, S. & Weber, M. Fuzzy spectral clustering by PCCA+: application to Markov state models and data classification. *Adv. Data Anal. Classif.* **7**, 147–179 (2013).

39. Noé, F., Schütte, C., Vanden-Eijnden, E., Reich, L. & Weikl, T. R. Constructing the equilibrium ensemble of folding pathways from short off-equilibrium simulations. *Proc. Natl. Acad. Sci. U. S. A.* **106**, 19011–19016 (2009).

40. Metzner, P., Schütte, C. & Vanden-Eijnden, E. Transition Path Theory for Markov Jump Processes. *Multiscale Model. Sim.* **7**, 1192–1219 (2009).

41. Nierzwicki, Ł. *et al.* Principles of target DNA cleavage and the role of Mg2+ in the catalysis of CRISPR–Cas9. *Nat. Catal.* **5**, 912–922 (2022).

42. Becke, A. D. Density-functional exchange-energy approximation with correct asymptotic behavior. *Phys. Rev. A* **38**, 3098–3100 (1988).

43. Lee, C., Yang, W. & Parr, R. G. Development of the Colle-Salvetti correlation-energy formula into a functional of the electron density. *Phys. Rev. B* **37**, 785–789 (1988).

44. Parrinello, M., Andreoni, W. & Curioni, A. CPMD; IBM. Corporation and Max-Planck Institut: Armonk, New York and. Stuttgart, Germany (2000).

45. Troullier, N. & Martins, J. L. Efficient pseudopotentials for plane-wave calculations. II. Operators for fast iterative diagonalization. *Phys. Rev. B* **43**, 8861–8869 (1991).

46. Martyna, G. J. & Tuckerman, M. E. A reciprocal space based method for treating long range interactions in *ab initio* and force-field-based calculations in clusters. *J. Chem. Phys.* **110**, 2810–2821 (1999).

47. Laio, A., VandeVondele, J. & Rothlisberger, U. A Hamiltonian electrostatic coupling scheme for hybrid Car–Parrinello molecular dynamics simulations. *J. Chem. Phys.* **116**, 6941–6947 (2002).

48. Ciccotti, G., Ferrario, M., Hynes, J. T. & Kapral, R. Constrained molecular dynamics and the mean potential for an ion pair in a polar solvent. *Chem. Phys.* **129**, 241–251 (1989).

49. Carter, E. A., Ciccotti, G., Hynes, J. T. & Kapral, R. Constrained reaction coordinate dynamics for the simulation of rare events. *Chem. Phys. Lett.* **156**, 472–477 (1989).

50. Ivanov, I., Tainer, J. A. & McCammon, J. A. Unraveling the three-metal-ion catalytic mechanism of the DNA repair enzyme endonuclease IV. *Proc. Natl. Acad. Sci. U. S. A.* **104**, 1465–70 (2007).

51. Casalino, L., Palermo, G., Rothlisberger, U. & Magistrato, A. Who Activates the Nucleophile in Ribozyme Catalysis? An Answer from the Splicing Mechanism of Group II Introns. *J. Am. Chem. Soc.* **138**, 10374–10377 (2016).

52. Laio, A. & Parrinello, M. Escaping free-energy minima. *Proc. Natl. Acad. Sci. U. S. A.* **99**, 12562–6 (2002).

53. Iannuzzi, M., Laio, A. & Parrinello, M. Efficient Exploration of Reactive Potential Energy Surfaces Using Car-Parrinello Molecular Dynamics. *Phys. Rev. Lett.* **90**, 4 (2003).

54. Gong, S., Yu, H. H., Johnson, K. A. & Taylor, D. W. DNA Unwinding Is the Primary Determinant of CRISPR-Cas9 Activity. *Cell Rep.* **22**, 359–371 (2018).

55. Delaglio, F. *et al.* NMRPipe: A Multidimensional Spectral Processing System Based on UNIX Pipes. *J. Biomol. NMR* **6**, 277–293 (1995).

56. Lee, W., Tonelli, M. & Markley, J. L. NMRFAM-SPARKY: Enhanced software for biomolecular NMR spectroscopy. *Bioinformatics* **31**, 1325–1327 (2015).

57. Keller, R. L. J. Optimizing the process of nuclear magnetic resonance spectrum analysis and computer aided resonance assignment. *Diss., Naturwissenschaften, Eidgenössische Technische Hochschule ETH Zürich*, Nr. 15947 (2005).

58. Grzesiek, S. & Bax, A. An efficient experiment for sequential backbone assignment of medium-sized isotopically enriched proteins. *J. Magn. Res.* **99**, 201–207 (1992).

59. Belato, H. B., East, K. W. & Lisi, G. P. 1H, 13C, 15N backbone and side chain resonance assignment of the HNH nuclease from Streptococcus pyogenes CRISPR-Cas9. *Biomol. NMR Assign.* **13**, 367–370 (2019).

60. Grzesiek, S., Stahl, S. J., Wingfield, P. T. & Bax, A. The CD4 Determinant for Downregulation by HIV-1 Nef Directly Binds to Nef. Mapping of the Nef Binding Surface by NMR. *Biochemistry* **35**, 10256–10261 (1996).

61. Kay, L. E., Torchia, D. A. & Bax, A. Backbone dynamics of proteins as studied by nitrogen-15 inverse detected heteronuclear NMR spectroscopy: application to staphylococcal nuclease. *Biochemistry* **28**, 8972–8979 (1989).

62. Loria, J. P., Rance, M. & Palmer 3rd, A. G. A Relaxation-Compensated Carr-Purcell-Meiboom-Gill Sequence for Characterizing Chemical Exchange by NMR Spectroscopy. *J. Am. Chem. Soc.* **121**, 2331–2332 (1999).

63. Markley, J. L. Observation of histidine residues in proteins by nuclear magnetic resonance spectroscopy. *Acc. Chem. Res.* **8**, 70–80 (1975).

64. Wu, T. *et al.* Three Essential Resources to Improve Differential Scanning Fluorimetry (DSF) Experiments. Preprint at: https://doi.org/10.1101/2020.03.22.002543 (2020).

65. Li, P. & Merz, K. M. Metal Ion Modeling Using Classical Mechanics. *Chem. Rev.* **117**, 1564–1686 (2017).

66. Bravo, J. P. K. *et al.* Structural basis for mismatch surveillance by CRISPR–Cas9. *Nature* **603**, 343–347 (2022).
