# Supplementary material for: A cryptic binding pocket regulates the metal-dependent activity of Cas9
Source: Nat Commun. 2026 Oct 9;17:11501. doi: 10.1038/s41467-026-78458-0 (PMC13745514; doi:10.1038/s41467-026-78458-0)
Supplement: Supplementary file 3 [file 41467_2026_78458_MOESM3_ESM.pdf]

nature portfolio

Peer Review File

A Cryptic Binding Pocket Regulates the Metal-Dependent Activity of Cas9

Corresponding Author: Professor Giulia Palermo

This file contains all reviewer reports in order by version, followed by all author rebuttals in order by version.

Version 1:

Reviewer comments:

Reviewer #1

(Remarks to the Author)
The authors have made a serious and thorough effort to address this reviewer's concerns, and the rebuttal is direct and well supported by new simulations and experiments. A lingering doubt remains regarding the functional significance of the cryptic divalent metal binding pocket (DBP) identified by simulation. The pocket is not resolved in any published cryo-EM structure of SpCas9 and, by the authors' own Markov state model, is only transiently populated (~100 ns lifetime); its occupancy and the associated activation transitions are further reduced at near-physiological (2 mM) Mg2+. Together these observations leave open how much the DBP contributes to Cas9 activity under cellular conditions, as opposed to the 10 mM in vitro regime in which the mechanism is most clearly observed. That said, the mutational data, reinforced in revision by stability, nucleic-acid-binding, and ITC controls, argue convincingly that perturbing these residues impairs cleavage through a metal-dependent rather than a globally destabilizing mechanism. With all things considered, I recommend the acceptance of this manuscript based on its solid technical foundation, extensive data, and its contribution to an important model system for the understanding of RNA-guided processes.

Reviewer #2

(Remarks to the Author)
The authors have addressed my previous comments satisfactorily. I am now supportive of its publication in Nature Communications.

Reviewer #4

(Remarks to the Author)
The authors have addressed my concerns with multiple independent experiments (NMR, ITC etc.) and calculations - I believe this only makes the manuscript stronger ruling out parallel or competing interpretations. I recommend publication as is!

Open Access This Peer Review File is licensed under a Creative Commons Attribution 4.0 International License, which permits use, sharing, adaptation, distribution and reproduction in any medium or format, as long as you give appropriate credit to the original author(s) and the source, provide a link to the Creative Commons license, and indicate if changes were made.
In cases where reviewers are anonymous, credit should be given to 'Anonymous Referee' and the source.
The images or other third party material in this Peer Review File are included in the article's Creative Commons license, unless indicated otherwise in a credit line to the material. If material is not included in the article's Creative Commons license and your intended use is not permitted by statutory regulation or exceeds the permitted use, you will need to obtain permission directly from the copyright holder.
To view a copy of this license, visit https://creativecommons.org/licenses/by/4.0/

We thank the reviewers for their thoughtful evaluation of our manuscript and for their constructive comments, which helped us improve the clarity and strength of the study. In response to the reviewers' suggestions, we have performed additional molecular dynamics (MD) simulations and carried out new NMR and biochemical experiments, further supporting the conclusions of this work. We have now included 12 new Supplementary Figures (**Supplementary Figs. 13, 14, 18–22, 25–29**) and revised the manuscript throughout to incorporate these additional data and analyses. In addition, a new section of Supplementary Results has been included (**S2**), and four new sections have been added to the Supplementary Methods (**S1.3, S1.4, S1.7, S1.13**) describing the expanded simulation protocols and analyses, thermal stability measurements, nucleic acid binding experiments, and additional biochemical characterization. To provide a more balanced integration of the computational and experimental components of the study, we have now introduced a new **Fig. 4** dedicated to the experimental results supporting our main conclusions.

Reviewer #1 (Remarks to the Author):

The manuscript by Ahsan used molecular dynamics simulation to reveal the presence of a cryptic divalent metal ion binding site in SpCas9 when its HNH nuclease domain moves from pre-active conformation (PDB 5F9R) to the active conformation (6O0Y). High Mg2+ concentration eased the conformational transition whereas low Mg2+ concentration penalized the transition. In support of the simulation results, the authors showed that mutating the metal-coordinating residues reduced DNA cleavage by SpCas9, especially on the TS strand. The authors further deduced information from the simulation about the impact of different metal ions on the geometry of the HNH active site.

The in-depth dissection of cryptic metal binding site is potentially appealing to the general public, given that similar phenomenon may also exist in Cas12 (Nguyen et al., NAR, 2024). This reviewer focuses on the functional interpretation of this metal binding site, and on whether such mechanisms are evolutionarily conserved, etc. I have the following major points for the authors to address.

1. How rate limiting is the conformational transition from pre-cleavage to the active conformation? SpCas9 and its DNA substrate undergo large conformational changes before adopting the pre-cleavage state in 5F9R. These changes include dsDNA melting, R-loop formation, and conformational changes in the RECIII and HNH domains. The changes are depicted in PDBs 7Z4C, 7Z4D, 7Z4E, 7Z4I, and the parallel set of structures by coauthor David Taylor's laboratory. The cryptic metal binding site cannot form in the early state (up to 13-bp R-loop at least). If SpCas9 dwell much longer in these early states a than in the transition from 5F9R to 6o0Y, the latter would not significantly impact SpCas9 cleavage.

We thank the reviewer for this thoughtful comment and for highlighting the importance of the early stages of SpCas9 activation, including R-loop formation and large-scale conformational rearrangements. We agree that these early steps are relatively slow and constitute the primary kinetic bottleneck during target recognition (Gong et al., Cell Rep 2018).

The late-stage conformational activation of the HNH domain remains an obligatory and mechanistically distinct step required for catalysis and is coupled to divalent metal ions. Single-molecule and kinetic studies (e.g., Dagdas et al., Sci. Adv. 2017; Raper et al., JACS 2018; Wang et al., PNAS 2020) have established a multi-step activation pathway in which early and intermediate HNH conformations – including undocked and partially rearranged states – are accessible in the absence of divalent ions, whereas efficient population of the catalytically competent docked HNH conformation requires divalent cations.

Single-molecule studies (e.g., Wang et al., PNAS 2020) further support that, even in late-stage ternary complexes such as the pre-active state represented by PDB 5F9R, the HNH domain undergoes an additional conformational transition to reach the reaction-competent state. In line with previous work (Dagdas et al., Sci. Adv. 2017; Raper et al., JACS 2018), this late activation step is coupled to divalent ion binding and associated with final catalytic positioning at the cleavage site. Thus, while earlier steps establish target recognition and partial activation, cleavage requires completion of this final conformational transition.

We also agree with the reviewer that the cryptic metal-binding pocket identified in our work is unlikely to be present in early R-loop intermediates (e.g., structures with partial R-loop formation such as PDBs 7Z4C-I). Consistent with this observation, our results support a model in which this pocket is an emergent feature of late-stage activation, becoming accessible only after sufficient R-loop maturation and reorganization of the HNH–RuvC–DNA interface. Thus, rather than influencing early recognition steps, the cryptic site modulates the efficiency and metal dependence of the final activation step.

In the revised manuscript, we have clarified this point to emphasize that while early conformational transitions govern target engagement, the late HNH-docking transition represents an obligatory, divalent ion–coupled step required for catalysis. This clarification has been incorporated in the Introduction (***pages 3-4, lines 69-74***).

2. Is there a reason to use 10 mM Mg2+ instead of 2 mM to simulate the high-Mg state? This reviewer is not sure if the free Mg2+ concentration inside the cell could ever reach 10 mM. 2 mM sounds more physiological. Have the authors explored the range of Mg2+ concentrations to see if 2 mM is sufficient to trigger the conformational change?

The reviewer correctly points out that free Mg^{2+} levels in cells are typically in the low millimolar range. Our choice of 10 mM Mg^{2+} was motivated by the need to directly match the conditions used in biophysical and single-molecule studies. Specifically, studies characterizing HNH conformational dynamics and activation (e.g., Dagdas et al., Sci. Adv. 2017; Raper et al., JACS 2018) commonly employ Mg^{2+} concentrations in the 5-10 mM range. Furthermore, our own biochemical cleavage assays (Fig. 4) were performed at 10 mM Mg^{2+}.

To address the reviewer's question regarding lower concentrations, we performed additional simulations at 2 mM Mg^{2+}. These simulations show that the same conformational mechanism is preserved; however, the occupancy of the cryptic ion-binding pocket and the frequency of transitions toward the active state are reduced compared to 10 mM. This behavior supports a model in which Mg^{2+} shifts the conformational equilibrium toward the active state, consistent with the use of 5-10 mM Mg^{2+} concentrations in in vitro studies of Cas9 activation.

We have revised the Results (***page 10, lines 197-198***) and the Methods sections (***page 23, lines 500-501***) to report that our simulation conditions were chosen to mirror the *in vitro* experimental regime (10 mM Mg^{2+}) rather than the cytosolic environment. We have also added a brief discussion on the results of our 2 mM simulations to demonstrate that the identified cryptic pocket remains functionally relevant at lower millimolar concentrations (***page 11, lines 226-229***). Data are reported in a new ***Supplementary Fig. 18.***

3. Regarding the impact of Ca2+ and Co2+ on the HNH active site: the authors argue that Ca2+ does not distort active site geometry. However, Ca2+ typically slows down DNA cleavage due to the subtle perturbations to the active site of the nuclease. The authors should conduct DNA cleavage assays in the presence of Mg2+ and Ca2+ to strengthen their conclusions.

We thank the reviewer for highlighting the important role of Ca^{2+} in Cas9 catalysis. We agree that Ca^{2+} is widely reported to reduce or inhibit DNA cleavage efficiency compared to Mg^{2+}.

Our conclusion that Ca^{2+} does not significantly distort the HNH active-site geometry is based on structural and spectroscopic evidence. In particular, NMR titration experiments (Fig. 4 and ***Supplementary Fig. 21***) show that Mg^{2+} and Ca^{2+} induce highly similar chemical shift perturbations (CSPs) across the HNH domain, indicating that the overall conformational ensemble is preserved. Consistently, the pK_a of the catalytic residue H840 remains essentially unchanged (6.77 in Mg^{2+} vs. 6.86 in Ca^{2+}, Fig, 5c), suggesting that the local electrostatic environment of the HNH active site is largely maintained.

Regarding the reviewer's suggestion to perform cleavage assays in both Mg^{2+} and Ca^{2+} conditions, we note that the effect of Ca^{2+} on Cas9 cleavage has been extensively characterized in prior biochemical studies (Jinek et al., Science 2012; Dagdas et al., Sci. Adv. 2017). These biochemical studies show that Ca^{2+} supports cleavage of the DNA target strand (TS) by the HNH domain, while cleavage of the non-target strand (NTS) by the RuvC domain is impaired. As a result, overall double-strand DNA cleavage is reduced compared to Mg^{2+} conditions. These observations are consistent with our findings that Ca^{2+} can support HNH conformational activation and

preserve active-site organization, while remaining functionally distinct from Mg^{2+} in enabling coordinated DNA cleavage.

To clarify this distinction, we have revised the manuscript to explicitly state that preservation of HNH active-site organization in the presence of Ca^{2+} does not imply equivalent catalytic behavior relative to Mg^{2+}, particularly with respect to coordinated double-strand cleavage (***page 17, lines 367-369***).

4. If the cryptic metal binding pocket is functionally important, the coordinating residues are expected to be more conserved than the surrounding residues. Is it true in the multiple sequence alignment analysis among Cas9s?

To address this point, we performed a multiple sequence alignment (MSA) of SpCas9 with representative Cas9 orthologs, including *Streptococcus thermophilus* (StCas9), *Staphylococcus aureus* (SaCas9), *Campylobacter jejuni* (CjCas9), *Neisseria meningitidis* (NmeCas9), and *Geobacillus stearothermophilus* (GeoCas9) to evaluate the conservation of residues defining the cryptic divalent metal binding pocket (DBP).

The analysis shows that while the DBP is not defined by strict sequence invariance, several key residues exhibit strong conservation or conservation of physicochemical properties. In particular, acidic residues such as D54 and E57 are highly conserved across orthologs, supporting a potential role in coordinating divalent cations. Other positions (e.g., T13, N14, Q920, and E923) display variability at the sequence level but retain polar or charged character, preserving a local environment compatible with ion binding.

Importantly, when compared to neighboring regions, the DBP is relatively enriched in conserved acidic and polar residues, rather than showing random variability. This pattern suggests that the functional features of the pocket are preserved at the level of chemical properties rather than strict sequence identity, which is consistent with a role in metal-mediated conformational regulation.

We have added a concise summary of this analysis in the revised manuscript (***pages 11-12, lines 237-240***), and the corresponding data are presented in a new ***Supplementary Fig. 20.***

Minor point:

Line 261: “in the” was repeated twice.

This minor point has been fixed.

Reviewer #2 (Remarks to the Author):

In the submitted manuscript, the authors examined the role of metal ions in mediating Cas9 cleavage activity. Largely through computational simulations, they identified a Divalent ion Binding Pocket (DBP) at the interface of the enzyme's two nuclease domains, which involves residues from the non-target DNA strand, the RuvC domain, and the L2 linker. Part of the presented results is already known in the literature (for example, it is known that Co2+ can facilitate transition of the HNH domain to an active docked state but does not support DNA cleavage). Nevertheless, I think there is still some scientific value in the present manuscript. Below are some comments that might help improve the work further.

Major comments:

1. The authors chose to focus on Mg2+, Ca2+, and Co2+. But these metal ions have already been studied experimentally. I would be interested to learn more about other ions. For example, how about monovalent or trivalent cations? If conformational activation is based purely on ionic charge, I would expect higher concentration of monovalent ions or lower concentration of trivalent ions to facilitate conformational activation too.

See response to point 3 below.

2. Similarly, how about other divalent cations that are more extreme in their ionic radius? For example, beryllium and radium in group 2 of the periodic table.

See response to point 3 below.

3. The authors can also assess other transition metals like Mn2+ and Zn2+.

We thank the reviewer for raising these important questions regarding the ion dependence of the Cas9 activation mechanism. In response, we have significantly expanded our analysis to include a broader panel of metal ions spanning monovalent, divalent, and trivalent classes. Specifically, we now consider Li^{+} and Al^{3+} (also enabling complementary solution NMR experiments), as well as additional divalent ions including Be^{2+}, Mn^{2+}, Zn^{2+}, and Ra^{2+}, as suggested by the reviewer.

To characterize ion-dependent conformational responses of the HNH domain, we performed new molecular dynamics (MD) simulations on the pre-active Cas9 structure (PDB: 5F9R) in the presence of these ions under high (10 mM) and low (10 μM) concentration conditions. Each system was simulated in three independent replicas of ~1 μs each, resulting in an additional ~36 μs of sampling. Conformational responses were quantified using two structural observables reporting on catalytic positioning and domain movement, together with a joint metric ("activation probability") that captures the extent to which these motions occur concurrently (new ***Supplementary Fig. 13***, details in Supplementary Methods).

We observe that increasing the concentration of the monovalent ion Li^{+} does not promote activation-like conformations, as both catalytic positioning and global HNH domain movement remain largely unchanged across conditions (new ***Supplementary Fig. 13***). To experimentally support these observations, we performed NMR HSQC titrations of the HNH–L2 construct in the presence of Li^{+}, which show minimal perturbation of the HNH–L2 region even at high ion concentrations (new ***Supplementary Fig. 21***). In contrast, divalent ions favor sampling of activation-like configurations, promoting both catalytic residue positioning and domain movement toward the scissile phosphate, with an associated increase in the probability of concurrently sampling these states (new ***Supplementary Fig. 13***). While this effect is consistently observed across divalent ions, variability is present in the degree of coupling between local and global motions. Trivalent ions, exemplified by Al^{3+}, alter this balance. In simulations, catalytic positioning is observed, whereas domain movement exhibits a smaller average shift accompanied by substantial variability across replicas. Consistently, NMR HSQC titrations with Al^{3+} reveal substantial spectral perturbations throughout all of HNH–L2 (new ***Supplementary Fig. 13***). These results suggest that highly charged ions can perturb the conformational landscape in a manner that differs qualitatively from divalent ions.

To complement these structural and dynamical observations, we additionally performed Cas9 DNA cleavage assays in the presence of Mg^{2+}, Li^{+}, and Al^{3+} (new ***Supplementary Fig. 14***). Mg^{2+} supported efficient DNA cleavage, whereas Li^{+} and Al^{3+} did not support productive cleavage activity under the tested conditions. These findings further indicate that productive Cas9 activation and cleavage are associated with divalent ions.

Together, these results indicate that a divalent charge promotes coordinated sampling of activation-like conformations in Cas9, in agreement with single-molecule experiments (Dagdas et al., Sci. Adv. 2017), whereas the monovalent ion Li^{+} does not and the trivalent ion Al^{3+} perturbs this balance.

We note that the mono- and trivalent analyses presented here are based on representative ions (Li^{+} and Al^{3+}, respectively). Accordingly, while these results highlight clear differences relative to divalent ions under the conditions tested, a broader analysis across additional mono- and trivalent cations would be required to establish the generality of these effects and was not systematically explored in the present study.

Finally, we note that classical fixed-charge force fields do not explicitly capture electronic polarization or charge transfer, which can be particularly relevant for ions with higher charge density or more specific coordination preferences (e.g., Be^{2+}, Al^{3+}, Zn^{2+}) (Li & Merz, Chem Rev 2017). Accordingly, results for these ions are interpreted qualitatively, focusing on relative trends rather than absolute quantitative differences.

These additional data are now presented in a new section of the Supplementary Results (Section S2.1, "Ion-dependent modulation of HNH conformational activation") and are also summarized in the main text (***pages 10-11, lines 210-216; page 13, lines 268-271***). In addition, the Methods section has been revised to include the expanded simulation set (***page 24, lines 512-519***), and two new sections describing methods and analysis have been added to the Supplementary Methods (Sections S1.3 and S1.4).

4. Can Ca2+ replace Mg2+ in in vitro DNA cleavage assays at multiple target sites?

As discussed in our response to Reviewer 1 (point 3), Ca^{2+} can partially substitute for Mg^{2+} *in vitro*, with domain-specific effects. Indeed, Ca^{2+} supports HNH conformational activation and is compatible with target strand (TS) cleavage, whereas cleavage of the non-target strand (NTS) by the RuvC domain is impaired (Dagdas et al., Sci. Adv. 2017). Consistent with these observations, our NMR titration data (***Supplementary Fig. 21***) show that Mg^{2+} and Ca^{2+} induce highly similar chemical shift perturbations across the HNH domain, indicating comparable conformational responses. The measured pK_a of the catalytic H840 is also largely unchanged (6.77 for Mg^{2+} vs. 6.86 for Ca^{2+}, Fig, 5c) suggesting that the local electrostatic environment of the active site is preserved.

Additional discussion is provided in our response to Reviewer 1 (point 3).

5. The authors performed some experimental validations using three Cas9 variants bearing mutations in the DBP. However, they should include a negative control - mutate residues that are not in the DBP and don't look important. One possibility is that numerous amino acids are important and the authors could have picked any random set of residues and have most of them affect HNH/ RuvC activity.

The mutations introduced in this study were designed based on a specific structure-function hypothesis, namely that residues lining the divalent binding pocket (DBP) contribute to ion-dependent conformational activation of the HNH domain. Accordingly, the selected variants were not intended as a general mutagenesis screen, but as targeted perturbations of residues predicted to participate in metal-mediated interactions within the DBP.

To further assess the role of these mutations, we performed additional MD simulations of our three mutants (M1, M2, M3) under high Mg^{2+} conditions. These simulations show that the variants reduce the formation of Mg^{2+}-mediated bridging interactions between the RuvC domain, the non-target strand (NTS), and the L2 region (new ***Supplementary Fig. 29***). In particular, the M2 variant, which displays the strongest reduction in DNA target strand cleavage activity (Fig. 4), exhibits markedly reduced occupancy of these interactions, accompanied by altered positioning of the HNH domain relative to the scissile phosphate. Additionally, isothermal titration calorimetry experiments conducted with full-length WT, M1, M2, and M3 SpCas9 proteins and Mg^{2+} reveal detectable Mg^{2+} binding to WT SpCas9, while M1-3 disrupt this binding (see response to Reviewer 4, point 6).

These observations support the interpretation that perturbation of the DBP affects HNH conformational activation and is associated with disruption of the specific ion-dependent regulatory network within the DBP, rather than nonspecific destabilization of the Cas9 scaffold. We have now incorporated these additional simulation results and their discussion into the revised manuscript (***page 16, lines 340-345***).

6. How rapid is the formation and disappearance of the DBP? Cryo-EM can capture different conformational states of a macromolecule if there are sufficient particles. If the DBP is absent in all published cryo-EM data of SpCas9, then presumably the DBP must be very transient.

We thank the reviewer for this important point. Cryptic binding pockets are transient conformational features that emerge dynamically and are often weakly populated within the broader conformational ensemble. Extensive computational and structural biology literature has shown that such pockets can remain difficult to resolve using experimental structural approaches, particularly when the corresponding conformations are short-lived or sparsely populated (see REFs 33-36).

To address this point quantitatively, we performed a Markov state model (MSM)-based analysis of the structural lifetime of the DBP using the MD simulations. The resulting analysis (new ***Supplementary Fig. 19***) estimates

an ensemble-level DBP lifetime on the order of ~100 ns. Specifically, the mean first passage time from pocket-containing to disrupted-pocket states was estimated to be ~95 ns using bootstrapped MSM analysis.

These results indicate that the DBP is dynamically formed and transiently maintained on nanosecond timescales, consistent with the behavior generally associated with cryptic pockets in biomolecular systems. We have now clarified this point in the revised manuscript (***page 11, lines 230-234***) and added the corresponding MSM analysis and methodological details to the Supplementary Information (Section S1.7).

7. Are the authors' results generalisable to engineered SpCas9 variants like xCas9, SpG/SpRY, and SpCas9-NG?

The engineered variants mentioned by the reviewer (e.g., xCas9, SpCas9-NG, SpG, and SpRY) are all derived from the SpCas9 scaffold and primarily contain mutations at the interface with the Protospacer Adjacent Motif (PAM), within its interacting domain and/or in the recognition (REC) lobe to alter PAM recognition and targeting scope. In contrast, the residues forming the divalent binding pocket (DBP) identified in our study are located at the HNH–RuvC interface and within the L2 region and are preserved in these engineered variants.

Accordingly, we expect that the ion-dependent conformational activation mechanism described here is likely maintained across these SpCas9-derived systems. At the same time, we note that the present study specifically focuses on wild-type SpCas9, and direct characterization of engineered variants will be required to fully establish the extent to which the same regulatory mechanism operates in each case.

Minor comments:

8. The premise of the present study is that cells can have highly variable ion concentrations - please provide relevant citations. What concentration ranges of Mg2+ and other divalent cations have been observed in various cell types?

We thank the reviewer for this insightful suggestion. We agree that providing additional context regarding intracellular divalent ion homeostasis strengthens the biological motivation of this work. Accordingly, we revised the Introduction (***page 3, lines 52-55***) and included references discussing Mg^{2+} and Ca^{2+} transport, buffering, compartmentalization, and signaling across bacterial and eukaryotic systems. Specifically, we included references covering bacterial Mg^{2+} homeostasis and adaptation to environmental changes (Groisman et al., Annu. Rev. Genet. 2013), cellular magnesium regulation in mammalian systems (Romani, Arch. Biochem. Biophys. 2011), calcium signaling and intracellular compartmentalization in eukaryotic cells (Clapham, Cell 2007), and divalent cation signaling in immune cells (Chaigne-Delalande & Lenardo, Trends Immunol. 2014). Together, these studies support the notion that the availability of divalent ions can vary across organisms, cell types, and physiological conditions, thereby influencing protein–nucleic acid interactions and catalytic activity.

9. The authors indicated D839 and N863 as coordinating residues of Mg2+. How about D861?

We thank the reviewer for this point. In cryo-EM structures of catalytically competent Cas9 (PDBs: 6O0Y, 7S4X), the coordination environment of the catalytic Mg^{2+} ion involves D839 and N863. In these structures, D861 is oriented away from the catalytic Mg^{2+} ion. This active-site configuration is maintained in our simulations of the active state.

10. Define PSCI (scissile phosphate) on first mention in the main text.

This minor point has been fixed.

Reviewer #3 (Remarks to the Author):

The manuscript entitled "A Cryptic Binding Pocket Regulates the Metal-Dependent Activity of Cas9" investigates how divalent metal ions modulate Cas9 HNH activation and cleavage through molecular simulations, Markov State Models, QM/MM and free energy calculations, and biochemical assays. The authors report that high concentrations of divalent cations facilitate the HNH conformational transition into its catalytically active state, whereas low concentrations hinder this shift. They further propose that divalent ions interact with a dynamically forming, previously uncharacterized "cryptic divalent metal–binding pocket" (DBP) located at the HNH–RuvC interface, which they suggest plays a regulatory role in coordinating HNH activation and nuclease domain communication.
Overall, the study is ambitious, technically diverse, and contributes valuable insights into metal-dependent regulation within CRISPR/Cas9 catalysis. The manuscript would, however, benefit from clarifications in several areas as outlined below.

1. The authors performed molecular dynamics simulations using high concentrations of divalent metal ions (Mg2+, Ca2+, Co2+) explicitly in their systems. which can create strong site-specific coordination. So, there can be a possibility that stable divalent metal ion binding to certain cites (like the DBP, which is one of the main focus in the paper) is a result of electrostatic screening and slow binding–unbinding kinetics due to high charge density of divalent ions. Especially, the pocket appears to be derived from transient ion–residue contacts in MD simulations, and not from experimentally validated structures and given the high negative charge of pocket residues, transient cation interactions are expected. This point must be discussed.

The reviewer correctly points out that transient interactions between divalent cations and negatively charged residues are expected in highly charged biomolecular systems such as Cas9–RNA–DNA complexes, particularly at elevated ion concentrations. However, several observations suggest that the divalent metal binding pocket (DBP) identified in our study reflects a functionally relevant conformational feature.

Solution NMR experiments performed on the isolated HNH–L2 construct show that Mg2+ induces localized structural perturbations within the HNH–L2 region, with the largest effects observed near the catalytic residue H840 and residues proximal to Q920 in the L2 region (***Supplementary Fig. 21***), consistent with the computationally identified ion-dependent regulatory network. To further investigate the ion dependence of these effects, we performed expanded simulations including mono-, di-, and trivalent ions, which revealed qualitatively distinct ion-dependent behaviors (see also the response to Reviewer 2, points 1-3). While divalent ions promote coordinated sampling of activation-like conformations, the monovalent ion Li^+ does not induce comparable structural responses, and the trivalent ion Al^{3+} perturbs the conformational landscape differently (new ***Supplementary Fig. 13***). Consistently, Li^+ induces minimal perturbations in NMR experiments even at high concentration, whereas Al^{3+} produces broader spectral perturbations throughout all of HNH–L2 (new ***Supplementary Fig. 21***). These observations support the notion that the observed effects arise from ion-dependent modulation of the conformational landscape and cannot be explained solely by electrostatic stabilization within the DBP.

In addition, newly performed simulations of the M1-M3 variants demonstrate that perturbation of residues lining the DBP specifically reduces Mg^{2+}-mediated bridging interactions between the L2 region, RuvC, and the DNA non-target strand, accompanied by altered HNH positioning relative to the scissile phosphate (new ***Supplementary Fig. 29***). Consistently, biochemical experiments reveal differential effects on HNH and RuvC catalysis (Fig. 4), while DSF, NMR, and nucleic acid binding measurements (new ***Supplementary Figs. 25, 26***) indicate that the mutations do not substantially disrupt global protein stability or nucleic acid binding.

Finally, simulations performed at intermediate Mg^{2+} concentration (2 mM) preserve the same conformational mechanism, although with reduced DBP occupancy and fewer ion-mediated interactions relative to 10 mM conditions (new ***Supplementary Fig. 18***).

Together, these findings support a model in which divalent ions modulate a dynamically formed and functionally relevant regulatory pocket, rather than producing effects that can be explained solely by electrostatic stabilization within the DBP. We have now clarified this point throughout the paragraph entitled "*Divalent Ions Drive the HNH*

Transition Through a Cryptic Binding Pocket" and added a corresponding statement in the revised manuscript (***page 13, lines 276-279***).

2. The manuscript states that Ca^{2+} maintains coordination geometries and energy barriers similar to Mg^{2+} in the HNH active site. If this were the case, SpCas9 would be expected to display catalytic activity in the presence of Ca^{2+}. However, in literature it is known that Cas9's catalytic chemistry requires Mg^{2+} in both nuclease active sites (HNH and RuvC), which contradicts this observation. Authors must provide an explanation regarding that.

The reviewer raises an important point, which is also discussed in our responses to Reviewer 1 (point 3) and Reviewer 2 (point 4).

Previous biochemical studies (e.g., Dagdas et al., Sci. Adv. 2017) have shown that Ca^{2+} supports conformational activation of the HNH domain and cleavage of the DNA target strand (TS) by HNH, but does not support RuvC-dependent cleavage of the non-target strand (NTS). As a result, overall double-strand DNA cleavage is reduced under Ca^{2+} conditions compared to Mg^{2+}. Our study investigates the role of Ca^{2+} in HNH activation and active-site organization, indicating that Ca^{2+} preserves key structural features of the HNH active site and can support TS cleavage. This finding is in line with biochemical studies and does not suggest that Ca^{2+} enables fully coordinated double-strand DNA cleavage by Cas9.

We agree that the distinction between HNH and RuvC catalysis was not sufficiently emphasized in the original manuscript. We have now clarified this point in the revised text (***page 17, lines 367-369***). Additional discussion is also provided in our responses to Reviewer 1 (point 3) and Reviewer 2 (point 4).

3. The QM/MM simulations involve ~20 ps sampling. Is the sampling sufficient to capture activation barriers for metal-dependent transition states? The short (~20 ps) may not fully capture long-timescale catalytic events. Please comment.

We thank the reviewer for raising this important point regarding the sampling of the QM/MM simulations.

Chemical reactions are rare events on the timescales accessible to *ab initio* QM/MM MD simulations, which are typically limited to the picosecond regime due to the computational cost associated with the explicit treatment of the electronic structure. As a result, spontaneous sampling of catalytic events through unbiased QM/MM MD simulations is generally not feasible. For this reason, the catalytic reaction was investigated using free-energy methods, including thermodynamic integration and metadynamics, which are designed to characterize reaction pathways and activation barriers beyond the timescales directly accessible to standard QM/MM MD.

In this approach, the relevant criterion is not the absolute trajectory length itself, but rather the convergence of the computed free-energy profiles along the chosen reaction coordinate. To assess convergence, we performed forward and backward thermodynamic integration calculations and evaluated hysteresis between the corresponding free-energy profiles (***Supplementary Figs. 34-36***). In addition, force convergence analyses were carried out for the thermodynamic integration windows (***Supplementary Fig. 24***), and QM/MM metadynamics simulations yielded converged free-energy profiles (***Supplementary Fig. 26***). Together, these analyses indicate that the calculated activation barriers are well converged within the adopted QM/MM approach. We have clarified this point in the Supplementary Methods, where the convergence analyses and enhanced sampling protocols are described.

4. In Figure 3e, the x-axis label is "Time/ns," while in Figure 3f, it is "Time (s)." The authors should standardise the formatting to avoid confusion and ensure consistency across panels.

We have corrected the x-axis in Fig. 4e (previously Fig. 3f).

5. Page 9, line 185, meaning of PSCI must be defined on its first time use.

We have now defined the acronym P_{SCI} (scissile phosphate) at its first occurrence in the manuscript.

Reviewer #4 (Remarks to the Author):

This work focuses on how divalent metal ions, like magnesium (Mg^{2+}), control the activity of Cas9, a protein essential for gene editing. Cas9 is a metal-dependent enzyme that modulates its ensemble to become fully active and cut DNA. Using computer simulations, Markov modeling and enzymatic assays, the researchers find that divalent metals (Mg^{2+}, calcium [Ca^{2+}], and cobalt [Co^{2+}]) help activate the HNH domain of Cas9. This activation is a consequence of metal binding to a 'cryptic' spot called the divalent metal binding pocket (DBP) located between the domains HNH and RuvC. When metal ion concentrations are high, the interaction of the metal ion with the DBP brings together distant regions thus enabling catalysis. If residues in this DBP are mutated, Cas9's ability to cut DNA is significantly reduced, showing that the DBP is a key regulator. The study also shows that while any of these ions can induce structural changes bridging distant sites together, the actual process of cutting the DNA strand is specific to the metal. Mg^{2+} and Ca^{2+} support effective DNA cleavage. In contrast, Co^{2+} blocks the cutting process by modifying the geometry of the active site, resulting in a significantly slower reaction.

While the approach is interesting and so are the simulations, I have several critical concerns that require additional experiments, simulations and reasoning. Specifically, the authors appear to look upon mutations through a narrow lens of activity, without suitably ruling out various other outcomes and are required to objectively evaluate the manuscript. The comments are listed below. Hence, I think the manuscript will be suited to a journal of lower profile like Biophysical Journal or JPCB, and not this venue.

Major Concerns:

1. While the molecular simulations appear to be extensive, they discuss it in terms of total aggregate time which is misleading. This should be corrected to represent the simulation time for a given condition and then indicating that such a simulation was done as duplicates or triplicates. This information is not clear in the main text.

We thank the reviewer for this important comment. We agree that reporting only the aggregate simulation time may obscure the sampling performed for individual simulation conditions.

In the revised manuscript, we explicitly state that each condition was simulated using three independent ~1 μs replicas, and we detail the corresponding ion conditions, mutant systems, and conformational states studied. The aggregate simulation time is reported only as an overall measure of the total sampling performed across all systems. We have revised the Results section accordingly (***page 10, lines 199-200***), while complete information regarding the number, duration, and setup of all trajectories is now provided in the Methods section (***pages 23-24***) and in the Supplementary Methods (***pages S2-S3***).

2. The authors do observe several partially structured states in their simulations and map them using MSMs. However, very little attention is devoted to the timescale of the different structural interconversions and their comparison with experiments that the authors themselves cite (references 17 and 18 in the main text). The interconversion timescales captured by simulations will be in the order of tens to hundreds of nanoseconds while the experiments report on millisecond timescales of interconversion (I am not talking about the catalytic rate here). This 6-order difference in the observed rates is neither acknowledged nor discussed. In other words, is it even reasonable to use MD simulations of such large systems to frame hypothesis when the timescale differences differ by orders of magnitude? The reviewer is also of the opinion (justifiably) that 1-4 microsecond runs are too short to learn anything reliable from simulations, despite using MSMs, for such large systems.

We thank the reviewer for this important comment. We agree that direct atomistic MD simulations of large biomolecular systems are typically limited to nano-to-microsecond timescales, whereas experimentally observed conformational interconversion processes may occur on substantially slower timescales.

The goal of the Markov state model (MSM) approach is precisely to infer long-timescale conformational kinetics from ensembles of shorter atomistic trajectories by identifying metastable states and estimating transition probabilities between them (see REFs 25-26, 29-31). MSM-based approaches are extensively used to investigate slow conformational transitions in large biomolecular systems for which direct millisecond-timescale MD sampling is computationally inaccessible.

In the present work, the MSM was constructed from an aggregate ensemble of approximately 4 µs of atomistic MD simulations. Transition Path Theory analysis of the resulting MSM yields an estimated A→B interconversion rate on the order of ~10^2 s^{-1}, using flux (Fig. 2) and lag time (Supplementary Fig. 5c). Flux is the probability mass moving between states as observed in our simulation, and lag time is the discrete observation window chosen for the MSM. To assess the robustness of the model, we evaluated the convergence of the implied timescales (ITS) across a range of lag times (Supplementary Fig. 5c). The observed plateau indicates that the slow conformational processes captured by the MSM are adequately separated from fast local fluctuations. In addition, Chapman–Kolmogorov tests (Supplementary Fig. 5d) show good agreement between MSM-predicted and observed transition probabilities, supporting the Markovian behaviour and predictive reliability of the model.

Experimental studies probing the corresponding conformational transition report forward rates on the order of ~1.5 s^{-1} (Dagdas et al. *Sci. Adv.* 2017). While the simulated kinetics are faster than the experimental estimates, the difference corresponds to an effective free-energy barrier deviation of only ~2-3 kcal mol^{-1}. A deviation of this magnitude is well within the expected uncertainty of atomistic force fields and MSM-derived kinetics and is consistent with observations reported in the MD/MSM literature (see REFs 25-26, 29-31).

Importantly, the conclusions of this work do not rely on exact quantitative agreement between simulated and experimental interconversion rates. Rather, the simulations are used to characterize metastable conformational ensembles, identify ion-dependent structural rearrangements, and generate mechanistic hypotheses that are independently supported by biochemical and NMR experiments.

We have now clarified these points in the revised manuscript and added discussion regarding the relationship between MSM-derived kinetics and experimentally observed conformational interconversion timescales (***page 9, lines 180-186***).

3. Continuing from above, even if one could still generate hypothesize, why is there a such a large difference in time-scales? Do simulations artificially capture some states at a faster timescale? Please justify with appropriate reasoning and provide graphs that directly compare experimental and simulation interconversion rates.

As discussed in response to point 2, MSM-based approaches are designed to infer long-timescale conformational kinetics from ensembles of shorter atomistic trajectories, and modest deviations between simulated and experimental interconversion rates are expected for complex biomolecular systems of this size.

The differences between MSM-derived and experimentally measured interconversion timescales likely reflect a combination of factors, including force-field approximations, incomplete representation of the experimental environment, and the intrinsic approximations associated with MSM-based extrapolation of long-timescale kinetics from MD sampling. Importantly, these differences do not indicate that the simulations artificially generate nonphysical conformational states. Rather, the metastable conformational ensembles identified computationally are independently supported by the biochemical and NMR experiments presented in this work.

As noted above, the difference between simulated and experimental rates corresponds to an estimated free-energy barrier deviation of only ~2-3 kcal mol^{-1}, which falls within the expected uncertainty of current atomistic MD force fields and MSM-derived kinetic models.

We have now clarified these points in the revised manuscript (***page 9, lines 180-186***).

4. The mutations M1, M2 and M3 'likely disrupts' the metal binding site. However, another and more likely possibility is that the mutations modulate the ensemble or destabilize the individual domains such that the overall cleavage activity is compromised. Do M1, M2 and M3 modulate the stability of the domains or is the domain stability unaltered? Note that this requires a careful analysis of the stability contributions from experiments and not just spectral measurements at fixed conditions (a comparison of CD spectra of WT and mutant, or just the NMR spectra will not work as the stability could still be compromised).

We thank the reviewer for this important comment. We agree that reduced catalytic activity could, in principle, arise from nonspecific destabilization of the Cas9 scaffold or modulation of the conformational ensemble, rather than from direct perturbation of the divalent binding pocket (DBP).

To address this point, we performed differential scanning fluorimetry (DSF) experiments on WT Cas9 and the M1, M2, and M3 variants under apo, Mg^{2+}-bound, and Mg^{2+}/gRNA-bound conditions (***Supplementary Fig. 25 and Supplementary Table 1***). The melting transition temperatures of all apo Cas9 variants fall within a narrow range (~40-43 °C), comparable to WT Cas9. Addition of Mg^{2+} does not produce substantial (de)stabilization of any variant, whereas guide RNA binding stabilizes all constructs by ~2-5 °C, indicating preservation of the folded architecture and assembly competence of the Cas9 complex. These observations argue against major global destabilization of the protein scaffold as the origin of the observed catalytic defects.

Importantly, the functional effects of the mutations are not uniform across the two nuclease domains. Measurements of target-strand (TS) and non-target-strand (NTS) cleavage show differential HNH and RuvC activity among the mutants (Fig. 4e-g). In particular, the mutations produce uniform reductions in RuvC-mediated NTS cleavage, while the M2 mutant, demonstrates the strongest reduction in HNH cleavage, corroborating the role of the L2 region in HNH activation. Such differential effects would not be expected from nonspecific unfolding or generalized destabilization of the Cas9 structure.

In addition, MD simulations of the M1–M3 variants show reduced formation of Mg^{2+}-mediated bridging interactions within the DBP between the RuvC domain, the L2 region, and the NTS, with M2 displaying the strongest disruption together with altered HNH positioning relative to the scissile phosphate (***Supplementary Fig. 29***). These observations support the notion that the mutations perturb the ion-dependent regulatory network associated with the DBP rather than globally destabilizing Cas9.

Consistent with these observations, temperature-dependent NMR measurements, and subsequent calculations of per-residues temperature coefficients within the HNH–L2 construct indicate preservation of the folded state, hydrogen bonding patterns, and overall structural compressibility, across the investigated temperature range in both apo and Mg2+-bound conditions (***Supplementary Fig. 26***). Using correlation analyses, these data also revealed a very small number of amino acids that may have changes to their ensemble average structure.

We have added discussion of the DSF experiments and mutant simulations in the revised manuscript (***pages 15-16, lines 325-330***) and additional information in the Supplementary Methods.

5. Page 13: The authors state "A distinct structural state was observed in the presence of Mg2+ (Supplementary Fig. 18)..". What is the distinct structural state, and how does it compare with simulations? An explicit comparison is required to objectively evaluate this. From the supporting figure, it appears that there is no change in chemical shift apart from the ion-binding sites.

We thank the reviewer for highlighting this aspect. We have revised the manuscript to clarify that the NMR experiments do not indicate a globally distinct structural state, but rather Mg^{2+}-dependent perturbations within the HNH–L2 region, indicative of binding and an altered chemical environment in their region. Importantly, the largest chemical shift perturbations are observed near the catalytic residue H840 and residues proximal to Q920 in the L2 region, consistent with the ion-dependent conformational rearrangements and regulatory network identified in the simulations (Figs. 2, 4 and ***Supplementary Fig. 21***). We have now clarified this point in the revised manuscript (***page 13, lines 260-265***).

6. What is the dissociation constant of the different metal ions to the WT and the mutants (M1, M2 and M3)? Are they the same? This set of experiments (as authors have access to NMR) is critical to know whether the binding of ions is specific or non-specific and the associated energetics (Kd), and the extent to which the mutations modulate them (if at all).

We thank the reviewer for this important point. We agree that characterization of the ion-dependent interactions associated with the divalent metal binding pocket (DBP) is important for understanding the specificity of the observed effects.

However, we note that the DBP identified in this work does not correspond to a classical buried high-affinity metal-binding site. Rather, it represents a transient cryptic pocket that dynamically emerges during conformational activation and recruits divalent ions in an ensemble-dependent manner (see also response to

Reviewer 2, point 6). Consistently, MSM analysis estimates an ensemble-level DBP lifetime on the order of ~100 ns (new ***Supplementary Fig. 19***), indicating that the pocket is only transiently populated, in line with the behavior generally associated with cryptic pockets (Refs. 33-36). As a result, the associated ion interactions are expected to be relatively weak, heterogeneous, and dynamically exchanging, making the definition of a single well-defined equilibrium dissociation constant difficult.

Nevertheless, multiple independent observations support the presence of specific and functionally relevant ion-dependent effects associated with the DBP. MD simulations show the formation of Mg^{2+}-mediated bridging interactions involving the L2 region, the RuvC domain, and the non-target strand, which are reduced in the M1–M3 variants, particularly in M2 (new ***Supplementary Fig. 29***, see also response to Reviewer 2, point 5). In parallel, NMR experiments reveal ion-dependent perturbations within the HNH–L2 region **(Supplementary Fig. 21**), while biochemical experiments demonstrate differential effects on HNH and RuvC catalysis upon mutation of DBP residues (***Fig. 4*** and ***Supplementary Figs. 23-24***). Further, isothermal titration calorimetry experiments conducted with full-length WT, M1, M2, and M3 SpCas9 proteins and Mg^{2+} (see below) show that Mg^{2+} binding to WT SpCas9 can be observed, while M1-3 disrupt this binding.

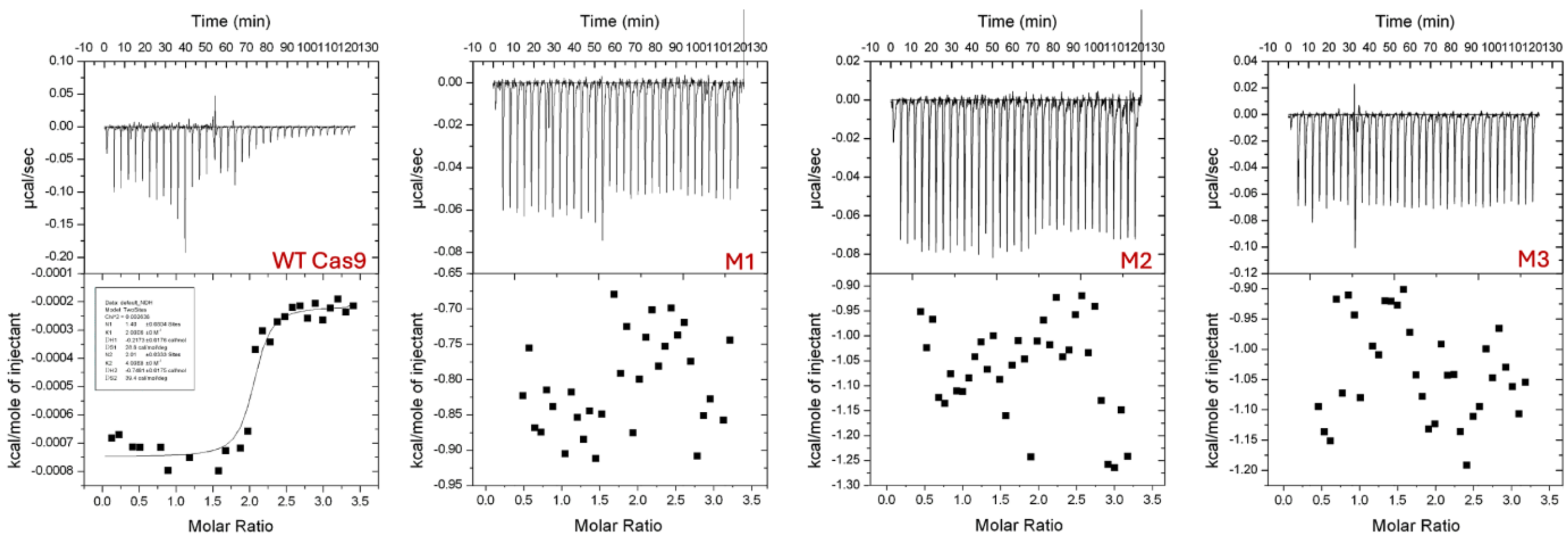

ITC analysis of Mg^{2+} binding to wild-type Cas9 and Cas9 variants M1, M2, and M3. 400 μM Mg^{2+} stock was titrated into 16 μM Cas9, and the resulting raw thermograms (top) and integrated binding isotherms (bottom) are shown. WT Cas9 was fit to a two-site binding model. The M1, M2, and M3 Cas9 variant datasets are shown for comparison, where no evidence of Mg^{2+} binding was detected with the same experimental setup as WT Cas9.

Together, these observations support the notion that the mutations perturb an ion-dependent regulatory network associated with the DBP, even though the transient and dynamic nature of the pocket complicates definition of a unique ion-binding K_d.

7. Given the complex nature of the system, it quite tricky to attribute mutations to modulate only one event. In this case, there are two additional possibilities that arise: does the binding affinity of the Cas9 to DNA remain unperturbed when mutations are introduced? Is the binding affinity to the guide RNA the same? Again, without these critical controls it is impossible to evaluate the authors' conclusions.

We thank the reviewer for this important comment. To address whether the DBP mutations alter substrate binding, we measured the affinities of WT Cas9 and the M1-M3 variants for both DNA and single guide (sgRNA) substrates.

Fluorescence anisotropy measurements of DNA binding show that all three mutants retain binding affinities comparable to WT Cas9 (new ***Supplementary Fig. 27***). In addition, sgRNA binding affinities were measured under both low (10 µM) and high (10 mM) Mg^{2+} conditions (new ***Supplementary Fig. 28***). WT Cas9 and the M1 and M3 variants exhibit comparable sgRNA binding affinities under low Mg^{2+} conditions, while M2 shows only a modest reduction in affinity. Increasing Mg^{2+} concentration weakens sgRNA binding for all Cas9 species, consistent with electrostatic screening effects arising from increased positive charge in solution.

Importantly, the observed changes in DNA and gRNA binding are relatively modest compared to the strong catalytic defects observed for the mutants, particularly for M2, which exhibits the strongest impairment in HNH cleavage activity (Fig. 4). These observations suggest that the catalytic phenotypes of the DBP mutants are not primarily caused by impaired nucleic acid binding.

We have added the discussion of these measurements in the revised manuscript (***page 16, lines 334-338***).

8. Supp. figure 19 and the associated discussion in text: Though the dynamics appear to be marginally different, it seems that the same set of residues are affected and not significantly attenuated. I also do not see error bars in this figure, and surprising given the small differences. Can a statistical analysis be provided to see if the differences are really significant?

We agree with the reviewer that a more careful look can be taken at the NMR relaxation data. In the revised manuscript, we have now incorporated the CPMG relaxation dispersion NMR data into the main text (new **Fig. 4**), showing all dispersion profiles indicative of ms motion within the figure panels. We note that Mg^{2+} binding suppresses some motion in L2 and other sites. This remains true and is likely due to the physical interaction. We have updated the figure legend to note specific residues with overlapping flexibility (via underlines) in apo and Mg^{2+}-bound samples. We also note specific flexible residues in L2 (via boxes), showing that the Mg^{2+}-bound sample has not flexibility in L2. These additions better highlight the differences between the apo and Mg^{2+}-bound datasets, particularly the attenuation of ms-timescale motions within the L2 region upon Mg^{2+} binding.

We also note in the revised text that faster timescale motions (measured by T1, T2, NOE relaxation in ***Supplementary Fig. 22***) do not have significant variation between the apo and Mg^{2+}-bound states, which is in line with the reviewer's observation.

Regarding the error bars, the extensive nature of the data collection resulted in a single-field analysis of these relaxation parameters. Thus, we did not report higher-level metrics (i.e. populations, exchange rates, diffusion tenors, order parameters) that would require multiple fields and extensive error analysis. Our intent in these studies was to establish semi-quantitative signatures of Mg2+ binding in HNH-L2, which appear primarily through chemical shifts and the changes in number/location of relaxation dispersion profiles.

9. It is also surprising that simulations/NMR are performed at 298-300 K, while cleavage reactions are at 310 K. The difference in temperatures are expected to affect the dynamics, but not justified. In other words, does the catalytic activity end up being similar if the WT/mutants are compared at 298 K? A discussion is needed.

We agree with the Reviewer that temperature can influence conformational dynamics and catalytic activity in biomolecular systems. In the present study, simulations and NMR experiments were primarily performed at 298-300 K to ensure consistency with standard solution NMR conditions and optimal spectral quality for structural and dynamical characterization of HNH from prior work.

To address the reviewer's concern, we additionally performed temperature-dependent NMR measurements of the HNH–L2 construct spanning 288–308 K under both apo and Mg^{2+}-bound conditions (new ***Supplementary Fig. 26***), including relaxation measurements (T1, T2, and heteronuclear NOE) and Mg^{2+} titration experiments at 310 K. Overall, these data show that the structural and dynamical trends observed at 298 K are preserved at higher temperatures and remain consistent with the conclusions drawn from the simulations and biochemical measurements.

We have clarified this point and added discussion of the temperature-dependent measurements in the revised manuscript (***pages 16, lines 330-334***).

Minor Point

Many instances of 'us' instead of 'microseconds' in the supporting information.

We have now introduced the notation for microseconds (μs) and introduced the abbreviation at its first occurrence in the Supporting Information.
